# Burden and Trends of Aortic Aneurysms in Individuals Aged 55 and Older from 1990 to 2021: A Systematic Analysis of the Global Burden of Disease Study 2021

**DOI:** 10.5334/gh.1517

**Published:** 2026-01-21

**Authors:** Youfu Wang, Yiming Su, Han Yang, Wenhong Jiang, Xiao Qin

**Affiliations:** 1 Department of Vascular Surgery Ward, The First Affiliated Hospital of Guangxi Medical University, Guangxi Nanning, 530000, China; 2 Department of Vascular Surgery Ward, The Fourth Affiliated Hospital of Guangxi Medical University, Guangxi Liuzhou, 545000, China

**Keywords:** aortic aneurysm, epidemiology, Global Burden of Disease, disability-adjusted life years

## Abstract

**Background:**

This study aims to assess the burden of aortic aneurysm (AA) among individuals aged 55 years and older from 1990 to 2021 at global, regional, and national levels.

**Methods:**

Data from the Global Burden of Disease (GBD) 2021 were analyzed to estimate disability-adjusted life years (DALYs), deaths, age-standardized DALYs rates (ASDR), age-standardized mortality rates (ASMR), and average annual percentage change (AAPC) associated with AA. We employed Joinpoint regression to characterize temporal trends in the AA burden, decomposition analysis to quantify the contributions of key drivers, the Slope Index of Inequality (SII) and Concentration Index of Inequality (CII) to assess health disparities, frontier analysis to benchmark development-stratified achievable disease control levels, and a Bayesian Age-Period-Cohort (BAPC) model to project AA burden trajectories through 2050.

**Results:**

Between 1990 and 2021, global deaths and DALYs due to AA among individuals aged 55 years and older increased by 73.92% and 62.57%, respectively. In contrast, ASMR and ASDR exhibited a declining trend, with AAPC values of –1.07 and –1.12, respectively. Population growth showed strong correlations with increased deaths and DALYs. SII and CII decreases indicated diminished transnational disparities in AA burden. Projections for 2050 indicate a continued rise in deaths and DALYs, while ASMR and ASDR are expected to decline further.

**Conclusions:**

Although global ASMR and ASDR for AA declined from 1990 to 2021, the absolute number of deaths and DALYs increased, with notable regional variations in disease burden. Targeted public health interventions and optimized resource allocation are essential to mitigate the burden of AA.

## Introduction

Aortic aneurysm (AA) is defined as a localized abnormal enlargement of the aorta, exceeding 1.5 times its normal diameter (1). It mainly affects individuals aged 55 years and older and is ranked as the 15th leading cause of death within this age group (2). The development of AA is often gradual, with a long asymptomatic phase, making early diagnosis challenging (2,3). As there are no effective pharmacological treatments available, surgical intervention remains the primary approach for managing AA (3). However, treatment costs are considerable. In the United States, the average cost of endovascular repair for AA is $32,052, while open repair can cost up to $36,091, creating a significant economic burden on healthcare systems (4).

Once an AA ruptures, the outcome is often fatal, with mortality rates exceeding 80% (5). Annually, about 200,000 deaths worldwide are attributed to AA (6). Although epidemiological studies on AA are limited, data from developed countries show an increasing trend in its burden. For instance, in the United Kingdom, the incidence of AA rose from 46 per 100,000 between 2000 and 2002 to 64 per 100,000 between 2017 and 2019, a trend that has been exacerbated by social inequalities (7). In the United States, the number of deaths due to AA increased by 3.39% from 2010 to 2021 (8). Despite this, there remains a lack of regional epidemiological data, particularly from developing nations. The development of AA is linked to factors such as aging, hypertension, smoking, and ethnicity (6,9). With an increasing global population and the aging demographic, the burden of AA is expected to grow. Therefore, assessing the burden of AA is crucial for the formulation of effective health policies.

In this study, the burden of AA is evaluated by examining the number of deaths, disability-adjusted life years (DALYs), age-standardized mortality rates (ASMR), and age-standardized DALYs rates (ASDR). The study also investigates the factors contributing to changes in AA burden, the variations in burden across different sociodemographic index (SDI) levels, and the effectiveness of burden control in 204 countries. Additionally, the burden of AA is projected through 2050 to anticipate future public health challenges.

## Methods

### Data source and disease definition

The GBD 2021 study assessed the epidemiological trends of 371 diseases and injuries across 21 regions and 204 countries and territories. All data are publicly available via the Global Health Data Exchange platform (https://ghdx.healthdata.org/gbd-2021/sources). Additional information on the data sources, cleaning processes, and modeling methodologies can be found in previous reports (10). In this study, data regarding the number of deaths, ASMR, DALYs, and ASDR associated with AA in individuals aged 55 and older were extracted at global, regional, and national levels (204 countries and territories). The dataset also includes 95% uncertainty intervals (UI) to indicate confidence of the estimates. Furthermore, data were collected and analyzed from age groups ranging from 55 years to 95 years and above, categorized into five-year intervals. In GBD 2021, the ICD codes associated with AA are 444.1–444.9 in the 9th edition and I71.1–I71.9 in the 10th edition (11).

### Sociodemographic index

Sociodemographic index (SDI) is a composite metric that evaluates the level of development based on education, income, and fertility rates (12). A higher SDI value signifies a more developed region. The GBD study categorized 204 geographic units worldwide into five SDI quintiles: low (0–0.454743), low-middle (0.454743–0.607679), middle (0.607679–0.689504), high-middle (0.689504–0.805129), and high (0.805129–1). In this study, the burden of AA was assessed across these SDI categories to examine how disease trends differ across regions, countries, and areas with varying SDI levels.

### Statistical analysis

To account for age structure variations, ASMR and ASDR were utilized to evaluate the burden of AA at global, SDI region, 21 GBD region, and 204 country and territory levels, with standardized rates reported per 100,000 population. This study employed Joinpoint Software (Version 5.3.0) and R software (Version 4.2.2) for data analysis. and statistical significance was set at a *P*-value of <0.05. The analytical methods applied in this paper are detailed in the subsequent subsections.

### Joinpoint regression analysis

Joinpoint regression analysis was applied to assess the temporal trends of ASMR and ASDR (13). This technique uses a log-linear regression model for piecewise regression based on temporal patterns, with P-values calculated using the Monte Carlo permutation method. Trends were quantified by computing the annual percentage change (APC) between inflection points and the average annual percentage change (AAPC) for the overall trend. A positive AAPC indicates an increasing trend (14).

### Decomposition analysis

Das Gupta’s decomposition analysis attributes the changes in disease-related deaths and DALYs to three main factors: aging, population growth, and epidemiological changes (15). This methodology has been thoroughly described in previous research (16,17). In this study, it was used to determine the key factors driving changes in the burden of AA, evaluating both the absolute and relative contributions of each factor. The absolute contribution is measured by the number of deaths and DALYs, while the relative contribution is calculated as the proportion of deaths and DALYs in 2021 relative to 1990.

### Slope and concentration index of inequality

The slope index of inequality (SII) and the concentration index of inequality (CII) were employed to evaluate cross-national variations in AA burden across five SDI levels (18). The SII, derived from regression analysis, quantifies the extent of disparities in health outcomes along a socioeconomic gradient (e.g., income), with higher SII values indicating greater inequality. CII measures how health outcomes are distributed across populations with different socioeconomic statuses, focusing on the proportional inequality. A CII closer to 0 indicates a more even distribution of health outcomes (19).

### Frontier analysis

To evaluate the gap between the actual burden and the theoretical minimum burden of AA across 204 countries and territories and to identify areas for potential improvement, a frontier analysis was conducted. This analysis utilizes data from multiple years to examine the relationship between SDI and changes in disease burden. The frontier analysis defines the theoretical minimum burden (the frontier line) and calculates the effective difference between each country’s actual burden and the frontier, with the effective difference defined as the observed value minus the theoretical minimum value. A smaller effective difference suggests that a country’s disease burden control is closer to the ideal for its SDI level (20).

### Bayesian Age-Period-Cohort model

The Bayesian Age-Period-Cohort (BAPC) framework projected longitudinal AA burden trajectories through 2050 (21). The BAPC model integrates a Bayesian inference framework to mitigate convergence and mixing challenges commonly associated with Markov Chain Monte Carlo methods. This model provides benefits such as stable long-term forecasts and the ability to generate predictions for specific age groups and age-standardized rates, making it a widely used tool in public health research (22). To evaluate model performance, we assessed prediction accuracy using the mean absolute error (MAE), mean absolute percentage error (MAPE), and fit accuracy.

## Results

### Global trends

#### Mortality

**Figure 1:**
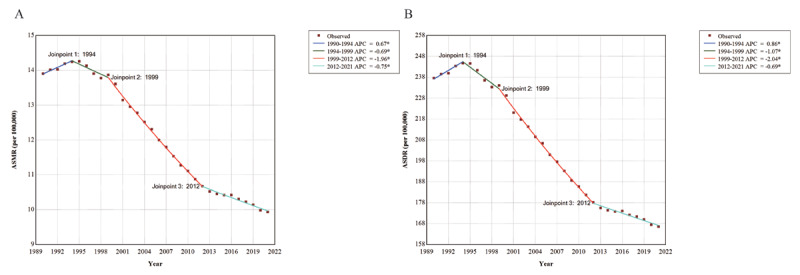
Joinpoint regression analysis of the global ASMR **(A)** and ASDR **(B)** for AA in individuals aged 55 and older from 1990 to 2021. ASMR: age-standardized mortality rate; ASDR: age-standardized disability-adjusted life years rate; AA: aortic aneurysm. *indicates that the APC is significantly different from zero at the alpha = 0.05 level.

Globally in 2021, the number of deaths due to AA amounted to 138,450 (95% UI: 123,754–149,214), reflecting a substantial increase of 73.92% (95% CI: 62.85–82.44) from 1990 to 2021. In contrast, the ASMR for AA showed a downward trend, decreasing from 13.90 per 100,000 population (95% UI: 12.74–14.78) in 1990 to 9.93 per 100,000 population (95% UI: 8.69–10.84) in 2021, with an AAPC of –1.07 (95% CI: –1.15 to –0.99) (Table 1). Joinpoint analysis revealed a significant change in ASMR in 1994. Between 1990 and 1994, ASMR rose with an APC of 0.67 (95% CI: 0.35–1.00, *P* = 0.0003) but began to decline thereafter (Figure 1).

**Table 1:** Mortality rates for AA in individuals aged 55 and older at global and regional levels, along with the associated AAPCs from 1990 to 2021.


LOCATION	1990		2021		1990–2021	
		
	CASES (95% UI)	ASMR PER 100,000 (95% UI)	CASES (95% UI)	ASMR PER 100,000 (95% UI)	CASES CHANGE (95% CI)	AACP (95% CI)

**Globe**						

Total	79607.58 (74397.50–84032.01)	13.90 (12.74–14.78)	138450.22 (123753.62–149214.49)	9.93 (8.69–10.84)	73.92 (62.85–82.44)	–1.07 (–1.15 to –0.99)

Male	51281.87 (48272.13–55304.78)	21.31 (19.62–22.98)	82322.88 (75999.88–89458.15)	13.61 (12.25–14.90)	60.53 (51.85–69.91)	–1.45 (–1.54 to –1.36)

Female	28325.71 (25536.14–31234.34)	8.72 (7.61–9.66)	56127.33 (47405.49–62050.43)	7.04 (5.84–7.92)	98.15 (79.15–113.26)	–0.70 (–0.80 to –0.59)

**SDI**						

High SDI	51071.57 (47779.66–52698.60)	26.41 (24.48–27.53)	63673.55 (54171.11–68784.57)	15.37 (13.21–16.61)	24.68 (13.49–31.39)	–1.77 (–1.85 to –1.69)

High-middle SDI	15596.83 (14863.36–16247.41)	10.23 (9.50–10.80)	30776.97 (28510.43–32911.58)	9.14 (8.24–9.89)	97.33 (82.26–111.47)	–0.36 (–0.67 to –0.04)

Middle SDI	6900.25 (6360.25–7667.24)	5.29 (4.74–5.96)	24295.67 (22015.17–26392.91)	5.96 (5.23–6.58)	252.10 (211.02–290.42)	0.40 (0.31–0.49)

Low-middle SDI	3794.10 (3001.84–5161.93)	4.70 (3.66–6.46)	14323.09 (11893.41–19198.01)	6.97 (5.68–9.35)	277.51 (213.93–345.28)	1.31 (1.20–1.42)

Low SDI	2125.00 (1286.46–3694.07)	7.33 (4.41–12.70)	5204.64 (3240.03–8583.43)	7.94 (4.83–13.05)	144.92 (100.28–199.32)	0.28 (0.12–0.43)

**GBD Regions**						

Andean Latin America	156.06 (132.58–185.43)	5.20 (4.10–6.63)	460.04 (383.35–551.60)	4.85 (3.78–6.19)	194.78 (131.33–277.43)	–0.18 (–0.66 to –0.30)

Australasia	1826.72 (1712.05–1933.55)	45.36 (39.99–50.39)	1492.29 (1298.04–1620.05)	14.45 (12.19–16.35)	–18.31 (–26.43 to –11.80)	–3.67 (–3.94 to –3.41)

Caribbean	855.56 (790.97–913.64)	21.12 (18.80–23.59)	1318.58 (1159.34–1476.95)	14.33 (12.26–16.54)	54.12 (35.95–73.16)	–1.19 (–1.61 to –0.77)

Central Asia	336.45 (289.44–402.37)	4.57 (3.88–5.53)	1234.08 (1095.70–1383.65)	10.46 (9.13–11.84)	266.79 (198.04–339.71)	2.69 (2.28–3.10)

Central Europe	3847.21 (3687.46–3977.96)	15.84 (14.88–16.67)	6213.49 (5716.40–6801.20)	15.40 (13.80–17.11)	61.51 (49.56–76.63)	–0.21 (–0.59 to –0.17)

Central Latin America	987.94 (942.54–1027.58)	8.23 (7.51–8.91)	2908.56 (2525.26–3328.30)	7.26 (6.12–8.40)	194.41 (157.08–236.30)	–0.49 (–0.89 to –0.10)

Central Sub-Saharan Africa	400.39 (217.61–667.45)	14.64 (7.58–25.62)	856.67 (477.70–1397.99)	13.02 (6.91–22.07)	113.96 (61.29–183.37)	–0.38 (–0.45 to –0.31)

East Asia	1849.72 (1512.66–2299.32)	1.57 (1.28–1.95)	7699.48 (6220.92–9524.14)	2.18 (1.74–2.71)	316.25 (192.52–474.42)	1.07 (0.88–1.26)

Eastern Europe	5640.40 (5427.65–5884.70)	12.23 (11.53–12.93)	11842.32 (10896.40–12763.24)	18.97 (17.09–20.77)	109.96 (92.54–126.98)	1.79 (0.80–2.78)

Eastern Sub-Saharan Africa	911.76 (533.71–1555.47)	9.68 (5.59–16.50)	2037.54 (1104.09–3371.79)	9.54 (5.14–15.69)	123.47 (66.78–200.84)	–0.03 (–0.14 to –0.08)

High-income Asia Pacific	4894.03 (4516.10–5125.51)	15.40 (13.92–16.44)	24857.23 (20020.26–27617.45)	23.71 (19.61–26.32)	407.91 (341.50–454.19)	1.41 (1.25–1.58)

High-income North America	18539.85 (17178.63–19326.41)	29.24 (26.73–30.79)	12703.78 (11188.78–13505.33)	10.48 (9.22–11.27)	–31.48 (–34.98 to –29.10)	–3.29 (–3.61 to –2.98)

North Africa and Middle East	766.43 (573.16–1035.34)	3.18 (2.19–4.57)	2932.09 (2522.52–3433.24)	4.59 (3.66–5.77)	282.56 (166.76–439.21)	1.23 (1.03–1.43)

Oceania	37.26 (28.44–49.94)	11.99 (8.81–16.35)	87.15 (67.88–110.86)	10.04 (7.45–13.49)	133.86 (88.29–194.57)	–0.57 (–0.77 to –0.38)

South Asia	2840.08 (1796.93–4566.73)	3.77 (2.35–6.08)	13965.31 (10035.92–20377.61)	6.57 (4.75–9.57)	391.72 (267.06–581.29)	1.85 (1.34–2.36)

Southeast Asia	1738.50 (1397.58–2180.37)	5.59 (4.37–7.21)	6540.62 (5719.64–7554.84)	7.57 (6.20–9.11)	276.22 (194.00–376.33)	0.96 (0.84–1.08)

Southern Latin America	1914.56 (1777.46–2074.10)	25.23 (21.98–28.64)	2149.33 (1971.42–2315.96)	14.00 (12.24–15.76)	12.26 (–0.14–25.04)	–1.82 (–2.01 to –1.62)

Southern Sub-Saharan Africa	586.50 (460.12–706.69)	16.03 (12.15–20.05)	995.40 (902.88–1093.07)	12.71 (10.94–14.66)	69.72 (41.49–121.68)	–0.77 (–1.04 to –0.49)

Tropical Latin America	2245.90 (2134.43–2334.05)	16.67 (15.29–17.82)	8764.08 (7980.25–9279.91)	20.74 (18.39–22.52)	290.23 (265.49–310.71)	0.78 (0.60–0.96)

Western Europe	27754.95 (26032.69–28621.87)	26.54 (24.69–27.79)	26510.00 (23076.72–28162.70)	14.11 (12.39–15.13)	–4.49 (–11.39 to –0.28)	–2.03 (–2.20 to –1.86)

Western Sub-Saharan Africa	1477.30 (780.91–2627.39)	12.84 (6.64–23.00)	2882.18 (1444.99–4848.08)	11.83 (5.90–20.06)	95.10 (45.92–144.01)	–0.26 (–0.37 to –0.14)


AA, aortic aneurysm; AAPC, average annual percentage change; ASMR, age-standardized mortality rate; SDI, sociodemographic index; GBD, Global Burden of Disease; UI, uncertainty intervals; CI, confidence interval.

Globally, the number of deaths from AA increased for both males and females, while the ASMR declined for both genders. However, the rise in deaths was more pronounced in females, while the decrease in ASMR was more notable in males. Specifically, between 1990 and 2021, male deaths increased by 60.53% (95% CI: 51.85–69.91), and female deaths rose by 98.15% (95% CI: 79.15–113.26), with AAPCs of –1.45 (95% CI: –1.54 to –1.36) and –0.70 (95% CI: –0.80 to –0.59), respectively (Table 1).

**Figure 2:**
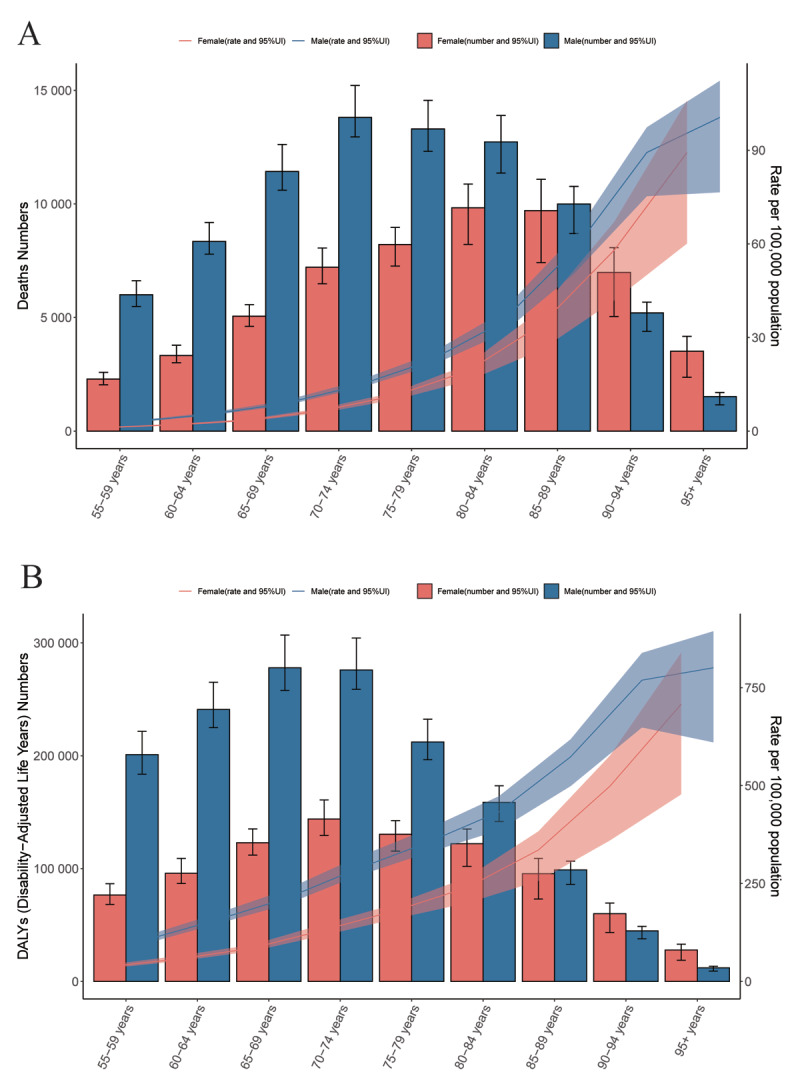
Global AA deaths and death rates **(A)**, DALYs and DALY rates **(B)** in 2021, categorized by age and gender. The blue and red lines represent the rates for males and females, respectively, with the shaded area showing the 95% uncertainty intervals. AA: aortic aneurysm; DALYs: disability-adjusted life years.

In terms of age groups, the highest number of deaths in 2021 occurred in individuals aged 80–84 years (22,557; 95% UI: 19,647–24,401). Between 2019 and 2021, all age groups saw an increase in the number of deaths, with the most substantial rise observed in females aged 95 years and older (504.17%; 95% CI: 442.71–544.92). Regarding mortality rates, both in 1990 and 2021, the rates increased with age for both males and females. In 2021, the highest mortality rate was found in individuals aged 95 years and older (92.37; 95% UI: 64.60–107.47). From 1990 to 2021, mortality rates decreased across all age groups except for those aged 95 years and older and females in this group. The most significant decline was observed in males aged 75–79 years (AAPC: –2.07; 95% CI: –2.24 to –1.91) (Figure 2, Supplementary Table 1).

#### DALYs

Between 1990 and 2021, the global DALYs attributable to AA increased by 62.57% (95% CI: 52.66–70.94), rising from 1,475,228 (95% UI: 1,399,237–1,556,633) in 1990 to 2,398,320 (95% UI: 2,194,896–2,576,074) in 2021. The ASDR demonstrated an overall decrease, dropping from 237.57 per 100,000 population (95% UI: 221.19–252.66) in 1990 to 166.53 per 100,000 population (95% UI: 149.30–181.22) in 2021, with an AAPC of –1.12 (95% CI: –1.20 to –1.04) (Supplementary Table 2). Joinpoint analysis indicated that ASDR rose with an APC of 0.86 (95%CI: 0.52–1.21, *P* < 0.0001) during 1990–1994, before beginning to decline, with the most significant decrease occurring between 1999 and 2012, marked by an APC of –2.04 (Figure 1).

From a gender perspective, the DALYs associated with AA increased for both males and females from 1990 to 2021, rising by 52.73% (95% CI: 43.87–62.52) in males and 83.11% (95% CI: 65.62–97.92) in females. The ASDR showed a declining trend, with AAPCs of –1.44 (95% CI: –1.53 to –1.36) in males and –0.73 (95% CI: –0.85 to –0.60) in females (Supplementary Table 2).

Regarding age groups, the highest DALYs in 2021 occurred in the 70–74 years age group (419,928; 95% UI: 390,321–453,272). Between 2019 and 2021, all age groups experienced an upward trend in DALYs, with the most significant increase in females aged 95 years and older (490.51%; 95% CI: 431.44–529.92). DALY rates showed an age-related increase in both 1990 and 2021. In 2021, the highest DALY rate was found in males aged 95 years and older (800.89; 95% UI: 610.56–894.13). Between 1990 and 2021, DALY rates declined across all age groups, except for the 95 years and older group. The largest decrease was observed in males aged 75–79 years (AAPC: –1.61; 95% CI: –1.54 to –1.67) (Figure 2, Supplementary Table 3).

### SDI regional trends

#### Mortality

In 2021, the highest number of deaths occurred in high SDI region (63,674; 95% UI: 54,171–68,785) and ASMR (15.37; 95% UI: 13.21–16.61) from AA. Between 1990 and 2021, death counts escalated in every SDI region, with low-middle SDI countries exhibiting the most pronounced increase (277.51%, 95% CI: 213.93–345.28). ASMR trends revealed substantial differences between high and low SDI regions. While high and high-middle SDI regions showed a decline, middle, low-middle, and low SDI regions saw increases. The most significant decline was in high SDI regions (AAPC: –1.77, 95% CI: –1.85 to –1.69), while the steepest rise occurred in low-middle SDI regions (AAPC: 1.31, 95% CI: 1.20–1.42) (Table 1).

#### DALYs

The patterns in DALYs across SDI regions closely followed the trends in deaths. In 2021, the highest DALYs was reported in high SDI region (963,980; 95% UI: 852,859–1,023,696) and ASDR (250.23; 95% UI: 223.40–266.44). However, from 1990 to 2021, DALYs increased more significantly in low- and middle-SDI regions than in high-SDI regions. Among all SDI regions, the high SDI region exhibited the least pronounced increase in DALYs (7.53%, 95% CI: 0.06–12.52) and the largest reduction in ASDR (AAPC: –2.00, 95% CI: –2.09 to –1.92). On the other hand, the low-middle SDI region experienced the most considerable rise in DALYs (260.54%, 95% CI: 197.67–329.37) and the in ASDR (AAPC: 1.25, 95% CI: 1.15–1.35) (Supplementary Table 2).

### Geographic regional trends

#### Mortality

Among the 21 geographic regions, Western Europe had the highest number of AA-related deaths in 2021 (26,510; 95% UI: 23,077–28,163), while Oceania had the lowest (87.15; 95% UI: 67.88–110.86). From 1990 to 2021, the greatest increase in deaths occurred in the High-income Asia Pacific region (407.91%; 95% CI: 341.50–454.19), while the most significant decrease was in High-income North America (–31.48%; 95% CI: –34.98 to –29.10). In 2021, the highest ASMR was recorded in High-income Asia Pacific (23.71; 95% UI: 19.61–26.32), while East Asia had the lowest (2.18; 95% UI: 1.74–2.71). At the same time, Central Asia showed the largest increase in ASMR (AAPC: 2.69; 95% CI: 2.28–3.10), while Australasia experienced the most significant decrease (AAPC: –3.67; 95% CI: –3.94 to –3.41) (Table 1).

#### DALYs

In 2021, Western Europe reported the highest DALYs related to AA (406,059; 95% UI: 365,383–427,589), while Oceania had the lowest (1877; 95% UI: 1435–2442). Tropical Latin America showed the highest ASDR (395.97; 95% UI: 358.27–427.61), while East Asia recorded the lowest ASDR (40.87; 95% UI: 32.43–51.37). From 1990 to 2021, South Asia saw the largest increase in DALYs (355.07%; 95% CI: 240.42–535.43), while high-income North America had the greatest reduction (–34.57%; 95% CI: –37.00 to –32.56). Central Asia exhibited the largest increase in ASDR (AAPC: 2.39; 95% CI: 1.89–2.89), while Australasia exhibited the most pronounced decrease (AAPC: –3.96; 95% CI: –4.46 to –3.45) (Supplementary Table 2).

### National trends

#### Mortality

**Figure 3:**
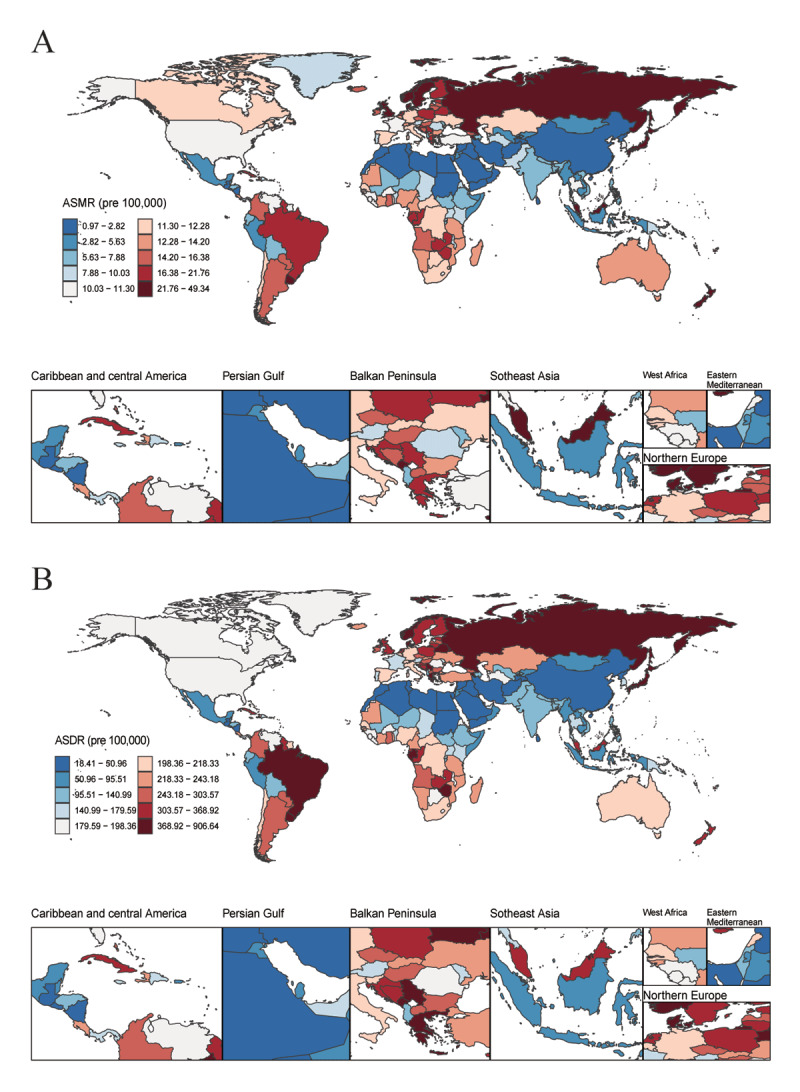
ASMR **(A)** and ASDR **(B)** of AA among individuals aged 55 and older in 204 countries and territories in 2021. ASMR: age-standardized mortality rate; ASDR: age-standardized disability-adjusted life years rate; AA: aortic aneurysm.

In 2021, Japan recorded the highest number of deaths (23,012; 95% UI: 18,379–25,666), while the Republic of Armenia had the highest ASMR (48.85; 95% UI: 40.01–58.53), and the Kingdom of Saudi Arabia recorded the lowest ASMR (0.98; 95% UI: 0.63–1.54). From 1990 to 2021, Uzbekistan experienced the largest increase in the number of deaths (926.56%; 95% CI: 560.54–1421.79) and the Republic of Yemen (845.18%; 95% CI: 510.92–1454.63). The United Kingdom of Great Britain and Northern Ireland had the largest decline in the number of deaths (–36.55%; 95% CI: –40.94 to –34.25). The Republic of Uzbekistan also showed the most substantial increase in ASMR (AAPC: 5.33; 95% CI: 4.53–6.13), while the largest decline occurred in Guam (AAPC: –4.76; 95% CI: –5.48 to –4.03) (Figure 3, Supplementary Table 4).

#### DALYs

In 2021, Japan had the highest DALYs (310,349; 95% UI: 260,095–337,781), while the Republic of Armenia recorded the highest ASDR (897.66; 95% UI: 736.65–1,078.58), and the Kingdom of Saudi Arabia reported the lowest ASDR (18.59; 95% UI: 11.92–28.73). From 1990 to 2021, the Republic of Uzbekistan (941.20%; 95% CI: 593.49–1412.90) and the Sultanate of Oman (859.56%; 95% CI: 258.95–2281.62) experienced the greatest increases in DALYs. The largest reduction in DALYs occurred in the United Kingdom of Great Britain and Northern Ireland (–46.21%; 95% CI: –49.42 to –44.31). The Republic of Uzbekistan also had the largest rise in ASDR (AAPC: 5.07; 95% CI: 4.07–6.07), while Guam saw the most significant decline (AAPC: –4.33; 95% CI: –5.19 to –3.46) (Figure 3, Supplementary Table 5).

### Aortic aneurysm burden by SDI

**Figure 4:**
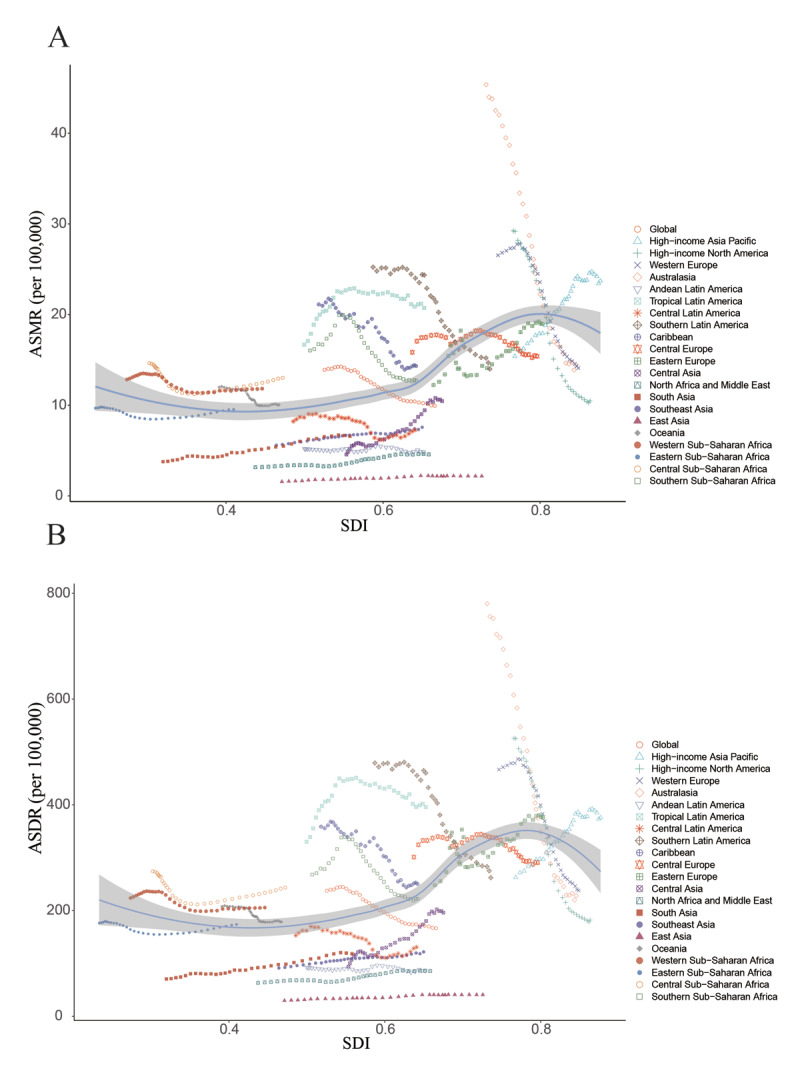
ASMR **(A)** and ASDR **(B)** of AA among individuals aged 55 and older from 1990 to 2021 across 21 regions classified by SDI. ASMR: age-standardized mortality rate; ASDR: age-standardized disability-adjusted life years rate; AA: aortic aneurysm; SDI: sociodemographic index.

An inverted S-shaped relationship was identified between ASMR and ASDR with SDI across 21 GBD regions from 1990 to 2021. In particular, ASMR (*r* = 0.4830, *p* < 0.001) and ASDR (*r* = 0.4610, *p* < 0.001) showed a positive correlation with SDI, with the highest levels of ASMR and ASDR occurring at an SDI around 0.8 (Figure 4). In 2021, a significant positive correlation was found between SDI and both ASMR (*r* = 0.3424, *p* < 0.001) and ASDR (*r* = 0.3200, *p* < 0.001) across 204 countries and territories. Noteworthy countries such as Armenia and Montenegro presented a burden considerably higher than anticipated (Supplementary Figure 1).

### Decomposition analysis of aortic aneurysm burden

A decomposition analysis of AA-related deaths and DALYs was conducted globally and across SDI regions to evaluate the contributions of epidemiological changes, population growth, and aging to the overall burden. The rise in deaths and DALYs globally and across SDI regions showed a strong correlation with population growth. Specifically, population growth contributed 147.04% to the increase in global deaths and 168.50% to the increase in DALYs. For high SDI, high-middle SDI, middle SDI, low-middle SDI, and low SDI regions, population growth accounted for 379.26%, 117.71%, 62.03%, 58.56%, and 88.08% of the increase in deaths, and 1177.01%, 132.27%, 64.52%, 60.76%, and 92.21% of the increase in DALYs, respectively. In contrast, epidemiological changes led to a reduction in global deaths and DALYs, with contribution rates of –68.47% and –80.38%, respectively. Additionally, both epidemiological changes and aging made positive contributions in middle and low SDI regions (Supplementary Table 6 and Figure 2).

### The slope index and concentration index of inequality in 1990 and 2019

The analysis of the SII and CII highlighted significant disparities in the AA burden across 204 countries and territories from 1990 to 2021. These disparities exhibited a downward trend over time. Specifically, the SII for ASMR decreased from 12.43 (95% CI: 8.70–16.16) to 6.67 (95% CI: 3.91–9.44), and the SII for ASDR dropped from 221.46 (95% CI: 154.14–288.77) to 111.26 (95% CI: 63.44–159.09). In addition, the CII for ASMR fell from 0.59 (95% CI: 0.38–0.71) to 0.39 (95% CI: 0.15–0.58), and the CII for ASDR declined from 0.56 (95% CI: 0.35–0.69) to 0.34 (95% CI: 0.11–0.53) (Supplementary Figure 3).

### Frontier analysis

A frontier analysis was conducted across 204 countries and territories using ASMR, ASDR, and SDI to evaluate the potential for reducing the AA burden. In general, countries with low to middle SDI showed a decreasing trend in both ASMR and ASDR, reflecting improvements in healthcare and public health. In contrast, trends in ASMR and ASDR in high-SDI countries have stagnated. At the country and regional levels, Armenia, Montenegro, Nauru, Monaco, Saint Lucia, Norway, Japan, and Denmark exhibited the largest effective differences in both ASMR and ASDR. Countries such as Somalia, Afghanistan, Yemen, Niger, and Timor-Leste, despite having low SDI, performed exceptionally well in controlling the disease burden. Surprisingly, high-SDI countries, including Monaco, Norway, Japan, and Denmark, showed the greatest effective disparities. Remarkably, 14 of the top 20 countries with the largest effective differences are high-SDI nations. Given their development levels, these countries have substantial potential for further reducing their disease burden (Supplementary Table 7 and Figure 4).

### Predictive analysis of aortic aneurysm through 2050

**Figure 5:**
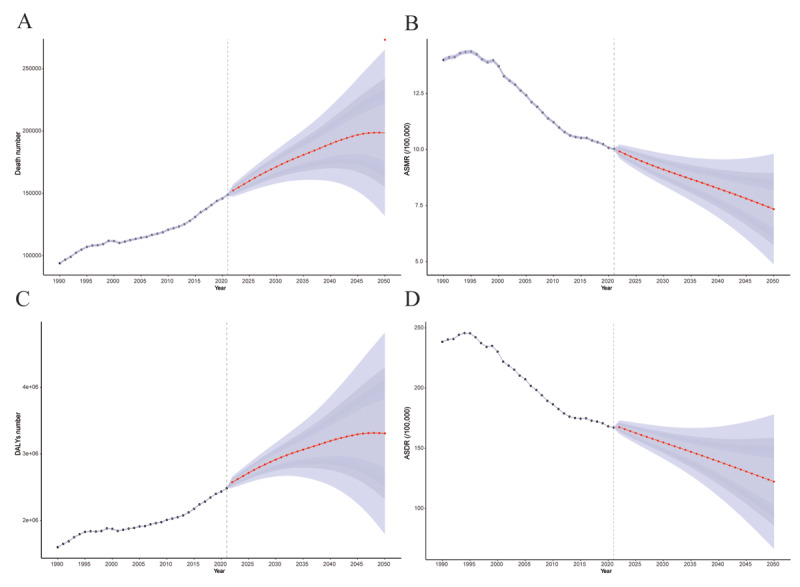
Forecasts for the number of deaths **(A)**, ASMR **(B),** DALYs **(C)**, and ASDR **(D)** for AA by 2050. The red solid line represents the projections from 2022 to 2050, with the shaded area indicating the 95% confidence interval. ASMR: age-standardized mortality rate; DALYs: disability-adjusted life years; ASDR: age-standardized disability-adjusted life years rate; AA, aortic aneurysm.

The BAPC model suggests that from 2022 to 2050, the number of deaths and DALYs will continue to increase. By 2050, AA-related deaths are anticipated to reach 198,666 (95% CI: 131,996–265,337), with DALYs totaling 3,309,386 (95% CI: 1,797,669–4,821,103), indicating rises of 43.49% and 37.99%, respectively, compared to 2021. In contrast, both ASMR and ASDR are expected to gradually decrease. By 2050, ASMR is projected to drop to 7.34 per 100,000 population (95% CI: 4.88–9.81), while ASDR is expected to reach 238.50 per 100,000 population (95% CI: 238.10–238.90) (Figure 5, Supplementary Table 8). Model performance evaluation further demonstrated good predictive accuracy, with relatively small MAE values, MAPE consistently below 12%, and fit accuracy exceeding 88%, supporting an overall satisfactory fit of the BAPC model (Supplementary Table 9).

## Discussion

This study assessed the burden and trends of AA among individuals aged 55 years and older globally, regionally, and nationally from 1990 to 2021. The results reveal that, although global ASMR and ASDR of AA have generally decreased, significant variability exists across age groups, SDI regions, and countries. Meanwhile, as the global population continues to rise, AA-related deaths and DALYs have also increased, underscoring the persistent impact of the AA burden. Moreover, the disparities observed between countries indicate that some regions have potential for improvement in disease management and intervention. These findings may support the development of targeted disease control strategies and the efficient allocation of healthcare resources.

The findings indicate that while the global ASMR and ASDR of AA have generally decreased, this trend is predominantly seen in high-SDI regions and countries, whereas middle- and low-SDI regions and countries exhibit an increasing pattern. This shift may be attributed to improvements in public health initiatives and medical technology. For instance, the United States established the AAAneurysm Outreach database in 1999, offering free nationwide screening, consultation, and recording services (23). In 2005, the U.S. Preventive Services Task Force began recommending screening for individuals aged 65–75 and those with a family history of AA (24,25). Sweden launched a nationwide AA screening program for men aged 65 and older in 2006, now covering more than 90% of men in this age group. Similar programs were later introduced by other developed nations, including the UK, Netherlands, and Norway (26,27,28). These initiatives have played a key role in reducing AA-related mortality and improving life expectancy (29,30). Additionally, smoking is a known risk factor for AA-related deaths (31). While smoking rates have been consistently falling in developed countries, they remain high in middle- and low-income countries, contributing to a decline in the AA burden in higher-income regions but a persistent burden in lower-income countries (30,32,33). Additionally, advancements in endovascular repair techniques for AA, which require expensive medical equipment, vascular stents, and specialized surgeons, make this treatment difficult to implement in low-income countries (34). For example, the United States has around 101 vascular surgeons per 10 million people, while the UK has 72.7. In comparison, countries like Morocco, South Africa, and Iran have 13.2, 10.8, and 10 vascular surgeons per 10 million people, respectively—about one-tenth of the US figure—and Ethiopia has just 0.25 (35,36,37,38,39,40). Thus, countries with lower development levels need to focus on enhancing prevention, screening, health education, professional training, and diagnostic and treatment capabilities. It should be noted that despite the declining trends in ASMR and ASDR in high-SDI countries, these values remain higher than those in low- and middle-SDI countries, possibly due to differences in screening rates and demographic factors. The frontier analysis shows that most of the top 20 countries with the largest effective differences are high-SDI countries. Although the slope index and concentration index of inequality analysis suggest that the gap in AA burden between high- and low-SDI countries has narrowed from 1990 to 2021, high-SDI countries still have significant potential for further improvement in managing AA given their higher level of development.

Additionally, it was noted that global AA-related deaths and DALYs continue to rise, a trend most noticeable in low- and middle-SDI countries. Decomposition analysis indicated that, globally, this increase is primarily driven by population growth and aging, with epidemiological changes helping to reduce the burden. From 1999 to 2020, the global population grew by about 1.8 billion, and by 2019, the number of individuals aged 65 and older surpassed 700 million. This figure is expected to represent one-fifth of the global population by 2050 (41,42). The combined effects of population growth and aging present substantial challenges to public health systems, worsening the burden of 371 diseases worldwide (11). The influence of population growth, aging, and epidemiological changes on the AA burden varies across different SDI regions. In high- and high-middle-SDI countries, population growth has worsened the AA burden, while epidemiological changes have somewhat mitigated it. Conversely, in middle-, low-middle, and low-SDI regions, all three factors have contributed to an increased burden. As previously mentioned, these disparities are linked to differences in AA screening, public health policies, resource allocation, and medical technology between SDI levels. Projections suggest that AA-related deaths and DALYs will continue to rise by 2050, presenting ongoing challenges to global public health systems.

Stratified analyses by gender, age, and region revealed notable variability. From 1990 to 2021, the decline in ASMR (AACP: –0.70, 95% CI: –0.80 to –0.59) and ASDR (AACP: –0.73, 95% CI: –0.85 to –0.60) was less prominent in females compared to males globally. Conversely, the rise in deaths (cases change: 98.15%, 95% CI: 79.15–113.26) and DALYs (cases change: 83.11%, 95% CI: 65.62–97.92) was more significant among females. This disparity may be due to screening policies in many countries that primarily focus on males. For instance, the U.S. Preventive Services Task Force recommends screening exclusively for men aged 65–75, while screening programs in the UK and Sweden are limited to men aged 65 and older (25,26,27). The exclusion of women from such screenings is typically justified by the perceived lower prevalence and cost-effectiveness (43,44,45). However, this approach has been questioned due to higher rupture rates of AA in females, which may result in underestimating their risk (46,47,48,49,50). Our findings underscore the need for AA screening to be equally implemented in women. At the age level, we observed that from 1990 to 2021, the number of AA-related deaths and DALYs increased with advancing age. This trend may be attributed to the cumulative effects of underlying conditions and other risk factors in older populations, emphasizing the significant role of aging in the growing AA burden. Regionally, AA-related deaths, DALYs, ASMR, and ASDR showed a decline only in Australasia, High-income North America, and Western Europe. This trend is likely driven by effective screening, diagnosis, advanced treatment techniques, and strong public health policies (51,52). In contrast, substantial regional differences in trends indicate an unequal distribution of the AA disease burden globally, reflecting disparities in healthcare resources, screening coverage, and management of risk factors. Therefore, prevention and control strategies should be tailored to the local sociodemographic context. In low SDI regions, expanding cost-effective screening programs, such as handheld ultrasound delivered through primary care and community outreach, may improve early detection and timely referral. In high SDI regions, screening strategies can be further optimized through risk-stratified approaches, including targeted screening among women in high-risk groups and prioritizing screening and surveillance in older adults, together with strengthened follow-up and comprehensive management of major risk factors. Overall, optimizing the allocation of healthcare resources, improving screening accessibility, and promoting evidence-based management and regional collaboration may help reduce the AA burden.

There are some limitations of our study. First, our study did not assess the incidence and prevalence of AA, which limits the overall evaluation of the disease burden. Given the often asymptomatic and gradual onset of AA, many cases remain undiagnosed until rupture, and many regions, especially those with limited healthcare resources, lack systematic AA screening programs, leading to gaps in GBD data. Second, inadequate screening and diagnostic efforts in certain regions may result in an underestimation of the disease burden, particularly in low-resource settings. Third, the absence of data on AA subtypes prevented us from evaluating the burden of different forms of AA, such as abdominal or thoracic aortic aneurysms. Finally, the frontier analysis and BAPC projections are based on historical patterns and therefore assume that no major public health crises or disruptive innovations in AA prevention, screening, or management occur during the projection period. Accordingly, the interpretation of these results should be made with appropriate caution.

## Conclusion

In summary, despite the decrease in ASMR and ASDR, the increasing global population and the aging trend have contributed to a continued rise in AA-related deaths and DALYs, highlighting the persistent impact of the AA burden. Furthermore, significant regional differences point to the unequal distribution of this burden across the globe. To address this challenge, it is vital to improve screening and early diagnosis worldwide, especially in regions with limited resources, while also implementing effective public health policies and interventions to alleviate the health burden of AA.

## Data Accessibility Statement

Publicly available datasets were analyzed in this study. The data are available from the GBD results tool (http://ghdx.healthdata.org/gbd-results-tool), and the processed data supporting the findings are provided in the Supplementary Material. For reasonable requests, the analysis code used in this study is available from the corresponding author.

## Additional File

The additional file for this article can be found as follows:

10.5334/gh.1517.s1 Supplementary Files. Tables 1 to 9 and Figures 1 to 4.
