# Supplementary material for: Burden and Trends of Aortic Aneurysms in Individuals Aged 55 and Older from 1990 to 2021: A Systematic Analysis of the Global Burden of Disease Study 2021
Source: Glob Heart. 2026 Jan 21;21(1):4. doi: 10.5334/gh.1517 (PMC12829448; doi:10.5334/gh.1517)
Supplement: Supplementary Files. — Tables 1 to 9 and Figures 1 to 4. [file gh-21-1-1517-s1.pdf]

Contents

Table 1. Mortality rates for AA in individuals aged 55 and older across various age subgroups at global and regional levels, along with corresponding AAPCs from 1990 to 2021 2

Table 2. DALYs for AA in individuals aged 55 and older at the global and regional levels, with the associated AAPCs from 1990 to 2021 3

Table 3. DALYs for AA in individuals aged 55 and older across different age subgroups at global and regional levels, along with related AAPCs from 1990 to 2021 6

Table 4. Mortality rates of AA among individuals aged 55 and older across 204 countries and territories, along with the corresponding AAPCs from 1990 to 2021 8

Table 5. DALYs of AA among individuals aged 55 and older in 204 countries and territories, with the associated AAPCs from 1990 to 2021 23

Table 6. Deaths and DALYs of AA with decomposition analysis, categorized by global and SDI regions 38

Table 7. ASMR and ASDR of AA with frontier analysis across all countries and territories 39

Table 8. Forecasts for the number of deaths, ASMR, number of DALYs, and ASDR for AA from 2022 to 2050 46

Table 9. The prediction accuracy validation of the Bayesian age-period-cohort mode 47

Figure 1. ASMR (A) and ASDR (B) of AA among individuals aged 55 and older from 1990 to 2021 in 204 countries and territories classified by SDI 48

Figure 2. Impact of aging, epidemiological changes, and population growth on AA deaths and DALYs globally and across SDI regions from 1990 to 2021 49

Figure 3. SII and CII of ASMR (A and B) and ASDR (C and D) for global AA in 1990 and 2021 50

Figure 4. Frontier analysis based on SDI, ASMR and ASDR for AA across 204 countries and territories 51

Table 1. Mortality rates for AA in individuals aged 55 and older across various age subgroups at global and regional levels, along with corresponding AAPCs from 1990 to 2021.

Characteristics	1990		2021		1990-2021	
	Cases (95%UI)	ASMR per 100,000 (95%UI)	Cases (95%UI)	ASMR per 100,000 (95%UI)	Cases change (95%CI)	AACP (95%CI)
**55-59 years**						
both	4666.17(4397.98-5074.86)	2.52(2.37-2.74)	8290.38(7597.86-9113.56)	2.09(1.92-2.30)	77.67 (59.44-95.13)	-0.54(-0.86 to 0.23)
male	3458.23(3138.71-3926.19)	3.72(3.38-4.23)	6001.98(5484.67-6616.98)	3.08(2.82-3.40)	73.56 (55.57-91.90)	-0.55(-0.86 to -0.25)
female	1207.94(1066.95-1468.85)	1.31(1.16-1.59)	2288.40(2039.54-2587.48)	1.14(1.01-1.29)	89.45 (57.10-117.67)	-0.37(-0.59 to -0.16)
**60-64 years**						
both	7783.99(7391.75-8362.96)	4.85(4.60-5.21)	11669.14(10885.96-12673.19)	3.65(3.40-3.96)	49.91 (38.33-60.51)	-0.88(-1.22 to -0.54)
male	5828.10(5409.87-6464.37)	7.42(6.89-8.23)	8345.76(7787.64-9180.67)	5.37(5.01-5.90)	43.20 (33.49-54.52)	-1.02(-1.43 to -0.60)
female	1955.89(1761.46-2292.66)	2.38(2.15-2.79)	3323.38(3006.94-3778.60)	2.02(1.83-2.30)	69.92 (47.59-89.03)	-0.52(-0.85 to -0.19)
**65-69 years**						
both	11493.10(10986.46-12197.73)	9.30(8.89-9.87)	16484.09(15355.40-17909.55)	5.98(5.57-6.49)	43.43 (33.48-52.15)	-1.4(-1.65 to -1.16)
male	8356.08(7809.23-9116.12)	14.58(13.62-15.90)	11425.80(10603.56-12616.22)	8.67(8.04-9.57)	36.74 (28.34-46.47)	-1.65(-1.84 to -1.46)
female	3137.02(2872.35-3544.85)	4.73(4.33-5.35)	5058.29(4610.31-5561.62)	3.51(3.20-3.86)	61.25 (44.21-75.77)	-0.93(-1.12 to -0.75)
**70-74 years**						
both	13329.14(12751.42-14067.54)	15.74(15.06-16.62)	21017.46(19538.19-22682.30)	10.21(9.49-11.02)	57.68 (47.58-67.12)	-1.42(-1.55 to -1.28)
male	9219.20(8676.54-9939.02)	24.51(23.06-26.42)	13805.14(12949.56-15213.96)	14.32(13.43-15.78)	49.74 (40.95-60.19)	-1.72(-1.85 to -1.59)
female	4109.94(3762.28-4604.94)	8.74(8.00-9.79)	7212.31(6484.05-8060.11)	6.59(5.92-7.36)	75.48 (57.98-91.55)	-0.91(-1.06 to -0.75)
**75-79 years**						
both	16611.64(15714.23-17398.45)	26.99(25.53-28.26)	21508.22(19620.13-23035.32)	16.31(14.88-17.47)	29.48 (21.44-35.82)	-1.62(-1.78 to -1.46)
male	10763.67(10173.78-11463.10)	42.66(40.32-45.43)	13297.35(12314.03-14557.91)	22.24(20.60-24.35)	23.54 (16.16-31.82)	-2.07(-2.24 to -1.91)
female	5847.97(5360.27-6369.39)	16.10(14.76-17.54)	8210.87(7265.50-8966.72)	11.39(10.08-12.44)	40.41 (28.20-50.82)	-1.09(-1.20 to -0.99)
**80-84 years**						
both	13469.49(12237.14-14222.85)	38.08(34.59-40.20)	22556.80(19647.35-24401.09)	25.75(22.43-27.86)	67.47 (57.35-74.86)	-1.27(-1.39 to -1.15)
male	7913.91(7306.79-8406.35)	59.58(55.01-63.28)	12726.58(11356.77-13895.22)	34.72(30.99-37.91)	60.81 (52.13-69.64)	-1.74(-1.86 to -1.61)

female	5555.57(4881.96-6005.34)	25.15(22.10-27.18)	9830.21(8218.34-10872.35)	19.30(16.14-21.35)	76.94 (62.70-89.33)	-0.86(-1.02 to -0.69)
**85-89 years**						
both	8191.72(7099.69-8742.04)	54.21(46.98-57.85)	19701.47(16222.10-21637.05)	43.09(35.48-47.32)	140.50 (124.71-151.97)	-0.72(-0.97 to -0.47)
male	4110.74(3686.26-4396.74)	81.17(72.79-86.82)	9996.57(8696.27-10773.29)	57.94(50.41-62.44)	143.18 (130.14-156.97)	-1.07(-1.28 to -0.86)
female	4080.99(3398.81-4447.90)	40.62(33.83-44.27)	9704.90(7418.96-11081.19)	34.09(26.06-38.92)	137.81 (113.79-154.98)	-0.56(-0.80 to -0.31)
**90-94 years**						
both	3187.73(2629.30-3461.58)	74.39(61.36-80.78)	12188.40(9386.06-13647.46)	68.13(52.47-76.29)	282.35 (256.29-299.23)	-0.29(-0.42 to -0.16)
male	1339.05(1160.92-1444.91)	106.36(92.21-114.77)	5204.03(4387.37-5673.87)	89.29(75.27-97.35)	288.64 (270.91-305.19)	-0.60(-0.88 to -0.33)
female	1848.68(1460.81-2036.87)	61.09(48.27-67.31)	6984.37(5046.05-8072.97)	57.91(41.84-66.94)	277.80 (240.40-299.62)	-0.17(-0.41 to 0.07)
**95+ years**						
both	874.60(664.64-976.27)	85.91(65.28-95.89)	5034.26(3520.79-5857.42)	92.37(64.60-107.47)	475.61 (427.77-508.17)	0.20(-0.10-0.50)
male	292.88(236.06-321.51)	112.55(90.71-123.55)	1519.66(1156.94-1697.07)	100.50(76.52-112.24)	418.86 (392.58-441.62)	-0.45(-0.72 to -0.17)
female	581.72(433.45-657.30)	76.76(57.19-86.73)	3514.60(2367.20-4170.68)	89.24(60.11-105.90)	504.17 (442.71-544.92)	0.44(0.24 to 0.64)

AA, aortic aneurysm; AAPC, average annual percentage change; ASMR, age-standardized mortality rate.

Table 2. DALYs for AA in individuals aged 55 and older at global and regional levels, with the associated AAPCs from 1990 to 2021.

Characteristics	1990		2021		1990-2021	
	Cases (95% UI)	ASDR per 100,000 (95% UI)	Cases (95% UI)	ASDR per 100,000 (95% UI)	Cases change (95% CI)	AACP (95% CI)
**Globe**						
total	1475228.48 (1399236.58-1556633.48)	237.57 (221.19-252.66)	2398319.66 (2194896.37-2576073.74)	166.53 (149.30-181.22)	62.57 (52.66-70.94)	-1.12(-1.20 to -1.04)
male	997137.12 (937295.79-1081925.32)	366.24 (339.12-397.23)	1522889.15 (1413511.40-1664816.28)	233.03 (212.59-255.79)	52.73 (43.87-62.52)	-1.44(-1.53 to -1.36)
female	478091.36 (432056.57-534256.71)	139.33 (123.60-156.08)	875430.51 (766730.46-964468.36)	110.42 (94.61-123.70)	83.11 (65.62-97.92)	-0.73(-0.85 to -0.60)
**SDI**						

High SDI	896446.67 (851955.63-919213.45)	463.28 (437.41-479.62)	963979.62 (852859.40-1023695.99)	250.23 (223.40-266.44)	7.53 (0.06-12.52)	-2.00(-2.09 to -1.92)
High-middle SDI	314639.73 (302310.16-328014.51)	190.74 (179.28-201.14)	578164.60 (540852.76-618522.80)	168.28 (153.63-182.04)	83.75 (68.56-98.06)	-0.27(-0.59 to -0.05)
Middle SDI	139043.21 (128624.04-155299.59)	91.76 (82.96-103.51)	466076.23 (423566.32-505897.54)	106.02 (94.15-116.89)	235.20 (193.33-274.52)	0.48(0.38-0.57)
Low-middle SDI	78036.31 (61579.97-107305.01)	86.03 (66.71-119.52)	281353.94 (234208.16-376926.65)	125.69 (103.06-169.05)	260.54 (197.67-329.37)	1.25(1.15-1.35)
Low SDI	44779.99 (27161.54-77917.68)	134.87 (81.02-234.14)	105606.19 (65459.56-174699.24)	143.29 (87.13-236.50)	135.83 (92.79-189.80)	0.21(0.06-0.37)
**GBD Regions**						
Andean Latin America	2914.00 (2438.22-3502.07)	92.25 (72.08-118.21)	8342.91 (6920.79-10100.37)	86.22 (67.03-110.54)	186.30 (120.34-273.69)	-0.19(-0.66 to -0.28)
Australasia	32079.18 (30288.80-33918.66)	780.59 (693.18-867.67)	22412.10 (19983.56-24075.12)	227.98 (195.52-257.25)	-30.14 (-36.26 to -24.87)	-3.96(-4.46 to -3.45)
Caribbean	15160.08 (14106.14-16243.45)	358.71 (320.11-401.07)	22943.78 (20226.07-25951.81)	250.39 (213.93-290.01)	51.34 (33.26-70.72)	-1.21(-1.50 to -0.91)
Central Asia	7345.66 (6378.44-8705.22)	94.05 (80.93-112.52)	25639.18 (22574.10-28910.49)	196.02 (171.10-222.94)	249.04 (185.53-317.09)	2.39(1.89-2.89)
Central Europe	77643.60 (74984.66-80121.88)	301.23 (285.52-316.11)	113914.44 (104729.96-125138.91)	290.84 (261.75-325.02)	46.71 (34.40-62.12)	-0.17(-0.49 to -0.15)
Central Latin America	19635.58 (18831.87-20400.02)	153.07 (140.41-165.85)	54006.75 (46622.31-62315.04)	130.98 (110.54-152.41)	175.05 (137.51-217.52)	-0.62(-1.03 to -0.22)
Central Sub-Saharan Africa	8914.94 (4850.56-15021.54)	274.44 (143.43-480.34)	18662.94 (10325.75-30409.13)	243.40 (129.41-411.66)	109.34 (54.95-179.82)	-0.39(-0.49 to -0.29)
East Asia	40900.13 (33094.11-51932.57)	29.80 (23.99-37.53)	154301.09 (123710.31-193835.77)	40.87 (32.43-51.37)	277.26 (158.18-433.77)	1.04(0.90-1.18)
Eastern Europe	119888.14 (115772.23-124923.85)	248.02 (235.60-261.43)	235419.75 (216564.39-254630.43)	375.65 (339.68-411.94)	96.37 (79.88-112.88)	1.67(0.87-2.47)

Eastern Sub-Saharan Africa	19124.18 (11094.15-32899.55)	176.56 (101.40-303.97)	41812.74 (22376.41-68779.90)	173.77 (92.81-286.88)	118.64 (62.31-197.91)	-0.04(-0.13 to -0.06)
High-income Asia Pacific	87665.06 (82105.39-91824.19)	262.46 (241.29-279.55)	339215.31 (286436.34-369497.87)	375.73 (325.23-409.72)	286.94 (246.83-317.11)	1.08(0.54-1.63)
High-income North America	327225.66 (309176.81-337899.72)	525.88 (490.86-549.59)	214102.20 (195517.01-224570.68)	182.35 (165.58-193.69)	-34.57 (-37.00 to -32.56)	-3.41(-3.80 to -3.02)
North Africa and Middle East	17119.21 (12621.83-23873.77)	63.61 (43.29-92.96)	60349.87 (51738.89-70830.77)	85.92 (68.32-108.46)	252.53 (137.26-409.63)	0.97(0.79-1.15)
Oceania	822.48 (614.98-1138.19)	207.87 (150.50-291.56)	1877.49 (1434.82-2441.74)	178.62 (131.06-242.52)	128.27 (79.54-194.06)	-0.48(-0.63 to -0.33)
South Asia	60281.25 (37935.92-98457.20)	70.68 (43.75-115.37)	274322.76 (196039.35-401991.22)	118.39 (85.17-173.33)	355.07 (240.42-535.43)	0.98(0.88-1.07)
Southeast Asia	32299.73 (26056.29-40521.55)	91.37 (71.82-117.59)	117485.80 (102456.19-134677.11)	121.93 (101.02-145.71)	263.74 (181.41-363.00)	1.72(1.44-2.00)
Southern Latin America	37687.28 (34979.92-41141.94)	479.22 (418.46-545.31)	39567.15 (36497.40-42739.69)	262.33 (230.88-295.49)	4.99 (-7.28-18.09)	0.92(0.76-1.07)
Southern Sub-Saharan Africa	10710.63 (8448.95-12849.52)	268.08 (204.37-335.08)	19421.10 (17564.71-21325.56)	222.25 (191.64-257.08)	81.33 (51.63-135.60)	-1.88(-2.08 to -1.68)
Tropical Latin America	48259.06 (46068.07-49987.60)	330.12 (306.11-351.56)	171843.15 (158938.06-181235.74)	395.97 (358.27-427.61)	256.08 (234.40-274.31)	-0.59(-0.90 to -0.28)
Western Europe	480708.18 (458128.22-493057.60)	466.79 (440.81-486.26)	406059.17 (365383.46-427589.00)	238.00 (214.28-253.48)	-15.53 (-20.45 to -12.08)	0.62(0.44-0.79)
Western Sub-Saharan Africa	28844.46 (15090.62-51809.23)	223.79 (115.10-404.21)	56620.00 (28133.95-96164.48)	205.66 (101.60-352.07)	96.29 (45.40-148.69)	-2.16(-2.35 to -1.97)

DALYs, disability-adjusted life years; AA, aortic aneurysm; AAPC, average annual percentage change; ASDR, age-standardized disability-adjusted life years rate; SDI, sociodemographic index; GBD, Global Burden of Disease; UI, uncertainty intervals; CI, confidence interval.

Table 3. DALYs for AA in individuals aged 55 and older across different age subgroups at global and regional levels, along with related AAPCs from 1990 to 2021.

Characteristics	1990		2021		1990-2021	
	Cases (95% UI)	ASDR per 100,000 (95% UI)	Cases (95% UI)	ASDR per 100,000 (95% UI)	Cases change (95% CI)	AACP (95% CI)
**55-59 years**						
both	156049.13 (147065.68-169736.49)	84.26 (79.41-91.65)	277658.53 (254432.49-305272.60)	70.16 (64.29-77.14)	77.93 (59.65-95.44)	-0.54 (-0.22 to -0.86)
male	115657.61 (104953.08-131334.48)	124.51 (112.99-141.39)	201029.51 (183681.02-221667.95)	103.24 (94.33-113.84)	73.81 (55.77-92.21)	-0.55 (-0.24 to -0.86)
female	40391.52 (35669.87-49131.67)	43.76 (38.64-53.22)	76629.02 (68293.42-86648.02)	38.12 (33.98-43.11)	89.72 (57.28-118.02)	-0.37 (-0.31 to -0.43)
**60-64 years**						
both	224502.49 (213176.26-241228.78)	139.78 (132.73-150.20)	336954.86 (314325.37-366000.10)	105.28 (98.21-114.36)	50.09 (38.49-60.72)	-0.88 (-0.55 to -1.21)
male	168153.67 (156067.87-186541.79)	214.08 (198.7-237.49)	241034.48 (224895.02-265180.60)	154.97 (144.59-170.49)	43.34 (33.62-54.69)	-1.01 (-0.61 to -1.42)
female	56348.82 (50735.09-66076.32)	68.67 (61.82-80.52)	95920.38 (86781.75-109068.85)	58.31 (52.75-66.30)	70.23 (47.82-89.40)	-0.52 (-0.46 to -0.57)
**65-69 years**						
both	279406.44 (267081.48-296573.05)	226.04 (216.07-239.93)	400868.60 (373392.39-435563.08)	145.33 (135.36-157.9)	43.47 (33.51-52.20)	-1.40 (-1.18 to -1.62)
male	203233.77 (189924.8-221746.02)	354.49 (331.28-386.78)	277989.58 (257969.98-306968.23)	210.86 (195.68-232.85)	36.78 (28.37-46.53)	-1.65 (-1.47 to -1.83)
female	76172.67 (69742.35-86088.66)	114.93 (105.23-129.89)	122879.03 (112000.87-135115.57)	85.33 (77.77-93.82)	61.32 (44.26-75.84)	-0.93 (-0.89 to -0.98)
**70-74 years**						
both	266313.49 (254736.74-281111.83)	314.56 (300.89-332.04)	419927.80 (390321.28-453271.74)	204.01 (189.62-220.21)	57.68 (47.57-67.15)	-1.41 (-1.29 to -1.53)

male	184341.14 (173469.03-198779.27)	490.03 (461.13-528.41)	276016.46 (258885.18-304295.66)	286.35 (268.58-315.69)	49.73 (40.92-60.19)	-1.73 (-1.62 to -1.84)
female	81972.35 (75024.39-91878.79)	174.25 (159.48-195.31)	143911.34 (129357.45-160857.95)	131.49 (118.19-146.97)	75.56 (58.02-91.66)	-0.90 (-0.85 to -0.98)
**75-79 years**						
both	264753.93 (250433.88-277337.79)	430.11 (406.84-450.55)	342678.44 (312641.05-367112.22)	259.83 (237.06-278.36)	29.43(21.36,35.80)	-1.61 (-1.54 to -1.67)
male	171781.58 (162347.68-182985.39)	680.79 (643.4-725.19)	212201.15 (196475.77-232424.10)	354.93 (328.63-388.76)	23.53 (16.14-31.82)	-2.11 (-1.98 to -2.24)
female	92972.35 (85198.89-101299.38)	255.96 (234.56-278.89)	130477.29 (115515.78-142531.66)	180.97 (160.22-197.69)	40.34 (28.12-50.78)	-1.09 (-1.04 to -1.15)
**80-84 years**						
both	168343.95 (152953.77-177768.66)	475.87 (432.37-502.51)	280832.37 (244685.42-303794.47)	320.65 (279.38-346.86)	66.82 (56.73-74.21)	-1.29 (-1.18 to -1.41)
male	99137.65 (91531.32-105317.63)	746.33 (689.06-792.85)	158766.04 (141684.71-173421.77)	433.17 (386.57-473.16)	60.15 (51.47-68.96)	-1.75 (-1.62 to -1.88)
female	69206.30 (60810.92-74819.24)	313.26 (275.26-338.66)	122066.33 (102086.34-135034.44)	239.67 (200.44-265.13)	76.38 (62.20-88.74)	-0.87 (-0.83 to -0.91)
**85-89 years**						
both	81280.61 (70459.04-86741.23)	537.89 (466.27-574.02)	194465.14 (160201.14-213520.3)	425.32 (350.38-467.00)	139.25 (123.55-150.66)	-0.74 (-0.49 to -0.99)
male	40900.98 (36679.8-43749.75)	807.67 (724.32-863.93)	98908.00 (86050.28-106607.57)	573.29 (498.77-617.92)	141.82 (128.86-155.53)	-1.09 (-0.89 to -1.29)
female	40379.63 (33633.38-44015.56)	401.91 (334.76-438.10)	95557.14 (73095.64-109080.56)	335.65 (256.75-383.15)	136.65 (112.79-153.69)	-0.58 (-0.53 to -0.64)
**90-94 years**						
both	27470.73 (22659.21-29830.10)	641.06 (528.78-696.12)	104940.21(80818.63-117499.79)	586.61(451.77-656.81)	282.01 (255.97-298.87)	-0.30 (-0.17 to -0.43)

male	11545.06 (10009.29-12457.97)	917.03 (795.04-989.54)	44834.21(37799.39-48881.99)	769.22(648.53-838.67)	288.34 (270.63-304.89)	-0.60 (-0.34 to -0.87)
female	15925.67 (12584.47-17546.81)	526.25 (415.85-579.82)	60106.00(43427.23-69473.07)	498.36(360.07-576.02)	277.42 (240.06-299.20)	-0.18 (-0.13 to -0.25)
**95+ years**						
both	7107.71 (5404.45-7929.92)	698.14 (530.84-778.90)	39993.71(28025.35-46485.54)	733.79(514.2-852.90)	462.68 (416.67-493.84)	0.17 (0.31-0.03)
male	2385.67 (1923.92-2618.27)	916.75 (739.31-1006.13)	12109.72(9231.96-13519.60)	800.89(610.56-894.13)	407.60 (382.46-429.49)	-0.53 (-0.25 to -0.81)
female	4722.04 (3520.38-5334.19)	623.08 (464.52-703.85)	27883.99(18822.93-33055.75)	708.03(477.95-839.35)	490.51 (431.44-529.92)	0.38 (0.46-0.29)

DALYs, disability-adjusted life years; AA, aortic aneurysm; AAPC, average annual percentage change; ASDR, age-standardized disability-adjusted life years rate; UI, uncertainty intervals; CI, confidence interval.

Table 4. Mortality rates of AA among individuals aged 55 and older across 204 countries and territories, along with the corresponding AAPCs from 1990 to 2021.

	**1990**		**2021**		**1990-2021**	
**Characteristics**	**Cases (95% UI)**	**ASMR per 100,000 (95% UI)**	**Cases (95% UI)**	**ASMR per 100,000 (95% UI)**	**Cases change (95% CI)**	**AACP (95% CI)**
American Samoa	0.50 (0.42-0.59)	20.43 (13.94-29.42)	0.91 (0.75-1.10)	14.20 (9.68-20.18)	80.03 (41.63-130.47)	-1.07 (-1.68 to -0.45)
Antigua and Barbuda	1.61 (1.46-1.78)	16.34 (13.85-19.20)	1.77 (1.60-2.00)	10.95 (9.18-13.16)	9.92 (-2.76-22.66)	-1.36 (-2.45 to -0.25)
Arab Republic of Egypt	61.29 (42.59-90.23)	1.79 (1.10-2.84)	183.62 (144.08-230.76)	2.26 (1.56-3.24)	199.59 (87.87-358.44)	0.81 (0.51-1.11)
Argentine Republic	1482.96 (1359.80-1626.53)	27.76 (23.52-32.33)	1398.46 (1273.65-1524.29)	14.22 (11.96-16.62)	-5.70 (-17.79-6.69)	-2.03 (-2.3 to -1.75)

Australia	1432.38 (1326.41-1529.52)	42.84 (36.93-48.75)	1143.49 (990.52-1247.34)	13.03 (10.80-15.01)	-20.17 (-28.64 to -13.02)	-3.82 (-4.36 to -3.27)
Barbados	8.68 (7.97-9.45)	15.87 (13.54-18.46)	11.14 (9.03-13.64)	12.33 (9.50-15.62)	28.28 (3.35-58.72)	-0.66 (-1.14 to -0.18)
Belize	0.84 (0.73-0.98)	5.52 (4.53-6.79)	2.06 (1.79-2.34)	4.84 (3.95-5.77)	145.76 (98.21-196.44)	-0.45 (-1.49-0.61)
Bermuda	6.42 (5.96-6.91)	65.60 (56.55-75.51)	6.13 (5.22-7.35)	23.43 (18.61-29.24)	-4.60 (-19.17-14.41)	-3.36 (-3.82 to -2.89)
Bolivarian Republic of Venezuela	171.91 (159.28-185.70)	12.02 (10.25-14.01)	473.24 (373.16-589.76)	10.04 (7.58-12.98)	175.29 (111.70-249.19)	-0.51 (-1.61-0.61)
Bosnia and Herzegovina	66.10 (48.10-90.73)	10.98 (7.42-15.68)	177.58 (127.20-235.51)	16.03 (10.69-22.64)	168.64 (67.43-330.04)	1.23 (1.01-1.46)
Brunei Darussalam	4.01 (3.16-4.95)	30.60 (17.61-49.51)	10.27 (8.53-12.29)	25.54 (17.87-35.87)	155.80 (91.23-249.47)	-0.6 (-1.01 to -0.19)
Burkina Faso	53.34 (28.41-111.71)	9.27 (4.39-19.90)	136.96 (66.64-266.75)	11.23 (5.12-23.14)	156.77 (79.70-259.47)	0.64 (0.45-0.83)
Canada	2059.75 (1891.69-2247.84)	36.87 (31.75-42.27)	1685.06 (1454.97-1836.25)	12.13 (10.02-14.09)	-18.19 (-27.61 to -9.54)	-3.53 (-4.06 to -3.00)
Central African Republic	19.57 (10.89-34.26)	14.27 (7.46-26.41)	28.09 (14.50-50.36)	11.39 (5.34-21.89)	43.53 (-0.41-93.71)	-0.72 (-0.84 to -0.61)
Commonwealth of Dominica	2.16 (1.65-2.75)	21.62 (13.92-31.58)	2.93 (2.36-3.63)	22.15 (14.96-32.02)	35.32 (-4.04-96.98)	0.05 (-0.09-0.19)
Commonwealth of the Bahamas	4.62 (4.17-5.22)	20.15 (16.92-23.90)	9.16 (7.60-10.94)	15.39 (12.28-18.94)	98.05 (57.95-143.54)	-0.86 (-1.7 to -0.01)
Cook Islands	0.31 (0.25-0.37)	18.46 (12.12-26.91)	0.67 (0.39-1.12)	15.42 (7.82-29.34)	118.00 (27.46-296.70)	-0.59 (-0.67 to -0.5)
Czech Republic	359.94 (334.23-388.33)	15.12 (13.52-16.90)	598.59 (520.47-679.33)	15.07 (12.69-17.50)	66.30 (42.51-90.74)	-0.09 (-0.79 to 0.62)

Democratic People's Republic of Korea	50.04 (36.25-65.61)	2.27 (1.37-3.51)	113.42 (85.17-146.86)	2.16 (1.32-3.36)	126.66 (64.30-215.11)	-0.15 (-0.22 to -0.09)
Democratic Republic of Sao Tome and Principe	0.93 (0.44-1.69)	9.84 (4.09-19.77)	1.76 (0.89-3.10)	12.28 (5.56-23.50)	89.97 (40.04-153.65)	0.70 (0.52-0.88)
Democratic Republic of the Congo	262.27 (130.69-467.39)	13.80 (6.21-26.29)	514.41 (256.15-917.19)	11.61 (5.34-22.00)	96.14 (39.87-177.49)	-0.54 (-0.70 to -0.39)
Democratic Republic of Timor-Leste	0.85 (0.61-1.29)	2.97 (1.83-4.80)	4.74 (3.11-7.42)	4.06 (2.34-6.65)	456.72 (267.56-719.13)	1.02 (0.94 to 1.11)
Democratic Socialist Republic of Sri Lanka	25.07 (20.78-30.63)	1.88 (1.33-2.55)	80.91 (55.34-113.94)	2.00 (1.25-3.03)	222.72 (101.43-411.94)	0.29 (-0.19-0.77)
Dominican Republic	40.18 (32.29-50.20)	7.96 (5.38-11.35)	140.96 (102.75-185.40)	8.74 (5.68-12.80)	250.83 (143.70-396.65)	0.35 (-0.11-0.81)
Eastern Republic of Uruguay	208.47 (194.01-225.50)	30.55 (25.93-35.40)	239.70 (217.60-258.80)	23.19 (19.50-26.98)	14.98 (2.64-27.07)	-0.90 (-1.2 to -0.6)
Federal Democratic Republic of Ethiopia	152.76 (88.06-278.25)	6.36 (3.47-12.25)	342.42 (175.70-581.98)	5.94 (2.97-10.28)	124.16 (44.43-262.76)	-0.21 (-0.33 to -0.08)
Federal Democratic Republic of Nepal	40.46 (22.58-72.89)	3.49 (1.73-6.62)	209.88 (143.29-327.23)	6.48 (3.78-10.90)	418.69 (244.27-692.09)	2.01 (1.93-2.09)
Federal Republic of Germany	4208.58 (3824.46-4518.61)	18.18 (15.47-21.02)	4483.27 (3892.54-4877.78)	11.85 (9.77-13.79)	6.53 (-4.58-18.68)	-1.39 (-1.96 to -0.82)
Federal Republic of Nigeria	835.81 (424.81-1539.94)	14.32 (7.24-26.22)	1384.28 (656.09-2494.85)	12.49 (5.90-22.18)	65.62 (13.68-127.82)	-0.43 (-0.61 to -0.26)
Federal Republic of Somalia	16.92 (8.80-34.72)	6.67 (3.05-14.57)	30.91 (14.45-73.78)	4.53 (1.91-11.10)	82.68 (19.88-171.72)	-1.22 (-1.36 to -1.08)
Federated States of Micronesia	1.29 (0.97-1.74)	21.92 (13.28-34.87)	1.50 (1.12-1.96)	18.17 (11.43-27.35)	16.82 (-14.35-65.61)	-0.61 (-0.67 to -0.56)
Federative Republic of Brazil	2209.81 (2100.72-2297.39)	16.85 (15.45-18.02)	8624.18 (7841.36-9139.66)	20.86 (18.48-22.67)	290.27 (265.30-311.34)	0.76 (0.58-0.94)

French Republic	3043.48 (2817.29-3219.33)	20.00 (16.90-23.17)	3030.11 (2601.80-3281.70)	10.32 (8.43-12.14)	-0.44 (-9.93-7.45)	-2.07 (-2.32 to -1.83)
Gabonese Republic	19.73 (12.10-28.36)	23.57 (13.16-38.17)	28.00 (17.42-40.69)	20.47 (11.45-33.59)	41.92 (-3.50-97.58)	-0.45 (-0.55 to -0.35)
Georgia	30.46 (25.74-35.94)	2.94 (2.36-3.60)	155.17 (131.33-180.39)	14.41 (11.81-17.37)	409.39 (288.66-546.65)	5.10 (3.88-6.33)
Grand Duchy of Luxembourg	22.64 (21.11-24.34)	23.70 (20.38-27.37)	24.67 (21.70-27.36)	12.58 (10.44-14.86)	8.96 (-3.46-22.18)	-2.07 (-2.57 to -1.57)
Greenland	0.88 (0.77-0.99)	21.98 (15.84-29.83)	0.98 (0.78-1.24)	10.03 (6.86-14.58)	11.43 (-13.57-45.82)	-2.39 (-3.00 to -1.76)
Grenada	3.51 (3.07-4.21)	25.80 (21.16-32.15)	4.36 (3.81-4.89)	25.02 (20.45-29.95)	24.23 (-0.50-49.81)	-0.01 (-0.73-0.72)
Guam	3.09 (2.61-3.66)	37.35 (27.17-49.64)	2.97 (2.48-3.51)	8.15 (5.86-11.16)	-4.07 (-25.38-23.20)	-4.76 (-5.48 to -4.03)
Hashemite Kingdom of Jordan	9.66 (7.30-12.78)	5.42 (3.69-7.79)	47.12 (35.87-61.22)	4.73 (3.20-6.79)	387.76 (212.75-652.04)	-0.4 (-0.68 to -0.12)
Hellenic Republic	535.90 (497.90-571.57)	20.57 (17.61-23.58)	1015.42 (905.85-1098.14)	21.41 (17.87-24.90)	89.48 (71.50-107.37)	0.11 (-0.33-0.54)
Hungary	405.54 (371.26-440.29)	16.37 (14.55-18.25)	528.83 (464.57-596.08)	14.89 (12.81-17.11)	30.40 (13.35-48.91)	-0.21 (-0.63 to 0.22)
Independent State of Papua New Guinea	15.79 (10.00-24.63)	7.70 (4.03-13.21)	45.90 (30.51-67.69)	8.07 (4.72-13.00)	190.67 (100.76-345.55)	0.15 (0.04-0.27)
Independent State of Samoa	1.99 (1.40-2.65)	17.95 (10.57-27.43)	2.90 (2.21-3.71)	14.51 (9.20-21.64)	45.96 (13.94-93.51)	-0.69 (-0.74 to -0.65)
Ireland	254.08 (236.82-272.13)	35.56 (30.65-40.70)	237.80 (197.68-264.77)	16.38 (13.06-19.35)	-6.41 (-19.28-5.24)	-2.39 (-2.76 to -2.02)
Islamic Republic of Afghanistan	4.34 (2.30-7.52)	0.42 (0.21-0.74)	16.06 (10.27-22.20)	1.42 (0.80-2.27)	270.06 (153.40-470.46)	4.04 (3.93-4.15)

Islamic Republic of Iran	46.29 (36.90-58.44)	1.42 (1.07-1.86)	303.80 (267.37-344.05)	2.68 (2.17-3.24)	556.24 (382.84-760.07)	1.98 (1.53-2.42)
Islamic Republic of Mauritania	21.11 (9.63-34.41)	14.67 (6.55-25.40)	38.76 (14.19-68.10)	12.92 (4.61-26.06)	83.61 (8.97-179.60)	-0.41 (-0.55 to -0.26)
Islamic Republic of Pakistan	366.52 (248.83-560.05)	4.53 (2.83-7.19)	1207.16 (870.02-1648.37)	7.96 (5.33-11.48)	229.35 (131.91-360.08)	1.84 (1.72-1.95)
Jamaica	30.97 (27.93-34.93)	9.75 (8.20-11.63)	52.47 (41.13-65.95)	9.70 (7.24-12.64)	69.45 (27.45-118.19)	-0.20 (-0.58-0.18)
Japan	4490.11 (4141.27-4673.24)	16.12 (14.69-17.07)	23012.10 (18379.46-25666.31)	27.10 (22.47-29.86)	412.51 (340.19-453.08)	1.64 (1.36-1.92)
Kingdom of Bahrain	0.66 (0.54-0.86)	3.32 (2.36-4.69)	3.50 (2.66-4.75)	3.73 (2.50-5.51)	427.68 (236.99-660.67)	0.34 (-0.40-1.09)
Kingdom of Belgium	785.31 (723.95-844.45)	28.28 (23.94-32.69)	586.53 (497.33-638.40)	12.44 (10.12-14.57)	-25.31 (-33.42 to -18.19)	-2.72 (-2.94 to -2.50)
Kingdom of Bhutan	1.21 (0.80-1.80)	4.25 (2.42-6.94)	7.47 (4.74-10.83)	8.18 (4.62-13.61)	520.00 (244.41-865.49)	2.15 (2.06-2.24)
Kingdom of Cambodia	16.74 (9.70-25.76)	3.00 (1.54-5.06)	63.91 (40.62-102.97)	4.07 (2.24-7.00)	281.68 (159.06-503.22)	1.01 (0.93-1.09)
Kingdom of Denmark	546.08 (499.17-587.55)	36.78 (31.47-42.47)	602.38 (532.75-657.82)	26.03 (21.67-30.20)	10.31 (-1.33-22.71)	-1.14 (-1.5 to -0.77)
Kingdom of Eswatini	4.40 (2.95-6.39)	13.04 (7.43-21.21)	7.67 (5.13-10.76)	11.36 (6.58-17.72)	74.40 (27.47-145.71)	-0.38 (-0.5 to -0.25)
Kingdom of Lesotho	10.18 (5.84-16.13)	8.90 (4.47-15.55)	14.04 (7.61-22.12)	10.31 (5.12-17.71)	37.89 (-6.66-100.80)	0.54 (0.28-0.79)
Kingdom of Morocco	13.09 (8.09-17.96)	0.62 (0.34-1.02)	113.96 (78.98-147.79)	2.21 (1.37-3.30)	770.46 (528.16-1111.21)	4.17 (3.92-4.42)
Kingdom of Norway	556.60 (522.60-582.11)	42.71 (39.19-45.47)	530.21 (455.96-570.32)	27.18 (23.60-29.68)	-4.74 (-12.01-0.90)	-1.43 (-1.75 to -1.10)

Kingdom of Saudi Arabia	2.82 (1.97-3.99)	0.38 (0.23-0.59)	19.97 (14.77-27.32)	0.98 (0.63-1.54)	609.28 (355.40-1013.16)	3.15 (2.87-3.42)
Kingdom of Spain	1303.87 (1218.51-1380.31)	13.58 (11.46-15.88)	2157.83 (1885.34-2334.57)	11.39 (9.30-13.48)	65.49 (50.68-78.77)	-0.63 (-0.97 to -0.30)
Kingdom of Sweden	1183.96 (1100.32-1251.95)	41.24 (36.01-46.28)	963.24 (825.60-1074.02)	21.89 (18.00-25.52)	-18.64 (-27.01 to -10.19)	-2.04 (-2.40 to -1.69)
Kingdom of Thailand	460.51 (345.55-623.04)	11.01 (7.11-16.45)	1960.65 (1488.79-2558.16)	10.66 (7.13-15.11)	325.75 (187.29-521.58)	-0.10 (-0.30-0.10)
Kingdom of the Netherlands	1486.37 (1366.78-1595.45)	41.76 (35.65-48.24)	1425.24 (1223.77-1563.79)	20.98 (17.34-24.38)	-4.11 (-13.23-5.06)	-2.26 (-2.61 to -1.91)
Kingdom of Tonga	1.16 (0.90-1.49)	15.36 (9.85-23.18)	2.04 (1.45-2.70)	16.13 (9.79-25.03)	76.52 (14.68-154.51)	0.16 (-0.05-0.37)
Kyrgyz Republic	6.14 (5.15-7.41)	1.31 (1.05-1.67)	27.00 (21.41-33.24)	3.85 (2.97-4.81)	339.47 (223.95-496.73)	4.57 (3.78-5.37)
Lao People's Democratic Republic	10.77 (6.92-16.77)	4.20 (2.39-6.82)	28.25 (20.83-39.14)	5.00 (3.20-7.74)	162.31 (76.39-306.69)	0.56 (0.51-0.61)
Lebanese Republic	44.75 (21.86-82.00)	13.57 (5.83-27.10)	120.56 (96.58-151.31)	11.17 (7.72-16.17)	169.42 (36.04-503.68)	-0.59 (-0.85 to -0.33)
Malaysia	237.29 (190.49-289.58)	18.33 (12.32-25.90)	887.69 (739.12-1072.42)	22.45 (15.53-32.13)	274.10 (177.67-408.97)	0.62 (0.30-0.95)
Mongolia	2.47 (1.71-3.39)	1.58 (1.05-2.27)	10.64 (8.03-13.59)	3.52 (2.57-4.72)	330.66 (185.66-571.76)	2.58 (2.21-2.95)
Montenegro	34.21 (26.56-44.94)	34.06 (24.78-46.66)	74.90 (56.67-98.81)	46.35 (32.23-65.50)	118.93 (54.07-224.20)	1.07 (0.71-1.44)
New Zealand	394.34 (364.97-419.78)	57.69 (49.71-65.56)	348.80 (304.53-378.24)	22.24 (18.58-25.61)	-11.55 (-20.52 to -3.55)	-3.3 (-4.02 to -2.57)
North Macedonia	28.96 (22.44-36.46)	10.38 (7.39-14.29)	73.81 (48.32-109.85)	14.40 (8.88-21.98)	154.88 (60.91-283.70)	1.15 (0.68-1.62)

Northern Mariana Islands	0.43 (0.34-0.53)	29.34 (19.30-42.30)	1.13 (0.90-1.42)	17.87 (10.81-27.92)	163.42 (90.13-276.29)	-1.85 (-2.59 to -1.1)
Palestine	4.02 (2.87-5.37)	3.17 (1.93-4.96)	13.28 (10.08-17.02)	3.96 (2.59-5.94)	229.88 (120.15-402.75)	0.64 (0.33-0.95)
People's Democratic Republic of Algeria	11.92 (8.39-16.37)	0.80 (0.48-1.25)	98.46 (73.09-130.06)	2.09 (1.28-3.22)	726.23 (462.58-1100.94)	3.2 (2.87-3.53)
People's Republic of Bangladesh	286.59 (169.36-505.58)	4.41 (2.25-8.55)	1306.39 (842.36-2159.40)	6.62 (3.76-11.68)	355.84 (206.71-624.79)	1.36 (0.94-1.78)
People's Republic of China	1647.66 (1307.92-2088.43)	1.46 (1.15-1.84)	6743.41 (5284.35-8574.60)	1.97 (1.52-2.51)	309.27 (175.62-488.90)	0.99 (0.89-1.08)
Plurinational State of Bolivia	28.26 (18.24-41.05)	6.46 (3.68-10.22)	94.28 (70.63-129.27)	7.20 (4.47-11.04)	233.63 (127.08-415.36)	0.36 (0.26-0.46)
Portuguese Republic	218.23 (203.98-232.38)	9.30 (7.98-10.69)	385.66 (342.64-416.62)	8.09 (6.74-9.50)	76.72 (59.52-93.90)	-0.41 (-1.38 to 0.57)
Principality of Andorra	3.43 (2.17-5.15)	38.54 (21.29-65.86)	6.17 (3.95-9.29)	22.20 (12.41-37.06)	79.83 (15.36-179.40)	-1.93 (-2.57 to -1.28)
Principality of Monaco	4.34 (3.23-5.41)	31.02 (20.32-44.87)	5.70 (4.30-7.63)	28.39 (18.20-42.43)	31.36 (-7.44-95.50)	-0.3 (-0.45 to -0.15)
Puerto Rico	75.71 (70.18-80.61)	12.89 (11.06-14.87)	74.19 (60.68-86.92)	5.03 (3.97-6.22)	-2.01 (-18.51-15.61)	-2.94 (-3.85 to -2.02)
Republic of Albania	14.49 (11.89-17.18)	4.91 (3.62-6.49)	43.67 (30.80-59.84)	5.81 (3.43-8.97)	201.48 (100.30-345.70)	0.41 (-0.01-0.83)
Republic of Angola	65.71 (36.09-103.89)	14.11 (7.34-23.65)	216.92 (128.76-327.19)	15.58 (8.43-26.14)	230.12 (114.78-403.57)	0.32 (0.13-0.51)
Republic of Armenia	96.25 (78.11-118.13)	22.99 (18.29-29.06)	367.49 (305.24-432.52)	48.85 (40.01-58.53)	281.80 (186.85-414.19)	2.45 (1.74-3.16)
Republic of Austria	373.58 (349.90-395.46)	17.39 (14.98-19.82)	350.26 (307.20-380.57)	9.94 (8.23-11.54)	-6.24 (-14.27-1.77)	-1.65 (-1.94 to -1.36)

Republic of Azerbaijan	26.87 (19.97-35.75)	3.58 (2.41-5.16)	107.54 (70.39-167.80)	7.02 (4.19-11.07)	300.30 (138.19-530.88)	2.24 (1.73- 2.74)
Republic of Belarus	277.78 (239.88-338.05)	12.64 (10.27-15.74)	515.10 (422.92-615.33)	18.05 (14.22-22.49)	85.44 (44.80-137.37)	1.22 (0.48-1.97)
Republic of Benin	25.04 (11.82-44.61)	8.60 (3.76-16.61)	57.77 (23.11-105.41)	8.64 (3.30-17.24)	130.75 (65.85-214.98)	0.02 (-0.07-0.12)
Republic of Botswana	8.94 (5.73-14.01)	13.66 (7.70-23.06)	20.54 (12.77-27.79)	11.90 (6.72-18.77)	129.83 (39.19-299.01)	-0.42 (-0.72 to -0.12)
Republic of Bulgaria	178.91 (158.17-198.64)	9.71 (8.46-11.05)	325.79 (265.91-402.54)	12.95 (10.27-16.20)	82.10 (41.99-135.07)	0.95 (-0.07-1.97)
Republic of Burundi	41.42 (23.00-66.36)	12.70 (6.24-22.98)	44.66 (19.18-83.40)	7.70 (3.13-15.45)	7.83 (-47.51-74.62)	-1.6 (-1.69 to -1.51)
Republic of Cabo Verde	4.11 (1.62-8.59)	9.88 (3.48-21.46)	8.92 (4.38-15.83)	13.22 (5.93-25.23)	117.09 (49.86-239.16)	0.88 (0.39-1.38)
Republic of Cameroon	74.64 (47.76-130.98)	13.12 (7.41-24.18)	196.27 (118.20-339.70)	12.46 (6.86-21.77)	162.96 (83.81-286.04)	-0.17 (-0.3 to -0.04)
Republic of Chad	31.36 (14.96-65.10)	7.56 (3.24-16.70)	59.68 (25.68-118.10)	8.15 (3.20-17.51)	90.29 (13.54-188.27)	0.26 (0.17-0.35)
Republic of Chile	223.03 (209.73-235.07)	14.36 (12.44-16.45)	511.05 (464.37-549.49)	11.42 (9.58-13.26)	129.14 (109.08-148.83)	-0.75 (-1.49 to -0.01)
Republic of Colombia	468.99 (441.59-496.99)	18.14 (15.66-20.76)	1465.86 (1221.25-1729.17)	15.72 (12.43-19.38)	212.56 (161.10-273.60)	-0.35 (-1.51 to 0.82)
Republic of Costa Rica	34.41 (31.21-38.24)	12.60 (10.62-14.84)	117.35 (101.81-132.25)	12.62 (10.29-15.01)	241.00 (188.93-295.04)	0.08 (-0.3-0.46)
Republic of Croatia	135.49 (117.20-157.04)	14.34 (12.32-16.68)	303.18 (258.38-349.08)	18.21 (15.11-21.35)	123.76 (77.70-177.57)	0.62 (-0.28-1.53)
Republic of Cuba	537.99 (492.96-581.51)	31.94 (27.59-36.64)	740.03 (628.29-834.80)	20.98 (17.31-24.98)	37.55 (18.68-61.70)	-1.42 (-1.84 to -1)

Republic of Cyprus	47.18 (36.38-60.80)	44.37 (27.47-66.23)	77.26 (58.05-97.75)	22.33 (14.81-32.47)	63.75 (11.49-141.16)	-2.16 (-2.66 to -1.65)
Republic of Côte d'Ivoire	61.48 (29.33-110.56)	13.75 (6.05-26.30)	174.16 (78.43-313.20)	13.01 (5.42-25.50)	183.25 (99.92-295.83)	-0.18 (-0.3 to -0.07)
Republic of Djibouti	1.75 (0.90-2.76)	11.92 (5.70-20.74)	7.40 (3.24-12.08)	10.37 (4.22-18.66)	323.38 (170.66-547.83)	-0.43 (-0.55 to -0.32)
Republic of Ecuador	55.32 (50.88-59.81)	7.18 (6.08-8.36)	178.74 (143.45-223.24)	6.87 (5.26-8.95)	223.10 (155.22-314.24)	-0.23 (-1.39 to 0.95)
Republic of El Salvador	12.51 (10.38-15.00)	2.67 (1.92-3.63)	29.12 (22.73-37.05)	2.69 (1.86-3.81)	132.75 (66.91-212.34)	0.01 (-0.83-0.86)
Republic of Equatorial Guinea	3.61 (2.06-5.96)	14.31 (7.56-25.24)	11.21 (5.56-19.01)	18.16 (8.41-32.94)	210.25 (62.41-455.16)	0.8 (0.63-0.97)
Republic of Estonia	50.04 (45.76-54.98)	14.42 (12.41-16.60)	92.17 (80.37-104.23)	17.71 (14.71-20.83)	84.19 (57.56-116.96)	0.78 (-0.1-1.68)
Republic of Fiji	7.24 (5.85-8.88)	18.11 (12.53-25.22)	17.58 (13.17-22.63)	19.23 (13.12-27.11)	142.70 (75.44-247.50)	0.19 (-0.15-0.54)
Republic of Finland	536.12 (496.57-572.05)	42.42 (36.19-48.75)	501.40 (433.34-548.05)	19.70 (16.14-23.01)	-6.48 (-16.22-3.32)	-2.45 (-2.86 to -2.04)
Republic of Ghana	130.31 (63.42-218.21)	16.56 (7.42-29.85)	327.05 (150.03-544.27)	15.35 (6.68-28.09)	150.97 (68.61-257.41)	-0.23 (-0.4 to -0.06)
Republic of Guatemala	13.63 (12.40-15.12)	3.11 (2.61-3.70)	36.92 (31.47-43.25)	2.20 (1.76-2.67)	170.89 (122.89-229.14)	-1.02 (-2.11 to 0.08)
Republic of Guinea	44.41 (21.10-85.29)	9.18 (4.06-19.17)	78.67 (28.91-153.28)	10.19 (3.50-21.56)	77.15 (2.58-159.48)	0.33 (0.26-0.4)
Republic of Guinea-Bissau	6.79 (3.63-12.20)	13.14 (6.46-25.18)	9.91 (5.23-17.78)	11.85 (5.74-22.53)	45.92 (4.83-110.07)	-0.33 (-0.48 to -0.19)
Republic of Guyana	5.87 (5.25-6.47)	10.88 (9.18-12.68)	17.26 (13.33-21.79)	18.46 (13.66-24.10)	194.14 (120.11-274.15)	1.86 (0.93-2.8)

Republic of Haiti	53.24 (34.84-80.98)	13.05 (7.58-21.20)	113.47 (70.80-176.07)	12.64 (7.10-21.29)	113.12 (53.60-191.94)	-0.09 (-0.2 to 0.01)
Republic of Honduras	12.10 (8.91-16.67)	4.25 (2.66-6.55)	58.74 (41.75-79.24)	6.51 (3.96-9.98)	385.36 (239.09-653.51)	1.43 (1.15-1.72)
Republic of Iceland	12.37 (11.08-13.27)	23.99 (20.27-27.53)	16.13 (13.53-18.07)	14.50 (11.59-17.12)	30.33 (15.04-46.66)	-1.63 (-1.87 to -1.39)
Republic of India	2145.30 (1276.94-3504.88)	3.56 (2.11-5.79)	11234.41 (8054.26-16394.59)	6.45 (4.57-9.40)	423.67 (285.38-642.50)	1.99 (1.32-2.66)
Republic of Indonesia	405.43 (265.40-551.13)	3.41 (2.09-4.87)	1566.25 (1074.87-2100.08)	5.63 (3.69-7.83)	286.32 (149.21-453.47)	1.64 (1.59-1.7)
Republic of Iraq	15.60 (11.22-20.88)	1.28 (0.80-1.94)	60.08 (43.44-78.45)	1.79 (1.15-2.62)	285.22 (144.63-526.29)	1.11 (0.87-1.35)
Republic of Italy	2642.50 (2472.76-2746.40)	16.75 (15.26-17.86)	3507.33 (3039.69-3789.43)	11.81 (10.25-12.89)	32.73 (21.80-40.76)	-1.14 (-1.48 to -0.79)
Republic of Kazakhstan	135.63 (108.66-173.29)	6.95 (5.46-9.02)	311.79 (249.89-380.29)	11.31 (8.92-13.89)	129.88 (62.94-223.81)	1.6 (1.01-2.19)
Republic of Kenya	84.61 (53.94-123.46)	7.78 (4.91-11.44)	269.52 (158.48-376.16)	9.54 (5.51-13.58)	218.54 (133.06-307.94)	0.68 (0.6-0.76)
Republic of Kiribati	0.09 (0.08-0.11)	2.04 (1.34-3.00)	0.20 (0.14-0.26)	2.26 (1.41-3.41)	109.75 (41.22-191.91)	0.32 (0.29-0.35)
Republic of Korea	354.28 (237.79-499.76)	10.00 (6.13-15.16)	1669.97 (1347.70-1975.45)	10.56 (7.44-14.01)	371.37 (191.21-679.10)	0.17 (-0.03-0.37)
Republic of Latvia	69.35 (63.67-75.64)	11.29 (9.77-13.00)	114.93 (98.44-129.98)	15.45 (12.78-18.40)	65.71 (42.25-93.20)	1.3 (0.55-2.05)
Republic of Liberia	19.41 (10.11-35.40)	11.89 (5.65-23.15)	26.62 (11.57-50.74)	10.53 (4.26-22.07)	37.12 (-8.74-89.89)	-0.4 (-0.67 to -0.13)
Republic of Lithuania	81.59 (74.70-89.64)	10.57 (9.15-12.14)	170.66 (148.76-193.18)	15.83 (13.15-18.76)	109.16 (80.60-141.86)	1.22 (0.99-1.45)

Republic of Madagascar	108.81 (54.32-190.77)	15.79 (7.03-29.48)	165.19 (80.03-282.63)	12.95 (5.74-23.43)	51.81 (8.63-114.13)	-0.67 (-0.81 to -0.53)
Republic of Malawi	38.44 (18.48-73.06)	7.91 (3.42-16.04)	91.85 (45.00-159.35)	9.61 (4.40-17.70)	138.93 (71.60-237.16)	0.64 (0.53-0.75)
Republic of Maldives	0.35 (0.24-0.49)	3.53 (2.13-5.71)	1.41 (0.73-2.26)	3.24 (1.60-5.73)	304.78 (140.94-552.90)	-0.34 (-0.54 to -0.15)
Republic of Mali	33.25 (16.62-67.20)	6.66 (3.05-14.09)	71.10 (29.75-140.83)	6.34 (2.52-13.41)	113.84 (22.86-256.66)	-0.14 (-0.32 to 0.05)
Republic of Malta	10.31 (9.54-11.15)	14.51 (12.26-16.92)	13.52 (11.84-15.18)	7.18 (5.83-8.62)	31.13 (14.42-49.34)	-2.45 (-3.18 to -1.72)
Republic of Mauritius	7.77 (7.26-8.27)	8.10 (6.94-9.31)	13.52 (12.33-14.40)	4.69 (3.93-5.47)	73.95 (58.10-88.67)	-1.92 (-3.66 to -0.13)
Republic of Moldova	31.51 (28.27-34.86)	4.73 (4.07-5.47)	79.11 (69.79-89.16)	7.54 (6.36-8.88)	151.05 (113.05-200.11)	1.61 (0.24-3.00)
Republic of Mozambique	74.59 (32.79-146.91)	9.95 (4.05-20.50)	173.82 (72.90-335.52)	12.48 (4.96-25.21)	133.02 (60.02-215.35)	0.75 (0.65-0.85)
Republic of Namibia	10.63 (6.91-16.19)	13.73 (7.93-22.59)	22.48 (16.45-30.67)	13.29 (8.21-20.75)	111.56 (52.02-208.30)	-0.09 (-0.16 to -0.02)
Republic of Nauru	0.14 (0.10-0.18)	25.55 (15.95-39.48)	0.19 (0.12-0.27)	32.25 (14.32-58.44)	38.56 (-8.91-101.03)	0.75 (0.7-0.8)
Republic of Nicaragua	3.93 (3.30-4.65)	1.77 (1.25-2.41)	13.51 (10.60-16.85)	1.82 (1.27-2.57)	243.77 (155.45-347.88)	0.13 (-0.03-0.29)
Republic of Niue	0.08 (0.06-0.10)	19.55 (12.69-28.75)	0.06 (0.05-0.07)	17.08 (11.11-24.92)	-27.10 (-45.32 to -4.87)	-0.44 (-0.51 to -0.37)
Republic of Palau	0.21 (0.16-0.27)	17.35 (11.16-25.77)	0.39 (0.30-0.52)	15.03 (9.58-23.01)	87.96 (34.03-169.15)	-0.5 (-0.65 to -0.35)
Republic of Panama	28.06 (25.40-30.52)	12.09 (10.07-14.17)	70.01 (53.54-84.86)	9.31 (6.89-11.74)	149.46 (93.19-203.09)	-0.75 (-1.34 to -0.15)

Republic of Paraguay	36.09 (29.17-44.81)	10.48 (7.52-14.38)	139.90 (106.15-180.26)	15.16 (10.36-21.19)	287.65 (179.34-451.02)	1.17 (0.83-1.52)
Republic of Peru	72.48 (56.63-93.73)	4.05 (2.69-5.94)	187.02 (137.05-248.66)	3.39 (2.16-5.08)	158.02 (75.08-278.97)	-0.4 (-1.09 to 0.29)
Republic of Poland	1743.51 (1674.71-1796.37)	23.83 (22.52-24.89)	2422.15 (2169.39-2662.28)	18.41 (16.21-20.45)	38.92 (26.66-52.95)	-1.04 (-1.46 to -0.62)
Republic of Rwanda	51.63 (30.37-81.68)	14.33 (7.57-24.73)	76.75 (45.86-127.71)	9.83 (5.38-17.77)	48.64 (-9.06-144.25)	-1.17 (-1.41 to -0.93)
Republic of San Marino	1.10 (0.88-1.35)	17.18 (11.42-25.26)	1.42 (0.92-2.20)	9.33 (5.04-15.72)	28.89 (-17.92-100.66)	-2.33 (-2.63 to -2.04)
Republic of Senegal	51.49 (24.23-95.71)	11.49 (4.93-23.35)	121.23 (50.56-223.81)	11.48 (4.52-22.83)	135.43 (67.74-215.11)	-0.01 (-0.29 to 0.28)
Republic of Serbia	349.83 (290.10-418.50)	22.46 (16.32-30.05)	652.48 (518.59-820.94)	21.76 (15.19-30.06)	86.51 (40.83-147.56)	-0.10 (-0.45 to 0.24)
Republic of Seychelles	0.55 (0.44-0.66)	5.73 (3.96-8.15)	0.95 (0.65-1.34)	5.74 (3.35-9.26)	74.47 (25.21-141.53)	0.08 (-0.18-0.35)
Republic of Sierra Leone	35.09 (16.78-65.53)	11.60 (5.12-23.18)	51.11 (22.37-99.60)	10.11 (4.02-20.89)	45.67 (1.55-111.72)	-0.44 (-0.51 to -0.38)
Republic of Singapore	45.62 (42.92-48.06)	14.40 (12.40-16.50)	164.89 (145.59-178.52)	11.82 (9.83-13.72)	261.43 (222.98-296.89)	-0.65 (-1.28 to -0.01)
Republic of Slovenia	57.61 (52.92-61.99)	13.67 (11.76-15.69)	100.93 (84.85-121.43)	11.81 (9.47-14.71)	75.21 (47.02-113.09)	-0.55 (-1.19 to 0.1)
Republic of South Africa	461.88 (347.83-579.91)	15.99 (11.57-20.83)	762.90 (676.06-849.75)	11.81 (9.93-13.95)	65.17 (33.77-120.72)	-0.97 (-1.31 to -0.63)
Republic of South Sudan	38.20 (15.20-70.69)	10.38 (3.97-20.72)	33.91 (14.78-66.18)	7.33 (2.93-14.63)	-11.25 (-38.46-29.53)	-1.12 (-1.22 to -1.02)
Republic of Sudan	6.35 (3.49-14.41)	0.47 (0.23-1.13)	43.51 (28.03-63.20)	1.63 (0.94-2.59)	585.73 (233.91-1151.65)	4.10 (4.01-4.19)

Republic of Suriname	4.93 (4.12-5.89)	12.99 (9.08-18.20)	11.01 (7.62-15.34)	10.95 (6.72-16.87)	123.18 (47.06-216.73)	-0.46 (-1.04 to 0.13)
Republic of Tajikistan	5.85 (3.94-7.77)	1.41 (0.83-2.24)	9.64 (6.96-13.31)	1.28 (0.83-1.93)	64.83 (12.00-149.57)	-0.41 (-1.03 to 0.21)
Republic of the Congo	29.50 (18.58-43.71)	21.10 (11.95-33.97)	58.05 (34.50-88.25)	18.15 (10.02-30.50)	96.79 (41.87-170.56)	-0.47 (-0.62 to -0.32)
Republic of the Gambia	5.42 (2.32-10.48)	11.96 (4.68-24.60)	17.26 (7.97-30.23)	13.06 (5.44-25.16)	218.23 (127.67-350.79)	0.28 (0.02-0.53)
Republic of the Marshall Islands	0.30 (0.20-0.44)	16.56 (9.58-27.17)	0.54 (0.37-0.77)	14.91 (8.88-22.99)	78.60 (32.34-146.10)	-0.35 (-0.44 to -0.25)
Republic of the Niger	23.48 (9.92-49.10)	7.40 (2.97-16.81)	64.15 (23.76-148.57)	6.44 (2.28-15.65)	173.23 (73.01-282.77)	-0.44 (-0.63 to -0.26)
Republic of the Philippines	232.39 (194.48-274.38)	6.54 (5.20-7.98)	779.27 (639.70-914.90)	7.12 (5.75-8.50)	235.33 (171.85-329.86)	0.25 (-0.08-0.59)
Republic of the Union of Myanmar	125.06 (73.19-186.46)	4.25 (2.33-6.94)	340.17 (255.68-447.27)	5.16 (3.29-7.68)	172.00 (82.05-321.54)	0.65 (0.61-0.69)
Republic of Trinidad and Tobago	37.51 (34.55-41.13)	30.15 (25.35-35.26)	68.94 (53.32-87.30)	21.36 (15.95-27.83)	83.82 (39.25-135.39)	-0.93 (-1.43 to -0.42)
Republic of Tunisia	5.43 (3.74-7.44)	0.75 (0.45-1.15)	46.30 (30.02-67.54)	2.26 (1.29-3.73)	751.90 (431.35-1279.48)	3.59 (3.37-3.82)
Republic of Turkey	514.45 (371.09-728.65)	9.75 (6.04-14.99)	1727.26 (1344.37-2173.25)	11.27 (7.85-15.91)	235.75 (115.23-402.10)	0.48 (0.21-0.74)
Republic of Uganda	71.87 (33.77-138.74)	8.33 (3.68-16.83)	157.70 (77.90-262.87)	8.50 (3.97-15.59)	119.43 (47.18-231.21)	0.07 (-0.04-0.18)
Republic of Uzbekistan	18.58 (13.45-27.06)	1.03 (0.74-1.54)	190.77 (152.48-235.80)	5.48 (4.33-6.85)	926.56 (560.54-1421.79)	5.33 (4.53-6.13)
Republic of Vanuatu	0.80 (0.55-1.16)	11.40 (6.39-18.82)	2.22 (1.63-2.97)	11.30 (6.93-17.66)	179.23 (108.90-276.23)	-0.03 (-0.15 to 0.08)

Republic of Yemen	3.27 (1.73-5.78)	0.48 (0.24-0.87)	30.87 (18.26-48.31)	1.62 (0.86-2.74)	845.18 (510.92-1454.63)	4.01 (3.7-4.32)
Republic of Zambia	42.30 (27.06-63.93)	11.99 (6.56-20.58)	150.11 (67.09-264.65)	17.59 (7.57-32.32)	254.87 (94.19-520.72)	1.26 (1.07-1.46)
Republic of Zimbabwe	90.47 (72.29-109.81)	18.43 (12.45-26.77)	167.77 (127.14-222.80)	20.80 (13.22-30.26)	85.44 (29.96-165.41)	0.45 (0.22-0.68)
Romania	294.66 (270.01-321.32)	6.75 (5.88-7.73)	623.99 (542.78-717.85)	9.42 (7.84-11.21)	111.76 (80.32-147.98)	1.15 (0.41-1.9)
Russian Federation	3887.51 (3750.36-3980.56)	13.27 (12.64-13.74)	9308.95 (8507.03-10081.25)	22.07 (19.73-24.23)	139.46 (120.49-157.82)	2.13 (0.97-3.31)
Saint Kitts and Nevis	1.35 (1.22-1.50)	20.26 (17.30-23.52)	1.28 (1.05-1.58)	13.78 (10.89-17.52)	-5.29 (-23.45-16.98)	-1.21 (-1.91 to -0.5)
Saint Lucia	7.31 (6.64-8.17)	57.46 (48.30-68.19)	10.75 (9.05-12.53)	27.67 (22.02-34.00)	47.17 (21.45-76.36)	-2.38 (-3.08 to -1.68)
Saint Vincent and the Grenadines	1.65 (1.52-1.81)	14.11 (12.02-16.42)	2.59 (2.29-2.92)	11.51 (9.57-13.69)	56.86 (35.81-84.40)	-0.64 (-1.14 to -0.13)
Slovak Republic	116.44 (99.92-137.37)	11.43 (8.71-14.96)	197.13 (155.30-249.16)	11.77 (8.23-16.37)	69.30 (21.95-133.11)	0.16 (-0.2-0.51)
Socialist Republic of Viet Nam	213.20 (151.93-290.57)	3.64 (2.23-5.68)	803.77 (588.02-1063.49)	5.87 (3.64-9.03)	277.00 (158.00-482.55)	1.56 (1.49-1.62)
Solomon Islands	1.31 (0.85-2.12)	9.49 (5.29-16.64)	3.78 (2.57-5.51)	10.44 (5.87-17.44)	189.03 (102.26-297.64)	0.29 (0.17-0.41)
State of Eritrea	11.25 (5.86-20.45)	9.07 (4.24-17.69)	30.38 (13.59-57.83)	9.97 (4.07-19.94)	170.14 (54.13-370.87)	0.29 (0.16-0.43)
State of Israel	105.31 (96.42-113.41)	12.93 (10.99-14.94)	165.06 (144.24-180.16)	7.22 (5.86-8.48)	56.73 (39.49-76.48)	-1.87 (-2.14 to -1.6)
State of Kuwait	3.49 (3.12-3.88)	4.61 (3.83-5.46)	15.62 (12.84-19.29)	4.38 (3.32-5.58)	347.23 (256.83-464.84)	0.46 (-2.66-3.68)

State of Libya	1.53 (1.09-2.22)	0.55 (0.33-0.92)	11.35 (7.16-17.39)	1.60 (0.89-2.64)	640.20 (378.57-1071.41)	3.54 (3.18-3.89)
State of Qatar	0.76 (0.60-0.97)	7.86 (5.23-11.42)	4.33 (2.41-7.61)	5.04 (2.71-9.16)	471.23 (174.37-1052.25)	-1.39 (-2.26 to -0.5)
Sultanate of Oman	0.76 (0.48-1.21)	0.84 (0.46-1.46)	7.00 (4.19-11.29)	2.82 (1.44-5.21)	819.70 (276.69-1985.87)	4.03 (3.65-4.41)
Swiss Confederation	512.24 (459.25-555.95)	26.72 (22.44-31.23)	474.90 (399.47-527.06)	12.84 (10.33-15.04)	-7.29 (-17.80-4.48)	-2.25 (-2.44 to -2.07)
Syrian Arab Republic	12.63 (8.72-17.12)	1.68 (1.05-2.56)	39.24 (28.62-52.68)	2.06 (1.31-3.09)	210.73 (97.00-395.26)	0.61 (0.38 to 0.84)
Taiwan (Province of China)	152.01 (141.55-162.38)	6.49 (5.55-7.50)	842.65 (743.85-918.26)	11.28 (9.42-13.23)	454.33 (395.52-513.09)	1.88 (0.86-2.92)
Togolese Republic	19.79 (9.59-36.41)	12.70 (5.57-25.19)	56.51 (23.87-101.68)	12.28 (4.85-24.62)	185.50 (101.08-278.47)	-0.11 (-0.23 to 0)
Tokelau	0.04 (0.03-0.05)	17.88 (11.05-27.06)	0.05 (0.03-0.07)	17.90 (9.33-30.33)	24.86 (-24.16-89.16)	-0.02 (-0.14 to 0.1)
Turkmenistan	14.20 (11.63-17.40)	5.03 (4.02-6.29)	54.04 (40.58-77.00)	9.12 (6.76-13.02)	280.68 (152.22-456.22)	2.06 (1.58-2.54)
Tuvalu	0.13 (0.08-0.18)	16.47 (9.53-26.78)	0.21 (0.17-0.27)	15.58 (10.29-22.68)	68.44 (23.67-144.01)	-0.18 (-0.22 to -0.13)
Ukraine	1242.61 (1121.17-1385.91)	10.19 (8.64-12.06)	1561.40 (1179.08-2013.21)	11.31 (8.24-15.05)	25.65 (-10.10-67.48)	0.38 (-0.36-1.13)
Union of the Comoros	3.17 (1.21-5.82)	12.41 (4.82-23.93)	6.96 (2.63-12.81)	10.47 (3.68-21.00)	119.67 (39.79-225.98)	-0.56 (-0.73 to -0.39)
United Arab Emirates	2.91 (1.78-4.36)	6.44 (3.55-10.51)	23.47 (18.55-30.67)	7.88 (5.19-11.43)	706.60 (420.34-1317.47)	0.52 (-1.86-2.97)
United Kingdom of Great Britain and Northern Ireland	9338.55 (8800.54-9610.76)	55.45 (52.03-57.56)	5925.16 (5226.43-6287.04)	22.74 (20.14-24.24)	-36.55 (-40.94 to -34.25)	-2.88 (-3.12 to -2.63)

United Mexican States	242.40 (233.76-249.98)	4.21 (3.95-4.47)	643.82 (568.73-726.14)	3.27 (2.84-3.73)	165.60 (136.84-197.95)	-0.91 (-1.63 to -0.19)
United Republic of Tanzania	173.38 (103.26-287.67)	12.03 (6.45-21.66)	454.19 (220.76-788.46)	13.33 (6.00-25.41)	161.96 (54.49-354.13)	0.35 (0.25-0.45)
United States of America	16478.81 (15192.02-17190.81)	28.52 (26.00-30.06)	11017.55 (9736.24-11744.76)	10.26 (9.03-11.04)	-33.14 (-36.27 to -30.67)	-3.27 (-3.64 to -2.91)
United States Virgin Islands	2.03 (1.66-2.43)	18.07 (12.45-25.12)	3.47 (2.63-4.39)	10.96 (7.13-16.29)	71.08 (22.77-138.18)	-1.67 (-2.06 to -1.27)

AA, aortic aneurysm; AAPC, average annual percentage change; ASMR, age-standardized mortality rate; UI, uncertainty intervals; CI, confidence interval.

Table 5. DALYs of AA among individuals aged 55 and older in 204 countries and territories, with the associated AAPCs from 1990 to 2021.

	**1990**		**2021**		**1990-2021**	
**Characteristics**	**Cases (95% UI)**	**ASDR per 100,000 (95% UI)**	**Cases (95% UI)**	**ASDR per 100,000 (95% UI)**	**Cases change (95% CI)**	**AACP (95% CI)**
American Samoa	10.45 (8.76-12.40)	344.22 (238.35-485.39)	17.80 (14.74-21.74)	241.59 (166.96-338.04)	70.27 (32.37-117.18)	-1.04 (-1.61 to -0.47)
Antigua and Barbuda	27.43 (24.85-30.39)	290.00 (245.73-341.38)	32.84 (29.54-37.63)	189.52 (158.60-229.46)	19.73 (5.71-34.03)	-1.43 (-2.72 to -0.13)
Arab Republic of Egypt	1367.56 (947.42-2042.24)	34.15 (21.05-54.47)	4146.34 (3210.85-5308.62)	42.86 (29.59-60.96)	203.19 (87.45-365.83)	0.77 (0.59-0.95)
Argentine Republic	29230.99 (26692.75-32351.42)	527.81 (447.50-617.06)	25922.38 (23652.26-28302.66)	268.63 (226.43-315.03)	-11.32 (-23.49-1.15)	-2.07 (-2.35 to -1.79)
Australia	25199.04 (23453.32-26987.03)	737.27 (638.74-841.35)	16997.35 (15006.24-18361.06)	204.35 (171.35-235.99)	-32.55 (-39.23 to -26.58)	-4.11 (-4.66 to -3.56)

Barbados	143.79 (131.49-157.30)	270.86 (231.21-316.05)	187.31 (149.00-232.70)	204.65 (154.74-262.30)	30.27 (3.56-63.84)	-0.75 (-1.23 to -0.26)
Belize	15.10 (13.22-17.60)	98.77 (81.39-121.55)	38.45 (33.24-43.71)	84.75 (69.08-101.43)	154.63 (102.81-210.46)	-0.56 (-1.57-0.47)
Bermuda	115.94 (108.11-125.10)	1132.83 (977.40-1308.83)	95.27 (81.17-115.18)	379.70 (301.32-477.17)	-17.83 (-30.64 to -1.60)	-3.52 (-4.58 to -2.45)
Bolivarian Republic of Venezuela	3463.52 (3220.24-3753.07)	230.83 (197.80-268.74)	8930.58 (6918.05-11290.65)	180.42 (134.57-235.74)	157.85 (94.91-232.36)	-0.75 (-1.83 to 0.33)
Bosnia and Herzegovina	1418.42 (1027.91-1957.23)	212.74 (143.50-304.56)	3413.75 (2457.37-4629.38)	307.45 (204.68-436.77)	140.67 (47.36-297.76)	1.18 (0.90-1.46)
Brunei Darussalam	73.17 (53.98-94.16)	517.18 (299.62-818.76)	205.43 (171.26-245.69)	427.48 (301.48-593.52)	180.74 (103.19-301.39)	-0.63 (-0.96 to -0.30)
Burkina Faso	1079.30 (570.67-2252.04)	165.65 (78.47-355.33)	2641.05 (1251.25-5244.76)	196.35 (88.53-404.12)	144.70 (72.52-244.99)	0.55 (0.40-0.71)
Canada	35968.44 (33229.80-39109.48)	636.83 (551.38-732.01)	25658.05 (22824.13-27896.86)	193.28 (161.96-224.88)	-28.67 (-36.38 to -20.56)	-3.86 (-4.51 to -3.20)
Central African Republic	450.79 (255.10-792.20)	275.07 (143.01-509.40)	653.59 (344.08-1194.49)	218.33 (102.58-421.95)	44.99 (0.98-96.34)	-0.75 (-0.87 to -0.63)
Commonwealth of Dominica	37.36 (27.90-47.63)	363.95 (234.30-531.80)	52.86 (42.31-67.35)	378.30 (257.30-543.19)	41.48 (-2.72-110.44)	0.08 (-0.05-0.22)
Commonwealth of the Bahamas	90.72 (81.87-102.92)	379.53 (319.61-449.89)	175.87 (145.18-211.99)	272.39 (215.61-337.56)	93.87 (52.76-141.23)	-1.01 (-2.01 to -0.01)
Cook Islands	6.04 (4.80-7.36)	318.76 (209.93-460.86)	11.70 (6.65-20.16)	259.35 (130.16-499.34)	93.93 (12.19-258.69)	-0.67 (-0.75 to -0.59)
Czech Republic	7255.74 (6735.30-7799.95)	301.84 (270.43-337.58)	10659.60 (9223.44-12082.91)	279.18 (234.01-327.66)	46.91 (24.73-70.51)	-0.27 (-0.89-0.36)
Democratic People's Republic of Korea	1130.02 (804.74-1515.39)	44.90 (27.38-68.85)	2459.25 (1816.33-3197.03)	44.38 (27.59-67.80)	117.63 (53.02-213.23)	-0.04 (-0.11-0.03)

Democratic Republic of Sao Tome and Principe	17.00 (8.23-31.07)	165.96 (71.16-327.27)	34.66 (17.67-61.57)	216.99 (99.51-412.65)	103.86 (48.74-175.11)	0.86 (0.73-1.00)
Democratic Republic of the Congo	5855.67 (2895.29-10583.79)	257.82 (117.42-489.48)	11180.27 (5526.22-19997.86)	217.29 (99.91-410.43)	90.93 (34.94-168.23)	-0.54 (-0.71 to -0.37)
Democratic Republic of Timor-Leste	17.02 (12.20-25.62)	50.70 (31.76-81.91)	87.70 (57.54-137.49)	67.15 (39.25-110.17)	415.25 (239.99-652.66)	0.92 (0.81-1.04)
Democratic Socialist Republic of Sri Lanka	460.96 (379.05-569.08)	30.39 (21.47-41.80)	1403.14 (924.68-2006.07)	31.95 (19.52-49.04)	204.40 (85.35-385.43)	0.21 (-0.30-0.73)
Dominican Republic	751.86 (591.04-973.50)	135.26 (91.95-193.93)	2571.87 (1848.11-3388.13)	157.20 (102.58-230.01)	242.07 (134.30-396.95)	0.64 (0.07-1.21)
Eastern Republic of Uruguay	4072.67 (3783.29-4401.41)	594.21 (504.86-690.47)	4204.42 (3840.32-4546.71)	438.38 (369.99-511.85)	3.24 (-7.84-14.87)	-1.00 (-1.30 to -0.71)
Federal Democratic Republic of Ethiopia	3375.46 (1968.08-6141.67)	118.71 (64.55-229.59)	6615.56 (3373.42-11346.26)	105.26 (52.64-182.77)	95.99 (24.85-223.01)	-0.37 (-0.48 to -0.27)
Federal Democratic Republic of Nepal	839.75 (448.54-1525.14)	62.92 (30.51-120.69)	4021.87 (2727.63-6302.31)	111.72 (65.03-188.95)	378.93 (210.38-654.51)	1.86 (1.74-1.99)
Federal Republic of Germany	71699.61 (65797.82-76898.15)	318.86 (272.58-368.39)	73648.63 (65923.92-79177.39)	214.38 (179.56-249.15)	2.72 (-7.71-13.83)	-1.20 (-1.60 to -0.80)
Federal Republic of Nigeria	15787.20 (7876.40-29533.65)	243.05 (120.45-451.40)	26317.64 (12027.71-48315.50)	210.65 (96.86-381.03)	66.70 (13.89-133.16)	-0.46 (-0.59 to -0.32)
Federal Republic of Somalia	373.36 (196.27-787.56)	124.66 (57.08-273.28)	708.92 (319.76-1715.53)	86.24 (35.87-214.68)	89.88 (22.24-193.56)	-1.17 (-1.30 to -1.04)
Federated States of Micronesia	24.95 (17.89-35.11)	360.54 (216.88-575.56)	32.04 (22.78-43.10)	306.42 (191.35-461.69)	28.42 (-6.78-87.44)	-0.53 (-0.58 to -0.48)
Federative Republic of Brazil	47531.56 (45445.63-49251.15)	333.36 (308.98-355.40)	169001.02 (156328.66-178330.82)	398.21 (359.91-430.37)	255.56 (233.78-274.94)	0.60 (0.42-0.78)
French Republic	50824.94 (47641.60-53892.04)	346.69 (293.25-404.33)	45363.12 (40063.92-49021.86)	175.93 (144.97-207.68)	-10.75 (-19.59 to -2.34)	-2.12 (-2.45 to -1.80)

Gabonese Republic	393.39 (245.15-558.96)	430.96 (244.38-689.79)	587.61 (357.56-868.55)	377.57 (209.95-617.41)	49.37 (-1.46-114.34)	-0.42 (-0.52 to -0.32)
Georgia	655.61 (549.26-789.07)	60.05 (48.66-73.53)	3088.43 (2583.50-3633.69)	292.02 (239.42-352.70)	371.08 (250.92-511.61)	4.93 (3.69-6.19)
Grand Duchy of Luxembourg	401.72 (374.14-433.57)	419.29 (359.20-486.52)	388.09 (345.28-430.62)	207.80 (172.42-246.55)	-3.39 (-14.09-9.31)	-2.34 (-2.88 to -1.79)
Greenland	18.12 (15.66-20.54)	396.20 (287.42-533.06)	20.55 (16.14-26.57)	180.07 (122.60-261.89)	13.40 (-12.30-46.77)	-2.39 (-2.95 to -1.84)
Grenada	61.24 (52.98-73.25)	477.07 (391.14-593.95)	84.86 (73.37-95.70)	452.50 (370.31-542.57)	38.58 (11.32-68.76)	-0.13 (-0.8-0.54)
Guam	66.46 (56.18-78.20)	634.87 (460.99-843.73)	62.42 (52.82-73.24)	168.59 (123.03-229.25)	-6.08 (-26.10-22.01)	-4.33 (-5.19 to -3.46)
Hashemite Kingdom of Jordan	219.22 (163.02-293.67)	109.36 (74.26-158.57)	1030.23 (768.19-1347.20)	90.80 (60.97-131.15)	369.95 (194.15-650.88)	-0.56 (-0.88 to -0.25)
Hellenic Republic	9772.05 (9088.26-10422.53)	366.28 (314.20-422.10)	16292.29 (14800.86-17403.08)	394.23 (331.17-459.56)	66.72 (50.86-83.16)	0.22 (-0.42-0.86)
Hungary	7933.41 (7300.95-8583.71)	309.10 (276.49-344.01)	9951.56 (8777.89-11203.75)	295.11 (255.50-338.72)	25.44 (8.96-42.75)	-0.2 (-0.81-0.42)
Independent State of Papua New Guinea	367.28 (231.06-582.00)	143.57 (76.78-247.13)	1020.94 (659.91-1524.02)	145.74 (84.94-234.57)	177.97 (85.40-329.84)	0.05 (-0.09-0.18)
Independent State of Samoa	39.19 (27.87-52.70)	307.74 (183.65-469.06)	56.19 (42.99-72.94)	253.09 (163.19-373.82)	43.37 (10.06-94.25)	-0.64 (-0.68 to -0.60)
Ireland	4550.05 (4259.03-4868.82)	626.80 (541.82-717.93)	3620.32 (3112.88-4016.78)	255.39 (206.69-301.02)	-20.43 (-30.70 to -10.64)	-2.72 (-3.19 to -2.24)
Islamic Republic of Afghanistan	97.93 (50.98-170.29)	8.41 (4.15-15.28)	338.60 (217.11-463.95)	28.58 (16.18-45.42)	245.76 (131.34-461.19)	4.03 (3.90-4.17)
Islamic Republic of Iran	1021.08 (815.38-1297.28)	25.99 (19.59-34.26)	5864.75 (5147.07-6627.64)	48.58 (39.71-58.10)	474.36 (315.09-656.96)	1.93 (1.49-2.36)

Islamic Republic of Mauritania	420.55 (192.33-678.70)	267.31 (120.45-459.08)	741.61 (275.30-1307.78)	226.62 (81.10-456.55)	76.34 (3.02-171.27)	-0.54 (-0.73 to -0.34)
Islamic Republic of Pakistan	7181.97 (4858.89-10950.73)	82.49 (52.00-131.09)	23960.76 (17432.71-33196.77)	139.54 (94.08-201.36)	233.62 (137.71-365.25)	1.71 (1.62-1.81)
Jamaica	533.95 (482.33-601.05)	171.18 (144.64-203.90)	907.04 (702.73-1156.73)	172.56 (127.22-228.01)	69.87 (25.66-121.04)	-0.16 (-0.57-0.26)
Japan	79827.81 (75025.90-82667.11)	276.43 (256.69-290.73)	310349.16 (260095.38-337781.04)	445.86 (387.36-481.29)	288.77 (244.89-313.54)	1.52 (0.96-2.09)
Kingdom of Bahrain	14.95 (12.17-19.28)	62.87 (44.67-88.97)	78.66 (59.06-107.10)	65.96 (43.45-98.86)	426.29 (236.43-675.75)	0.12 (-0.59-0.84)
Kingdom of Belgium	13515.78 (12500.32-14530.71)	492.09 (417.65-570.72)	8891.25 (7802.12-9627.63)	207.00 (170.76-242.24)	-34.22 (-40.62 to -27.40)	-2.99 (-3.16 to -2.81)
Kingdom of Bhutan	25.10 (16.45-37.92)	75.60 (43.10-124.59)	131.26 (82.94-194.05)	137.89 (77.13-232.42)	423.04 (188.03-737.02)	1.97 (1.87-2.07)
Kingdom of Cambodia	340.84 (196.69-537.39)	53.17 (27.68-90.12)	1247.06 (794.38-1991.78)	68.87 (38.87-117.75)	265.88 (142.32-487.75)	0.86 (0.78-0.94)
Kingdom of Denmark	9966.62 (9151.76-10738.29)	699.59 (599.87-809.67)	9152.30 (8197.59-9940.56)	413.14 (347.53-479.94)	-8.17 (-18.42-2.54)	-1.61 (-2.14 to -1.08)
Kingdom of Eswatini	87.81 (58.17-127.95)	225.51 (128.50-368.93)	163.99 (108.01-234.18)	206.90 (119.24-327.61)	86.76 (36.00-165.78)	-0.26 (-0.39 to -0.14)
Kingdom of Lesotho	186.97 (103.81-309.42)	147.92 (72.98-261.89)	289.02 (150.64-471.77)	182.62 (87.59-321.34)	54.58 (5.45-129.44)	0.72 (0.54-0.91)
Kingdom of Morocco	264.72 (173.33-366.04)	11.73 (6.65-18.93)	2323.66 (1592.92-3041.84)	41.42 (25.61-61.53)	777.77 (520.37-1102.44)	4.15 (3.92-4.38)
Kingdom of Norway	9646.36 (9155.18-10039.01)	773.23 (717.28-820.13)	7961.07 (7071.41-8488.72)	431.32 (383.24-467.57)	-17.47 (-22.90 to -12.84)	-1.85 (-2.21 to -1.48)
Kingdom of Saudi Arabia	56.98 (38.81-82.29)	6.98 (4.25-10.91)	464.57 (335.20-638.50)	18.59 (11.92-28.73)	715.26 (409.83-1237.95)	3.22 (2.96-3.48)

Kingdom of Spain	24224.38 (22798.92-25598.55)	249.32 (210.34-293.22)	35143.71 (31750.49-37722.59)	206.70 (170.17-244.96)	45.08 (33.03-56.06)	-0.68 (-1.03 to -0.32)
Kingdom of Sweden	20020.45 (18784.51-21062.13)	734.87 (647.56-822.97)	14339.77 (12495.14-16031.36)	354.14 (294.21-412.74)	-28.37 (-36.14--20.66)	-2.39 (-2.87 to -1.91)
Kingdom of Thailand	7810.67 (5827.94-10485.44)	165.51 (106.68-248.46)	31227.60 (24016.64-40561.38)	167.94 (112.04-238.34)	299.81 (167.13-501.87)	0.07 (-0.14-0.29)
Kingdom of the Netherlands	25959.58 (24110.12-27810.82)	738.60 (632.50-855.37)	20925.19 (18315.59-22691.96)	319.53 (266.56-371.44)	-19.39 (-27.08 to -11.48)	-2.75 (-3.13 to -2.38)
Kingdom of Tonga	22.94 (17.95-29.45)	269.08 (173.57-399.48)	37.02 (25.95-48.82)	284.33 (171.92-438.08)	61.34 (2.33-138.34)	0.18 (-0.02-0.37)
Kyrgyz Republic	132.49 (113.00-155.50)	26.54 (21.59-32.89)	598.29 (470.29-740.84)	76.25 (58.59-95.88)	351.57 (227.28-501.28)	4.30 (3.55-5.06)
Lao People's Democratic Republic	223.63 (139.88-352.31)	74.70 (42.03-121.72)	538.86 (389.95-746.52)	83.84 (53.70-130.14)	140.96 (59.05-291.50)	0.37 (0.33-0.41)
Lebanese Republic	985.81 (489.06-1777.73)	272.92 (120.27-542.39)	2050.89 (1652.17-2596.95)	199.42 (138.10-286.52)	108.04 (7.01-349.87)	-0.98 (-1.17 to -0.78)
Malaysia	4383.32 (3514.72-5376.85)	323.79 (218.13-455.95)	16031.03 (13485.64-19120.59)	368.92 (257.14-525.95)	265.73 (165.71-404.15)	0.35 (0.09-0.62)
Mongolia	52.81 (36.91-73.55)	31.42 (20.97-44.95)	233.17 (174.97-298.85)	66.99 (48.76-89.18)	341.49 (184.92-592.31)	2.44 (2.08-2.80)
Montenegro	678.72 (526.94-889.71)	647.44 (471.97-883.15)	1429.98 (1065.86-1882.37)	835.44 (576.54-1186.62)	110.69 (48.44-210.06)	0.97 (0.79-1.15)
New Zealand	6880.14 (6417.71-7329.81)	994.23 (864.99-1126.74)	5414.75 (4844.04-5842.39)	354.89 (302.99-405.15)	-21.30 (-29.10 to -14.36)	-3.32 (-4.07 to -2.57)
North Macedonia	564.89 (441.20-707.47)	188.40 (135.60-257.67)	1450.33 (909.18-2218.28)	256.65 (152.40-405.52)	156.75 (55.01-299.86)	1.09 (0.76-1.42)
Northern Mariana Islands	9.43 (7.43-12.05)	494.01 (327.22-709.50)	23.74 (18.54-30.80)	299.42 (181.24-468.80)	151.79 (75.73-267.23)	-1.7 (-2.05 to -1.36)

Palestine	81.27 (58.22-111.92)	59.19 (36.38-92.28)	283.03 (215.93-359.30)	74.09 (49.06-108.77)	248.28 (122.39-447.98)	0.66 (0.38-0.95)
People's Democratic Republic of Algeria	245.20 (172.47-334.74)	13.70 (8.28-21.23)	1901.86 (1392.50-2534.92)	35.40 (21.99-54.24)	675.62 (420.68-1044.25)	3.14 (2.97-3.30)
People's Republic of Bangladesh	5473.03 (3123.41-10118.93)	78.08 (38.87-154.76)	23519.85 (15283.13-40305.92)	109.50 (62.34-195.43)	329.74 (184.08-601.68)	1.16 (0.85-1.48)
People's Republic of China	36486.89 (28744.80-47243.64)	27.60 (21.65-35.53)	136337.08 (105147.11-176012.76)	37.22 (28.60-48.23)	273.66 (145.54-453.19)	0.98 (0.88-1.07)
Plurinational State of Bolivia	565.20 (362.96-834.40)	116.76 (65.39-187.31)	1812.08 (1318.02-2494.06)	126.62 (78.21-195.02)	220.61 (110.50-415.70)	0.27 (0.16-0.37)
Portuguese Republic	4056.12 (3782.41-4349.66)	165.87 (141.96-191.72)	6419.99 (5851.36-6907.46)	149.42 (125.44-175.58)	58.28 (42.36-74.11)	-0.28 (-1.29-0.75)
Principality of Andorra	61.76 (39.32-91.53)	649.49 (365.56-1098.96)	96.45 (60.52-149.22)	359.81 (198.80-606.58)	56.18 (-2.66-145.28)	-2.05 (-2.51 to -1.59)
Principality of Monaco	68.51 (50.78-86.28)	526.29 (346.10-757.19)	84.57 (63.74-114.20)	457.26 (292.79-686.05)	23.43 (-12.51-83.59)	-0.47 (-0.59 to -0.35)
Puerto Rico	1312.17 (1220.80-1403.41)	215.47 (184.69-249.37)	1144.53 (943.31-1339.72)	85.55 (67.61-106.33)	-12.78 (-27.33-3.07)	-2.87 (-3.71 to -2.03)
Republic of Albania	283.12 (231.56-341.28)	88.86 (65.69-117.27)	798.59 (506.44-1170.12)	102.60 (57.18-163.98)	182.07 (75.77-352.58)	0.43 (0.12-0.75)
Republic of Angola	1487.21 (806.86-2349.85)	272.52 (140.13-459.84)	4756.97 (2840.39-7210.53)	291.25 (157.94-489.54)	219.86 (104.07-398.60)	0.23 (0.02-0.44)
Republic of Armenia	2089.56 (1697.01-2544.19)	457.50 (366.34-572.14)	6905.43 (5735.48-8145.63)	897.66 (736.65-1078.58)	230.47 (148.98-346.31)	2.18 (1.60-2.76)
Republic of Austria	6573.73 (6187.58-6971.34)	315.20 (272.13-360.46)	5572.78 (4956.92-6002.83)	171.41 (143.75-199.11)	-15.23 (-22.25 to -7.51)	-1.90 (-2.31 to -1.48)
Republic of Azerbaijan	579.80 (435.11-774.96)	70.81 (48.78-100.97)	2462.10 (1455.31-4122.94)	140.99 (80.93-230.86)	324.65 (130.94-627.70)	2.21 (1.74-2.68)

Republic of Belarus	5894.03 (5115.00-7201.38)	258.37 (211.57-321.09)	10880.70 (8895.31-13217.95)	378.09 (295.95-475.37)	84.61 (41.65-135.83)	1.30 (0.49-2.11)
Republic of Benin	476.03 (224.03-857.22)	154.31 (67.25-298.14)	1127.14 (444.61-2073.36)	152.54 (57.62-305.92)	136.78 (65.80-225.77)	-0.04 (-0.13 to 0.06)
Republic of Botswana	185.81 (118.19-297.02)	236.52 (132.34-404.39)	396.61 (243.80-535.62)	198.36 (112.67-306.60)	113.44 (29.21-271.57)	-0.56 (-0.75 to -0.38)
Republic of Bulgaria	3919.98 (3440.67-4380.46)	184.73 (160.44-210.99)	6474.38 (5179.08-8082.34)	265.85 (207.90-337.43)	65.16 (26.81-118.30)	1.17 (0.22-2.13)
Republic of Burundi	846.88 (462.07-1399.43)	236.84 (115.65-429.30)	925.92 (406.08-1762.96)	137.47 (56.23-274.51)	9.33 (-47.69-80.92)	-1.75 (-1.82 to -1.68)
Republic of Cabo Verde	71.30 (28.35-146.91)	174.52 (61.57-374.28)	161.16 (77.91-287.65)	231.80 (102.38-442.44)	126.03 (59.04-258.55)	0.88 (0.35-1.41)
Republic of Cameroon	1517.18 (982.77-2668.40)	235.89 (133.02-434.65)	4044.66 (2390.19-7039.95)	226.91 (123.11-401.11)	166.59 (79.79-298.57)	-0.13 (-0.25 to -0.01)
Republic of Chad	605.36 (281.63-1275.18)	135.63 (58.90-296.64)	1216.09 (517.66-2420.66)	147.52 (57.30-315.47)	100.89 (21.46-208.22)	0.29 (0.19-0.40)
Republic of Chile	4381.84 (4138.46-4636.19)	268.28 (232.86-307.76)	9438.16 (8655.00-10147.61)	211.26 (178.35-245.77)	115.39 (95.90-135.08)	-0.83 (-1.54 to -0.13)
Republic of Colombia	9699.07 (9097.33-10338.39)	353.00 (306.21-404.82)	27050.21 (22202.73-32496.71)	289.36 (227.27-359.99)	178.89 (130.66-236.62)	-0.58 (-1.36-0.22)
Republic of Costa Rica	639.83 (582.11-713.34)	229.45 (194.20-270.36)	2161.41 (1886.67-2449.16)	230.79 (188.61-276.14)	237.81 (182.48-295.92)	0.05 (-0.32-0.41)
Republic of Croatia	2729.92 (2357.64-3180.58)	265.59 (227.98-310.24)	5333.88 (4511.64-6246.18)	334.82 (275.93-397.27)	95.39 (52.79-144.92)	0.55 (-0.39-1.49)
Republic of Cuba	9290.83 (8559.97-9994.30)	538.65 (465.93-618.47)	12373.00 (10481.30-14016.77)	360.11 (296.04-430.51)	33.17 (14.54-55.66)	-1.33 (-1.72 to -0.94)
Republic of Cyprus	837.86 (650.43-1069.19)	681.57 (428.93-1011.69)	1291.44 (969.14-1622.35)	360.48 (237.98-525.34)	54.13 (5.67-132.26)	-2.05 (-2.51 to -1.59)

Republic of Côte d'Ivoire	1330.00 (620.33-2371.46)	250.32 (108.37-476.20)	3637.91 (1607.52-6496.72)	236.52 (96.68-461.68)	173.53 (88.41-284.61)	-0.19 (-0.31 to -0.06)
Republic of Djibouti	38.80 (19.99-61.67)	219.17 (104.44-383.29)	161.62 (69.43-271.27)	188.25 (76.43-341.37)	316.53 (159.06-546.51)	-0.47 (-0.60 to -0.35)
Republic of Ecuador	1014.15 (932.70-1100.55)	124.67 (105.60-145.31)	3192.73 (2488.66-4094.11)	118.62 (88.61-158.37)	214.82 (141.28-314.38)	-0.27 (-1.53-1.01)
Republic of El Salvador	243.99 (201.42-290.68)	51.09 (36.86-69.42)	530.47 (409.60-685.84)	50.93 (34.69-72.61)	117.41 (53.21-195.68)	-0.01 (-0.87-0.87)
Republic of Equatorial Guinea	79.80 (44.35-131.41)	276.36 (145.13-491.74)	232.79 (110.75-402.04)	334.22 (151.40-612.53)	191.71 (46.40-439.40)	0.66 (0.43-0.88)
Republic of Estonia	1011.59 (919.92-1114.74)	284.88 (245.57-329.09)	1559.40 (1354.10-1779.40)	327.42 (272.64-387.07)	54.15 (30.59-82.76)	0.55 (-0.43-1.53)
Republic of Fiji	156.57 (123.96-191.38)	321.92 (223.38-447.98)	371.79 (274.59-487.58)	328.11 (221.38-467.94)	137.46 (65.98-246.87)	0.07 (-0.27-0.40)
Republic of Finland	9943.54 (9254.32-10600.90)	795.65 (682.05-914.84)	7842.73 (6952.38-8506.82)	335.47 (278.95-391.68)	-21.13 (-28.84 to -12.75)	-2.85 (-3.02 to -2.69)
Republic of Ghana	2723.61 (1327.08-4563.76)	302.49 (136.29-544.56)	6716.46 (3042.90-11208.03)	277.97 (119.91-507.95)	146.60 (65.47-258.51)	-0.27 (-0.44 to -0.09)
Republic of Guatemala	279.80 (252.49-309.65)	55.42 (46.61-65.70)	705.19 (594.08-841.11)	39.92 (31.58-49.19)	152.04 (106.20-208.56)	-1.01 (-2.17-0.16)
Republic of Guinea	871.68 (420.40-1670.80)	165.88 (74.64-342.81)	1546.28 (571.39-3023.38)	184.09 (63.58-388.90)	77.39 (0.28-163.71)	0.33 (0.26-0.40)
Republic of Guinea-Bissau	145.72 (77.96-264.49)	248.25 (120.99-476.11)	215.87 (114.73-389.81)	218.88 (106.40-415.70)	48.14 (3.85-116.55)	-0.39 (-0.50 to -0.28)
Republic of Guyana	118.23 (104.89-131.64)	204.13 (172.35-238.28)	361.66 (275.74-462.47)	349.54 (256.71-460.37)	205.89 (126.54-294.84)	1.88 (0.83-2.95)
Republic of Haiti	1119.97 (717.19-1746.83)	236.38 (135.41-390.95)	2348.98 (1433.77-3685.83)	228.48 (127.59-385.47)	109.74 (48.07-195.98)	-0.1 (-0.22-0.03)

Republic of Honduras	237.07 (174.99-330.23)	76.63 (48.70-116.78)	1145.94 (807.64-1575.38)	116.64 (70.70-179.62)	383.38 (231.37-649.59)	1.41 (1.13-1.69)
Republic of Iceland	209.64 (187.98-227.00)	416.93 (354.69-480.98)	243.77 (210.05-273.19)	230.79 (187.46-272.66)	16.28 (2.58-32.60)	-1.91 (-2.17 to -1.66)
Republic of India	46761.40 (27838.63-77331.29)	67.88 (40.28-112.29)	222689.03 (158767.07-326391.51)	117.64 (82.63-172.71)	376.22 (254.08-575.37)	1.83 (1.48-2.18)
Republic of Indonesia	8148.14 (5488.55-10985.70)	59.80 (38.04-84.18)	30671.96 (20556.48-41544.06)	92.71 (60.80-128.39)	276.43 (139.50-441.17)	1.44 (1.39-1.49)
Republic of Iraq	342.50 (242.51-477.31)	27.39 (17.09-41.52)	1358.17 (945.59-1794.82)	36.57 (23.13-53.68)	296.54 (139.21-562.53)	0.97 (0.77-1.18)
Republic of Italy	48841.61 (46473.44-50571.42)	309.89 (287.02-329.48)	53536.25 (47925.89-57360.68)	202.70 (180.68-219.60)	9.61 (1.67-16.34)	-1.39 (-1.73 to -1.05)
Republic of Kazakhstan	3023.17 (2439.01-3856.65)	146.27 (116.37-187.87)	6971.15 (5577.06-8511.72)	228.19 (180.33-280.99)	130.59 (65.39-223.69)	1.44 (0.73-2.16)
Republic of Kenya	1641.66 (1043.45-2385.62)	136.75 (86.63-201.31)	5503.44 (3238.30-7712.61)	169.29 (98.33-239.63)	235.24 (142.06-331.59)	0.7 (0.59-0.82)
Republic of Kiribati	2.13 (1.73-2.60)	38.94 (26.09-56.31)	4.54 (3.24-6.17)	41.81 (26.11-63.14)	113.46 (41.45-202.93)	0.23 (0.19-0.27)
Republic of Korea	6846.55 (4516.73-9852.18)	165.94 (100.38-255.27)	25872.02 (20687.08-30999.92)	159.91 (113.03-213.38)	277.88 (131.82-541.20)	-0.14 (-0.30-0.03)
Republic of Latvia	1399.32 (1283.79-1533.01)	224.86 (194.26-259.97)	2092.95 (1807.10-2378.09)	303.57 (251.12-362.37)	49.57 (27.62-75.04)	1.12 (0.30-1.95)
Republic of Liberia	383.75 (198.51-705.97)	212.98 (101.36-418.48)	533.05 (229.64-1036.90)	187.66 (75.85-394.47)	38.91 (-8.53-95.37)	-0.41 (-0.69 to -0.13)
Republic of Lithuania	1640.22 (1497.08-1811.98)	208.88 (181.04-240.92)	3118.27 (2696.75-3556.88)	311.83 (257.92-372.54)	90.11 (62.36-122.07)	1.14 (0.91-1.37)
Republic of Madagascar	2305.14 (1137.25-4022.49)	298.42 (133.32-555.80)	3656.68 (1798.89-6130.58)	238.72 (106.80-429.57)	58.63 (11.82-127.31)	-0.75 (-1.03 to -0.47)

Republic of Malawi	817.80 (389.87-1587.19)	145.29 (62.71-294.20)	1934.66 (922.63-3371.20)	180.03 (80.95-332.94)	136.57 (68.96-234.18)	0.70 (0.57-0.83)
Republic of Maldives	7.42 (5.12-10.53)	61.64 (37.39-99.33)	23.52 (11.07-39.11)	50.96 (23.49-93.86)	216.84 (73.83-434.03)	-0.73 (-0.92 to -0.54)
Republic of Mali	694.19 (349.58-1424.71)	119.91 (55.19-254.44)	1443.05 (604.49-2878.36)	113.79 (44.99-240.25)	107.88 (17.14-251.00)	-0.16 (-0.36-0.03)
Republic of Malta	189.14 (174.96-205.33)	258.99 (218.89-302.88)	223.46 (198.87-249.64)	124.63 (101.94-150.36)	18.14 (3.58-34.63)	-2.55 (-3.61 to -1.49)
Republic of Mauritius	150.14 (140.38-159.90)	138.74 (119.36-159.76)	242.24 (222.22-258.74)	78.52 (66.01-91.61)	61.35 (46.70-74.91)	-1.93 (-3.21 to -0.63)
Republic of Moldova	677.53 (603.76-754.44)	92.00 (79.08-106.71)	1662.86 (1459.39-1881.19)	156.98 (132.27-185.06)	145.43 (106.09-194.46)	1.86 (0.43-3.31)
Republic of Mozambique	1560.59 (672.48-3154.37)	180.93 (72.34-378.07)	3749.41 (1530.17-7307.35)	234.76 (90.32-480.17)	140.26 (63.26-232.31)	0.85 (0.77-0.93)
Republic of Namibia	220.47 (142.61-336.96)	238.35 (138.10-392.29)	439.04 (315.03-606.16)	226.42 (139.19-355.41)	99.13 (40.36-193.43)	-0.15 (-0.23 to -0.08)
Republic of Nauru	3.11 (2.34-4.07)	483.01 (297.76-754.29)	3.80 (2.58-5.15)	521.26 (252.84-908.74)	22.32 (-13.94-70.66)	0.24 (0.18-0.30)
Republic of Nicaragua	78.65 (65.49-93.85)	33.39 (23.87-45.47)	265.23 (207.84-334.68)	34.15 (23.78-48.33)	237.25 (147.90-346.95)	0.07 (-0.08-0.23)
Republic of Niue	1.28 (1.02-1.59)	328.46 (214.22-479.80)	1.06 (0.80-1.33)	289.14 (188.50-420.81)	-17.60 (-38.47-7.98)	-0.42 (-0.48 to -0.36)
Republic of Palau	4.25 (3.19-5.43)	298.57 (193.08-441.38)	8.34 (6.29-11.22)	249.56 (159.83-378.79)	96.24 (36.90-186.61)	-0.61 (-0.70 to -0.53)
Republic of Panama	536.41 (486.68-584.85)	225.04 (188.13-264.27)	1276.53 (965.98-1559.62)	171.74 (126.66-218.07)	137.97 (81.80-192.28)	-0.74 (-1.46-0.00)
Republic of Paraguay	727.50 (583.54-902.57)	204.31 (146.26-280.58)	2842.13 (2159.31-3693.31)	295.93 (201.70-416.94)	290.67 (179.17-465.40)	1.16 (0.79-1.54)

Republic of Peru	1334.65 (1018.56-1743.91)	71.83 (47.22-105.70)	3338.10 (2465.83-4503.13)	60.52 (38.67-91.05)	150.11 (66.08-266.03)	-0.40 (-1.07-0.28)
Republic of Poland	34785.66 (33664.27-35781.72)	457.93 (436.48-476.85)	43033.02 (38697.43-47583.47)	334.95 (296.62-373.79)	23.71 (12.05-36.85)	-1.24 (-1.62 to -0.86)
Republic of Rwanda	1122.19 (656.83-1782.43)	268.13 (139.53-463.84)	1579.76 (934.28-2641.25)	174.69 (94.54-315.24)	40.77 (-15.40-134.39)	-1.38 (-1.66 to -1.09)
Republic of San Marino	18.49 (14.79-22.91)	293.71 (194.32-432.80)	21.60 (13.73-33.27)	159.57 (83.95-272.02)	16.81 (-30.09-85.31)	-2.3 (-2.59 to -2.00)
Republic of Senegal	1035.01 (479.63-1912.71)	210.38 (89.93-423.82)	2374.13 (997.37-4398.23)	204.52 (80.62-405.32)	129.38 (63.19-211.91)	-0.13 (-0.44 to 0.18)
Republic of Serbia	7139.20 (6014.30-8447.30)	398.86 (290.84-532.77)	12127.37 (9565.74-15305.84)	411.75 (287.04-570.72)	69.87 (28.12-128.61)	0.02 (-0.22-0.25)
Republic of Seychelles	9.54 (7.83-11.53)	98.97 (68.60-139.88)	17.17 (11.37-24.62)	95.51 (54.76-156.43)	79.97 (24.54-155.14)	-0.27 (-0.97-0.44)
Republic of Sierra Leone	683.40 (321.30-1290.65)	211.58 (92.03-425.85)	1017.44 (438.62-1999.17)	183.64 (72.17-382.30)	48.88 (1.38-116.24)	-0.45 (-0.52 to -0.38)
Republic of Singapore	917.52 (866.09-966.46)	269.36 (233.06-308.49)	2788.71 (2532.85-2992.03)	193.70 (163.56-223.48)	203.94 (174.59-231.37)	-1.01 (-1.24 to -0.77)
Republic of Slovenia	1159.14 (1078.81-1243.19)	271.80 (234.17-312.26)	1673.58 (1401.58-2011.83)	209.73 (168.16-261.18)	44.38 (21.45-73.56)	-0.94 (-1.63 to -0.24)
Republic of South Africa	8246.38 (6254.80-10341.77)	266.31 (192.97-347.67)	14598.19 (12774.64-16441.73)	204.70 (171.55-243.39)	77.03 (45.10-135.13)	-0.84 (-1.21 to -0.48)
Republic of South Sudan	784.16 (305.78-1467.64)	194.51 (71.94-392.62)	720.77 (307.63-1417.20)	135.45 (53.34-271.43)	-8.08 (-37.66-35.68)	-1.17 (-1.28 to -1.06)
Republic of Sudan	137.80 (73.16-320.80)	9.41 (4.40-23.16)	919.70 (583.35-1371.39)	31.51 (17.90-50.67)	567.41 (222.41-1129.98)	3.99 (3.89-4.09)
Republic of Suriname	95.98 (79.55-114.64)	240.18 (168.89-335.78)	213.69 (149.09-293.60)	203.03 (124.85-312.80)	122.63 (46.19-219.66)	-0.46 (-0.96-0.04)

Republic of Tajikistan	118.11 (85.14-151.16)	26.76 (16.60-41.17)	210.68 (150.58-288.52)	24.28 (15.63-36.43)	78.37 (14.60-172.17)	-0.31 (-0.82-0.19)
Republic of the Congo	648.08 (411.98-962.85)	400.94 (230.08-649.14)	1251.71 (746.22-1926.76)	336.18 (186.28-561.99)	93.14 (34.61-173.22)	-0.55 (-0.67 to -0.42)
Republic of the Gambia	111.28 (45.75-220.07)	219.09 (84.96-455.33)	339.75 (155.36-595.98)	237.07 (98.68-456.49)	205.32 (116.42-347.89)	0.25 (-0.10-0.59)
Republic of the Marshall Islands	6.31 (4.15-9.23)	283.45 (164.02-464.72)	12.48 (8.18-17.99)	262.07 (154.50-409.88)	97.84 (43.92-176.52)	-0.26 (-0.37 to -0.15)
Republic of the Niger	484.42 (204.93-1032.60)	129.99 (52.03-296.94)	1304.05 (481.68-3019.10)	111.97 (39.47-273.81)	169.19 (71.53-282.16)	-0.47 (-0.66 to -0.28)
Republic of the Philippines	4332.90 (3615.08-5125.02)	106.84 (86.49-129.05)	15099.89 (12223.85-17853.92)	124.01 (99.78-149.13)	248.49 (180.79-349.12)	0.49 (0.24-0.74)
Republic of the Union of Myanmar	2554.18 (1473.26-3798.30)	75.30 (40.61-124.73)	6315.21 (4755.68-8381.48)	86.57 (55.26-128.86)	147.25 (62.41-300.46)	0.46 (0.43-0.50)
Republic of Trinidad and Tobago	711.00 (655.82-781.33)	531.13 (449.96-620.82)	1274.28 (973.79-1630.21)	378.15 (278.96-499.27)	79.22 (33.04-131.24)	-0.94 (-1.43 to -0.44)
Republic of Tunisia	113.67 (78.65-153.97)	14.02 (8.47-21.47)	878.18 (566.25-1287.81)	40.05 (23.29-64.82)	672.57 (382.04-1152.72)	3.42 (3.23-3.61)
Republic of Turkey	11599.70 (8213.18-16710.25)	202.52 (123.69-316.07)	35705.49 (27769.20-45418.97)	221.67 (154.07-316.32)	207.81 (90.11-377.43)	0.29 (-0.06-0.63)
Republic of Uganda	1462.64 (671.00-2819.85)	152.20 (64.76-311.23)	3185.62 (1517.06-5463.56)	152.98 (69.43-281.77)	117.80 (44.41-235.14)	0.01 (-0.10-0.11)
Republic of Uzbekistan	379.93 (281.35-529.33)	20.21 (14.79-28.74)	3955.82 (3138.88-4915.55)	99.91 (78.89-124.96)	941.20 (593.49-1412.90)	5.07 (4.07-6.07)
Republic of Vanuatu	17.56 (12.02-26.17)	204.09 (115.07-339.30)	48.22 (35.61-65.88)	197.47 (121.92-305.14)	174.54 (101.93-281.86)	-0.12 (-0.26-0.01)
Republic of Yemen	75.86 (38.81-134.43)	9.82 (4.71-18.30)	670.15 (388.47-1063.08)	31.61 (16.52-54.13)	783.40 (453.30-1391.27)	3.87 (3.63-4.12)

Republic of Zambia	858.11 (543.07-1318.78)	214.46 (118.00-371.28)	3171.58 (1355.68-5651.29)	329.00 (134.51-617.58)	269.60 (86.45-579.92)	1.39 (1.21-1.58)
Republic of Zimbabwe	1783.18 (1382.67-2184.00)	312.21 (210.81-450.08)	3534.25 (2622.20-4711.78)	371.85 (236.06-542.87)	98.20 (34.62-188.67)	0.62 (0.34-0.90)
Romania	6164.38 (5658.66-6712.14)	128.88 (112.37-147.88)	12029.87 (10428.46-13920.16)	192.09 (159.02-229.49)	95.15 (65.83-130.19)	1.42 (0.56-2.29)
Russian Federation	82296.31 (79962.64-84208.55)	265.53 (254.99-274.31)	182953.28 (167026.09-199483.27)	428.82 (384.58-472.53)	122.31 (102.33-142.18)	2.01 (1.03-2.99)
Saint Kitts and Nevis	24.74 (22.20-27.52)	357.79 (305.04-416.86)	25.66 (20.69-32.40)	240.37 (187.47-311.87)	3.72 (-18.26-31.68)	-1.18 (-1.89 to -0.47)
Saint Lucia	127.74 (116.24-142.54)	916.41 (772.86-1081.36)	173.98 (144.70-204.43)	436.16 (343.12-540.87)	36.19 (10.20-66.44)	-2.41 (-3.02 to -1.80)
Saint Vincent and the Grenadines	29.46 (27.09-32.38)	241.20 (204.65-281.72)	46.20 (40.51-52.58)	193.55 (159.66-232.30)	56.83 (35.45-86.39)	-0.66 (-1.17 to -0.16)
Slovak Republic	2369.35 (2006.47-2816.62)	227.30 (172.87-298.92)	3880.45 (2925.29-5078.59)	231.34 (158.25-328.37)	63.78 (13.20-134.62)	0.10 (-0.30-0.49)
Socialist Republic of Viet Nam	3814.25 (2712.93-5423.16)	60.44 (37.25-94.51)	14416.57 (10464.75-19485.79)	96.79 (60.07-148.50)	277.97 (152.14-495.12)	1.54 (1.48-1.61)
Solomon Islands	28.57 (18.35-48.84)	160.65 (88.49-288.37)	76.13 (51.02-112.37)	171.95 (98.40-285.37)	166.47 (82.47-274.32)	0.21 (0.06-0.37)
State of Eritrea	276.04 (142.13-507.09)	181.04 (85.07-357.17)	658.04 (296.08-1230.92)	180.41 (74.19-362.44)	138.38 (38.10-315.71)	-0.03 (-0.21-0.14)
State of Israel	1863.48 (1718.89-2002.58)	222.50 (190.16-257.74)	2598.35 (2315.52-2822.60)	118.83 (98.00-139.71)	39.44 (24.19-57.78)	-1.99 (-2.29 to -1.70)
State of Kuwait	81.30 (72.14-90.73)	95.16 (79.47-113.14)	319.84 (260.21-397.84)	80.78 (61.21-103.35)	293.42 (205.73-404.53)	0.09 (-3.02-3.30)
State of Libya	30.91 (21.45-45.96)	10.54 (6.24-17.46)	236.43 (145.96-378.56)	30.68 (16.85-51.66)	664.78 (387.36-1108.32)	3.57 (3.20-3.94)

State of Qatar	17.28 (13.18-22.67)	144.78 (95.60-211.49)	105.39 (54.21-196.42)	94.57 (48.14-179.74)	509.86 (170.98-1247.91)	-1.29 (-1.99 to -0.59)
Sultanate of Oman	16.82 (10.24-27.60)	17.23 (9.19-30.38)	161.36 (89.19-275.94)	57.07 (28.12-110.11)	859.56 (258.95-2281.62)	3.94 (3.42-4.45)
Swiss Confederation	8818.00 (8092.05-9542.66)	481.99 (405.94-565.42)	6927.60 (6046.46-7596.65)	204.81 (166.84-240.23)	-21.44 (-30.40--11.63)	-2.7 (-2.90 to -2.51)
Syrian Arab Republic	272.97 (189.83-380.65)	33.01 (20.63-50.48)	865.09 (624.96-1187.63)	39.78 (25.29-59.83)	216.92 (93.96-407.72)	0.51 (0.32-0.70)
Taiwan (Province of China)	3283.22 (3062.19-3500.03)	125.44 (107.50-145.38)	15504.76 (13926.08-16877.42)	207.39 (173.57-244.05)	372.24 (322.47-421.58)	1.76 (0.88-2.64)
Togolese Republic	406.52 (197.07-742.67)	232.54 (101.96-461.09)	1207.36 (502.07-2182.26)	225.05 (88.27-452.15)	197.00 (102.21-300.87)	-0.11 (-0.21-0.00)
Tokelau	0.70 (0.54-0.89)	314.34 (194.09-477.80)	0.75 (0.50-1.04)	292.14 (156.71-488.03)	6.74 (-33.10-53.77)	-0.25 (-0.40 to -0.09)
Turkmenistan	314.18 (259.67-381.84)	101.30 (81.83-125.59)	1214.09 (897.29-1750.73)	184.30 (135.73-265.44)	286.44 (153.75-470.28)	2.09 (1.59-2.59)
Tuvalu	2.79 (1.82-3.94)	285.13 (165.58-461.44)	4.24 (3.34-5.42)	264.04 (176.81-380.62)	52.21 (10.46-120.11)	-0.25 (-0.29 to -0.20)
Ukraine	26969.12 (24339.36-29973.14)	214.96 (182.68-254.20)	33152.28 (24364.88-43318.84)	242.06 (172.99-326.20)	22.93 (-13.30-64.56)	0.46 (-0.34-1.27)
Union of the Comoros	67.40 (25.44-124.62)	230.58 (87.96-446.17)	136.42 (51.49-252.31)	186.33 (65.03-372.04)	102.41 (29.32-205.34)	-0.71 (-0.95 to -0.46)
United Arab Emirates	66.31 (38.68-103.37)	131.05 (69.37-217.91)	591.20 (459.06-787.02)	147.90 (99.41-213.09)	791.57 (451.91-1552.64)	0.21 (-1.22-1.66)
United Kingdom of Great Britain and Northern Ireland	158249.66 (151615.20-161878.81)	958.17 (912.84-988.90)	85116.94 (77181.19-89620.41)	346.94 (314.73-367.09)	-46.21 (-49.42 to -44.31)	-3.27 (-3.52 to -3.01)
United Mexican States	4457.25 (4320.23-4569.97)	70.71 (66.89-74.66)	11941.18 (10468.10-13552.46)	58.13 (50.25-66.72)	167.90 (135.28-205.09)	-0.69 (-1.47 to 0.10)

United Republic of Tanzania		3580.28 (2134.38-5998.23)	219.00 (115.92-395.19)	9067.93 (4331.33-15664.07)	243.18 (107.21-466.68)	153.27 (45.15-345.55)	0.35 (0.25-0.45)
United States of America	291231.59 (274421.51-300868.52)	515.28 (479.94-538.92)	188420.24 (172461.27-197574.71)	180.83 (164.29-192.20)	-35.30 (-37.61 to -33.27)	-3.37 (-3.80 to -2.93)
United States Virgin Islands	39.21 (31.66-47.03)	312.82 (215.42-431.68)	59.00 (43.54-75.62)	179.59 (116.75-263.84)	50.46 (7.00-110.28)	-1.84 (-2.27 to -1.42)

DALYs, disability-adjusted life years; AA, aortic aneurysm; AAPC, average annual percentage change; ASDR, age-standardized disability-adjusted life years rate; UI, uncertainty intervals; CI, confidence interval.

Table 6. Deaths and DALYs of AA with decomposition analysis, categorized by global and SDI regions.

Measures	Location	Overall difference	Aging	Population	Epidemiological change	Aging percentage	Population percentage	Epidemiological change percentage
Deaths	Global	58842.64	12608.15	86523.32	-40288.84	21.43	147.04	-68.47
	High SDI	12601.97	-2575.01	47793.96	-32616.98	-20.43	379.26	-258.82
	High-middle SDI	15180.14	72.67	17869.03	-2761.56	0.48	117.71	-18.19
	Middle SDI	17395.42	4614.62	10791.04	1989.75	26.53	62.03	11.44
	Low-middle SDI	10528.99	1273.87	6165.40	3089.73	12.10	58.56	29.34
	Low SDI	3079.65	157.21	2712.64	209.79	5.10	88.08	6.81
DALYs	Global	923091.18	109610.81	1555428.38	-741948.01	11.87	168.50	-80.38
	High SDI	67532.95	-111962.70	794872.28	-615376.64	-165.79	1177.01	-911.22
	High-middle SDI	263524.87	-26314.82	348556.81	-58717.12	-9.99	132.27	-22.28
	Middle SDI	327033.02	71697.89	211008.28	44326.84	21.92	64.52	13.55
	Low-middle SDI	203317.63	19536.33	123545.64	60235.67	9.61	60.76	29.63
	Low SDI	60,826.20	1157.97	56085.39	3582.84	1.90	92.21	5.89

DALYs, disability-adjusted life years rate; AA, aortic aneurysm; SDI, sociodemographic index.

Table 7. ASMR and ASDR of AA with frontier analysis across all countries and territories.

Location	SDI	Death			DALYs		
		Frontier	Effective difference	Effective difference rank	Frontier	Effective difference	Effective difference rank
Afghanistan	0.337199998	0.4208624	0.9963134	4	8.508961	20.0752032	4
Albania	0.706849791	0.3924941	5.4209115	44	7.2582388	95.3371562	45
Algeria	0.659500924	0.3884117	1.7065388	13	7.2916109	28.1067905	11
American Samoa	0.723727533	0.390363	13.8134575	141	7.2357329	234.3502414	139
Andorra	0.869444113	0.3920055	21.8118565	188	7.2532021	352.557089	180
Angola	0.453721949	0.4204622	15.1622597	156	8.5070108	282.7386559	157
Antigua and Barbuda	0.749886887	0.3915987	10.5567382	94	7.3240919	182.1970186	94
Argentina	0.723122973	0.3913405	13.8263938	142	7.2620088	261.3710535	150
Armenia	0.701833194	0.3888557	48.461197	204	7.2242389	890.4344773	204
Australia	0.844252814	0.3890713	12.6401321	136	7.2701315	197.0769761	106
Austria	0.853837004	0.3902489	9.5513745	80	7.2482649	164.1632743	76
Azerbaijan	0.694851274	0.3921054	6.6230883	53	7.2230916	133.7620207	61
Bahamas	0.805020668	0.3894023	15.0016111	153	7.2865315	265.1060515	151
Bahrain	0.753043204	0.3891526	3.3403167	28	7.240685	58.7154877	28
Bangladesh	0.492420885	0.4210381	6.1997532	51	8.5120483	100.9861226	47
Barbados	0.746748764	0.39187	11.9412703	122	7.2434897	197.4051318	107
Belarus	0.784484711	0.3907142	17.6618816	168	7.299578	370.7928244	186
Belgium	0.853654016	0.3908832	12.0526678	124	7.255317	199.7494492	112
Belize	0.610229002	0.3942044	4.4454415	36	7.2364539	77.5130881	36
Benin	0.373486574	0.421281	8.2202587	69	8.5182343	144.0197088	66
Bermuda	0.821365422	0.3899314	23.0375182	194	7.2484311	372.4564688	189
Bhutan	0.473062378	0.4213117	7.7576704	66	8.4893791	129.4028856	59
Bolivia (Plurinational State of)	0.599010799	0.3884718	6.812441	56	7.2732601	119.3517142	56
Bosnia and Herzegovina	0.723077893	0.3890853	15.6384528	160	7.2532213	300.2002111	165

Botswana	0.642721629	0.3917067	11.5064637	118	7.2354927	191.128833	101
Brazil	0.653043887	0.3901119	20.4701115	179	7.2491002	390.9628712	191
Brunei Darussalam	0.810234367	0.3908805	25.1511102	196	7.2561184	420.2257952	194
Bulgaria	0.768150939	0.3920962	12.5531724	133	7.2738186	258.5773898	149
Burkina Faso	0.285118402	0.420306	10.8094734	98	8.4949318	187.8509513	99
Burundi	0.289374365	0.4195669	7.2795764	60	8.5120388	128.9615801	58
Cabo Verde	0.533534539	0.4193585	12.7978768	137	8.5366563	223.2622647	130
Cambodia	0.473621491	0.4219424	3.6451972	32	8.4914983	60.3754872	30
Cameroon	0.479691223	0.4202275	12.0397576	123	8.516228	218.3949449	126
Canada	0.87317068	0.3919879	11.734748	119	7.2661543	186.0158496	96
Central African Republic	0.30916769	0.4193021	10.9675982	105	8.501076	209.8283815	121
Chad	0.240436019	0.4192169	7.7325872	65	8.5127517	139.0025484	63
Chile	0.771514716	0.3935341	11.0268926	107	7.2957856	203.9600876	117
China	0.72162976	0.391784	1.5758929	10	7.295669	29.9276357	13
Colombia	0.655442913	0.3889463	15.3334959	158	7.2259489	282.132294	156
Comoros	0.475978688	0.4200294	10.0508504	90	8.4924344	177.8412657	91
Congo	0.583075236	0.3921476	17.757552	169	7.2583266	328.9263788	175
Cook Islands	0.779109955	0.3923992	15.0253629	154	7.3087104	252.0452348	146
Costa Rica	0.700340477	0.3897345	12.233592	129	7.230558	223.5596902	132
Côte d'Ivoire	0.425941883	0.4216227	12.5902263	134	7.2680441	327.5554028	172
Croatia	0.798341027	0.3908166	17.8210636	172	7.3021063	352.8033471	181
Cuba	0.668729864	0.3916764	20.5885891	180	7.3015083	353.1780982	182
Cyprus	0.835630545	0.3926573	21.940723	190	7.2832521	271.8935314	153
Czechia	0.828450433	0.3906762	14.6784289	150	8.5294381	227.993994	135
Democratic People's Republic of Korea	0.569854634	0.3911383	1.7719396	14	7.4294554	36.9506183	20
Democratic Republic of the Congo	0.383179849	0.4202955	11.1945346	110	8.5336666	208.7528992	120
Denmark	0.896424204	0.3904066	25.6431315	197	7.2780399	405.8587108	193
Djibouti	0.487958371	0.4198614	9.9462523	88	8.4822349	179.7686009	93
Dominica	0.746967185	0.3929021	21.7611931	187	7.2587742	371.0395926	188

Dominican Republic	0.619388201	0.3911325	8.3451941	70	7.238945	149.9570246	69
Ecuador	0.661017053	0.3912778	6.4820607	52	7.2998643	111.320135	52
Egypt	0.606787094	0.3885999	1.8701803	18	7.2728861	35.585248	19
El Salvador	0.563775188	0.3923732	2.30187	22	7.4313509	43.5034705	22
Equatorial Guinea	0.657857456	0.3903954	17.766026	170	7.2862827	326.9289684	171
Eritrea	0.403863943	0.4208487	9.5496937	79	8.4973542	171.9156053	82
Estonia	0.844917787	0.3886558	17.3254236	165	7.275842	320.1406368	168
Eswatini	0.585459713	0.3926509	10.9657706	104	7.2529485	199.6486681	111
Ethiopia	0.358823295	0.4210071	5.5156909	46	8.5114913	96.7499139	46
Fiji	0.675051631	0.3899222	18.8365955	175	7.2780687	320.8294901	170
Finland	0.859831368	0.3901135	19.3060702	176	7.2345824	328.232761	174
France	0.838364875	0.3891529	9.9274246	87	7.2634385	168.6714756	80
Gabon	0.634691393	0.3908536	20.0749654	177	7.2263494	370.3429196	185
Gambia	0.40971416	0.4225325	12.6366259	135	8.5119857	228.5608473	136
Georgia	0.732473604	0.3891385	14.0233009	144	7.2942796	284.7271679	158
Germany	0.902957091	0.3910485	11.4582975	117	7.277753	207.1042128	118
Ghana	0.56493039	0.3909779	14.9623922	152	7.324638	270.6497817	152
Greece	0.791854408	0.3917949	21.0147869	183	7.2757348	386.9575003	190
Greenland	0.826210336	0.3891726	9.6387449	81	7.2688284	172.8019548	84
Grenada	0.668993028	0.3906883	24.6330906	195	7.2743112	445.2233726	200
Guam	0.803982203	0.3899375	7.7649861	67	7.2149838	161.3720193	74
Guatemala	0.539972424	0.4036476	1.7920886	15	7.9933581	31.9254354	14
Guinea	0.336401293	0.4206705	9.766326	84	8.5317505	175.5627928	89
Guinea-Bissau	0.353109621	0.4215762	11.4274207	116	8.4988206	210.3774185	122
Guyana	0.650812335	0.3897652	18.0675265	174	7.2099587	342.3340905	177
Haiti	0.448278285	0.4205201	12.2217727	128	8.523131	219.9542834	128
Honduras	0.513037248	0.420859	6.0909043	50	8.5079358	108.1315934	50
Hungary	0.790754768	0.3922436	14.4978692	147	7.2077575	287.8994328	160
Iceland	0.87636168	0.3915577	14.110271	145	7.2539818	223.5312648	131

India	0.575401649	0.3890165	6.0581634	49	7.2497568	110.3937255	51
Indonesia	0.656868336	0.3895358	5.2363711	42	7.2680299	85.4466394	40
Iran (Islamic Republic of)	0.697207398	0.3892203	2.2941406	21	7.2010064	41.3796602	21
Iraq	0.662626231	0.3907597	1.4027594	9	7.2501911	29.3154977	12
Ireland	0.87375385	0.390766	15.9926294	162	7.2456986	248.1395101	144
Israel	0.809011652	0.3912971	6.8292337	57	7.2709152	111.5599771	53
Italy	0.805773534	0.3906886	11.4193232	112	7.271107	195.4276931	103
Jamaica	0.683263064	0.3900348	9.3120326	77	7.2713037	165.2936913	78
Japan	0.871241813	0.3925228	26.7071356	198	7.3029496	438.5526647	199
Jordan	0.725307227	0.3901409	4.3426259	35	7.3180277	83.4773086	39
Kazakhstan	0.725144495	0.3906208	10.9161188	102	7.2920904	220.8983373	129
Kenya	0.523768077	0.4214609	9.1230155	75	8.5032663	160.7876124	73
Kiribati	0.527186583	0.4208417	1.834386	17	8.5110812	33.2986227	17
Kuwait	0.846651055	0.390195	3.9853478	33	7.2592466	73.5229906	34
Kyrgyzstan	0.603979328	0.3903594	3.459796	29	7.21647	69.0322755	32
Lao People's Democratic Republic	0.489136091	0.4214631	4.5755979	37	8.4787258	75.3621374	35
Latvia	0.830663516	0.3901734	15.059644	155	7.2843456	296.2857323	163
Lebanon	0.744746351	0.3912821	10.7811902	97	7.2812647	192.1397705	102
Lesotho	0.510393066	0.4213455	9.8918847	86	8.4912456	174.1287632	87
Liberia	0.352442452	0.4209423	10.1110488	91	8.5254593	179.1303178	92
Libya	0.725771399	0.3911492	1.2066342	6	7.2118823	23.4726851	6
Lithuania	0.856484049	0.3902008	15.4398181	159	7.2481121	304.5794948	166
Luxembourg	0.884428955	0.392213	12.1854229	127	7.221434	200.5773718	114
Madagascar	0.400246943	0.4213156	12.5308652	132	8.5068672	230.2094095	137
Malawi	0.384553634	0.4215949	9.190779	76	8.4914602	171.5380277	81
Malaysia	0.742523828	0.3921129	22.0591568	191	7.2468042	361.6747103	183
Maldives	0.650886627	0.3927682	2.8429121	24	7.2546421	43.7072775	23
Mali	0.268579941	0.4202403	5.9233813	47	8.5370251	105.2503401	49
Malta	0.801585034	0.3910717	6.7876438	55	7.2359293	117.3924542	55

Marshall Islands	0.574091128	0.3904963	14.5192141	148		7.3639832	254.701167	147
Mauritania	0.4989451	0.4210562	12.4964197	131		8.5197833	218.1027183	125
Mauritius	0.718260446	0.3904679	4.2999807	34		7.2928393	71.2248162	33
Mexico	0.664575304	0.3907183	2.8788281	25		7.1964922	50.9312393	25
Micronesia (Federated States of)	0.587534967	0.3886592	17.7845039	171		7.2615846	299.1558361	164
Monaco	0.908262831	0.3886754	28.0052827	201		7.2620029	449.9933392	201
Mongolia	0.617621565	0.3927182	3.1319805	27		7.2796156	59.7099531	29
Montenegro	0.795800584	0.3910635	45.9588085	203		7.2088659	828.2291977	203
Morocco	0.562698301	0.3933214	1.8126763	16		7.3136782	34.1105445	18
Mozambique	0.326462614	0.4205843	12.0638081	125		8.5290711	226.2291894	134
Myanmar	0.53390084	0.4214772	4.741294	40		8.5111948	78.0574735	37
Namibia	0.617564872	0.3903915	12.9031074	138		7.2260139	219.192273	127
Nauru	0.625177834	0.3938369	31.857458	202		7.298811	513.9601608	202
Nepal	0.433174635	0.420084	6.056624	48		8.5059677	103.2132405	48
Netherlands	0.888464256	0.391226	20.590357	181		7.244481	312.2827542	167
New Zealand	0.849442499	0.3884372	21.8476791	189		7.2855285	347.6037442	179
Nicaragua	0.523958472	0.419832	1.4000704	8		8.5042991	25.6413773	10
Niger	0.168072774	4.5626538	1.8749918	20		86.63415	25.3310695	9
Nigeria	0.503390833	0.4203174	12.07059	126		8.5382839	202.1099295	115
Niue	0.72622205	0.3895574	16.6901303	163		7.2787547	281.8607275	155
North Macedonia	0.750629703	0.3911766	14.007141	143		7.262631	249.3882883	145
Northern Mariana Islands	0.771535213	0.3932316	17.4802328	166		7.2763507	292.1418322	162
Norway	0.91613281	0.3881446	26.7910651	199		7.2359408	424.0887565	196
Oman	0.773391602	0.3891075	2.4312433	23		7.289399	49.7821352	24
Pakistan	0.504028689	0.419048	7.5361024	62		8.5578274	130.9816045	60
Palau	0.754046931	0.3895854	14.6435753	149		7.2811172	242.2816296	142
Palestine	0.631011665	0.3891195	3.5709638	30		7.2646104	66.8290707	31
Panama	0.708864828	0.3897227	8.9223771	72		7.2223767	164.5161068	77
Papua New Guinea	0.417797443	0.4220358	7.6474875	63		8.4862245	137.257595	62

Paraguay	0.635718099	0.3929647	14.7662769	151		7.2737267	288.6522496	161
Peru	0.662054037	0.3904672	2.9967845	26		7.2519369	53.2638021	26
Philippines	0.651219329	0.3909525	6.7262183	54		7.2485643	116.7632397	54
Poland	0.812042809	0.3916492	18.0202732	173		7.2071059	327.747666	173
Portugal	0.744151851	0.392024	7.702141	64		7.2781543	142.1403608	65
Puerto Rico	0.825525847	0.3914544	4.636472	38		7.2749096	78.2789249	38
Qatar	0.846860584	0.3901736	4.6537867	39		7.2510523	87.3139839	41
Republic of Korea	0.886675267	0.3912304	10.1678353	92		7.2285998	152.68013	71
Republic of Moldova	0.732214875	0.3935048	7.148403	59		7.3028995	149.6738343	68
Romania	0.768453864	0.3906418	9.0308779	74		7.2641381	184.8250458	95
Russian Federation	0.808536005	0.3882077	21.6854566	186		7.2540944	421.5644583	195
Rwanda	0.435588706	0.4205948	9.4047943	78		8.5190382	166.1749612	79
Saint Kitts and Nevis	0.754987055	0.392462	13.3835598	140		7.2352212	233.1369643	138
Saint Lucia	0.672509735	0.3917202	27.2825341	200		7.2484336	428.9132186	197
Saint Vincent and the Grenadines	0.637195963	0.3918496	11.1159395	109		7.2682993	186.2836254	97
Samoa	0.593392769	0.3912699	14.1179046	146		7.2149328	245.8799944	143
San Marino	0.888005474	0.388949	8.9442178	73		7.3187973	152.2504457	70
Sao Tome and Principe	0.505413747	0.4205665	11.8634843	121		8.5271804	208.4655089	119
Saudi Arabia	0.815143493	0.3909015	0.5927929	2		7.2581743	11.3348134	2
Senegal	0.408054193	0.4215448	11.0612712	108		8.5125434	196.0066571	105
Serbia	0.792416294	0.3910821	21.3649082	184		7.2682989	404.4845056	192
Seychelles	0.730150775	0.3902281	5.3450035	43		7.2658732	88.2398371	42
Sierra Leone	0.358665881	0.4206131	9.689448	83		8.4885246	175.1499045	88
Singapore	0.856097766	0.3934265	11.4222458	114		7.2462433	186.449098	98
Slovakia	0.81061053	0.391284	11.3818726	111		7.2997157	224.0387854	133
Slovenia	0.842430731	0.3908054	11.4235355	115		7.2493604	202.4830705	116
Solomon Islands	0.429360316	0.4203348	10.0217524	89		8.5212023	163.431825	75
Somalia	0.077688109	4.5338994	0	1		86.2375796	0	1
South Africa	0.679626598	0.3898044	11.420831	113		7.1955294	197.5039473	108

South Sudan	0.278371125	0.4205952	6.9079009	58	8.5117405	126.9375643	57
Spain	0.769283698	0.3909421	10.9965033	106	7.236074	199.4636466	110
Sri Lanka	0.701534935	0.3901925	1.6145918	11	7.2412717	24.7068323	8
Sudan	0.541949735	0.4011599	1.2271443	7	7.9239064	23.5845826	7
Suriname	0.633665739	0.3897079	10.5593057	95	7.2465589	195.7848337	104
Sweden	0.886880299	0.390709	21.5038715	185	7.2792885	346.8572202	178
Switzerland	0.933059111	0.3907067	12.4512058	130	7.2320943	197.5740646	109
Syrian Arab Republic	0.623004075	0.3918124	1.6728201	12	7.2679129	32.5126204	15
Taiwan (Province of China)	0.874747053	0.3908452	10.8849422	101	7.30748	200.0811226	113
Tajikistan	0.541511187	0.4050048	0.8796536	3	7.936208	16.3446083	3
Thailand	0.682547933	0.3914706	10.2662709	93	7.2804415	160.6645564	72
Timor-Leste	0.444667619	0.4213755	3.6398657	31	8.4890093	58.6629348	27
Togo	0.408533695	0.421451	11.8576272	120	8.4855497	216.5644856	124
Tokelau	0.686425621	0.3897361	17.5105978	167	7.2605303	284.8806682	159
Tonga	0.626349936	0.3898949	15.7415208	161	7.2523482	277.0815441	154
Trinidad and Tobago	0.768763254	0.3914721	20.9651382	182	7.2609301	370.8842382	187
Tunisia	0.682432216	0.3908497	1.8739021	19	7.2268211	32.8205412	16
Turkey	0.712692673	0.3914226	10.8750191	99	7.3092264	214.3563722	123
Turkmenistan	0.682160776	0.3881685	8.7282883	71	7.2484607	177.0489016	90
Tuvalu	0.576620529	0.3912846	15.1874707	157	7.3094911	256.7308116	148
Uganda	0.423261181	0.4221859	8.0794211	68	8.5204196	144.4569133	67
Ukraine	0.760773913	0.3892394	10.9242039	103	7.229382	234.8272742	141
United Arab Emirates	0.849317734	0.3929818	7.4826432	61	7.2205724	140.6824619	64
United Kingdom	0.859000182	0.3898223	22.3462178	192	7.2522209	339.6913237	176
United Republic of Tanzania	0.446568273	0.4211614	12.9108785	139	8.5368574	234.6386313	140
United States of America	0.862448354	0.3889484	9.8735387	85	7.2642392	173.5643582	86
United States Virgin Islands	0.821830853	0.3900181	10.5669219	96	7.2711036	172.322188	83
Uruguay	0.719283445	0.3902834	22.7962203	193	7.2585351	431.1260309	198
Uzbekistan	0.662621694	0.3930282	5.0830222	41	7.2746878	92.6394257	44

Vanuatu	0.473100706	0.4214391	10.878415	100	8.5044387	188.9681444	100
Venezuela (Bolivarian Republic of)	0.596513059	0.3902914	9.6538998	82	7.2755397	173.1466802	85
Viet Nam	0.627933721	0.3887632	5.4829201	45	7.212528	89.5752916	43
Yemen	0.450376375	0.4216043	1.19729	5	8.5008195	23.1137234	5
Zambia	0.505948954	0.4209507	17.1718179	164	8.4918373	320.511065	169
Zimbabwe	0.473819486	0.4215704	20.3785274	178	8.5318596	363.3165442	184

ASMR, age-standardized mortality rate; ASDR, age-standardized disability-adjusted life years rate; AA, aortic aneurysm; SDI, sociodemographic index; DALYs, disability-adjusted life years.

Table 8. Forecasts for the number of deaths, ASMR, number of DALYs, and ASDR for AA from 2022 to 2050.

Location	Year	Death number (95% CI)	ASMR (95% CI)	DALYs number (95% CI)	ASDR (95% CI)
	2022	152383(147522-157245)	9.92(9.60-10.23)	2575662(2491022-2660302)	167.60(162.10-173.11)
	2023	154882(148870-160893)	9.80(9.42-10.18)	2621966(2517273-2726659)	165.93(159.31-172.55)
	2024	157463(150379-164548)	9.69(9.26-10.13)	2669034(2545180-2792888)	164.28(156.66-171.90)
	2025	160036(151909-168164)	9.59(9.10-10.07)	2715540(2572578-2858502)	162.65(154.09-171.21)
	2026	162536(153372-171700)	9.48(8.95-10.02)	2760514(2597973-2923055)	161.05(151.57-170.53)
	2027	164915(154708-175122)	9.38(8.81-9.96)	2802841(2619913-2985769)	159.50(149.09-169.91)
	2028	167170(155915-178425)	9.29(8.67-9.91)	2842297(2638048-3046546)	157.94(146.60-169.29)
	2029	169366(157040-181692)	9.20(8.53-9.87)	2879796(2652875-3106718)	156.38(144.06-168.70)
Global	2030	171492(158055-184929)	9.11(8.39-9.82)	2915439(2664055-3166822)	154.82(141.48-168.17)
	2031	173559(158953-188164)	9.02(8.26-9.78)	2949478(2671413-3227543)	153.28(138.83-167.73)
	2032	175553(159710-191395)	8.93(8.13-9.74)	2981832(2674512-3289153)	151.76(136.12-167.40)
	2033	177435(160286-194583)	8.85(8.00-9.70)	3011673(2672461-3350886)	150.21(133.29-167.13)
	2034	179213(160674-197753)	8.76(7.86-9.67)	3039188(2665155-3413222)	148.64(130.35-166.93)
	2035	180956(160913-200999)	8.68(7.72-9.64)	3065335(2653113-3477557)	147.06(127.28-166.84)
	2036	182693(161012-204374)	8.60(7.58-9.62)	3091015(2636785-3545246)	145.48(124.10-166.86)
	2037	184451(160973-207929)	8.52(7.43-9.60)	3116775(2616316-3617235)	143.91(120.80-167.01)
	2038	186238(160792-211684)	8.43(7.28-9.58)	3143122(2591972-3694271)	142.30(117.35-167.26)

2039	188046(160440-215652)	8.35(7.12-9.57)	3169750(2563235-3776265)	140.67(113.75-167.59)
2040	189819(159847-219791)	8.26(6.95-9.56)	3195610(2528995-3862225)	139.02(110.02-168.02)
2041	191517(158959-224075)	8.17(6.78-9.56)	3219824(2488366-3951283)	137.38(106.17-168.58)
2042	193052(157684-228420)	8.08(6.60-9.57)	3241201(2440306-4042097)	135.73(102.20-169.27)
2043	194465(156050-232881)	8.00(6.42-9.58)	3260648(2385557-4135739)	134.08(98.10-170.07)
2044	195789(154067-237512)	7.91(6.22-9.59)	3278967(2324656-4233278)	132.41(93.87-170.95)
2045	196951(151664-242239)	7.81(6.02-9.61)	3294713(2256535-4332890)	130.73(89.53-171.92)
2046	197877(148774-246979)	7.72(5.81-9.64)	3306661(2180459-4432863)	129.05(85.10-173.00)
2047	198487(145336-251638)	7.63(5.59-9.67)	3313450(2095748-4531152)	127.37(80.56-174.18)
2048	198770(141351-256189)	7.54(5.36-9.71)	3315298(2002958-4627639)	125.70(75.94-175.45)
2049	198822(136897-260747)	7.44(5.12-9.76)	3313782(1903402-4724161)	124.02(71.23-176.80)
2050	198666(131996-265337)	7.34(4.88-9.81)	3309386(1797669-4821103)	122.34(66.45-178.22)

ASMR, age-standardized mortality rate; DALYs, disability-adjusted life years; ASDR, age-standardized disability-adjusted life years rate; AA, aortic aneurysm; CI, confidence interval.

Table 9. The prediction accuracy validation of the Bayesian age-period-cohort mode

Indicator	MAE	MAPE (%)	Fit accuracy (%)
Deaths	11,937.04	11.612	88.388
DALYs	97,032.45	5.279	94.721
ASMR	0.1109	0.921	99.079
ASDR	0.9032	0.445	99.555

MAE, mean absolute error; MAPE, mean absolute percentage error

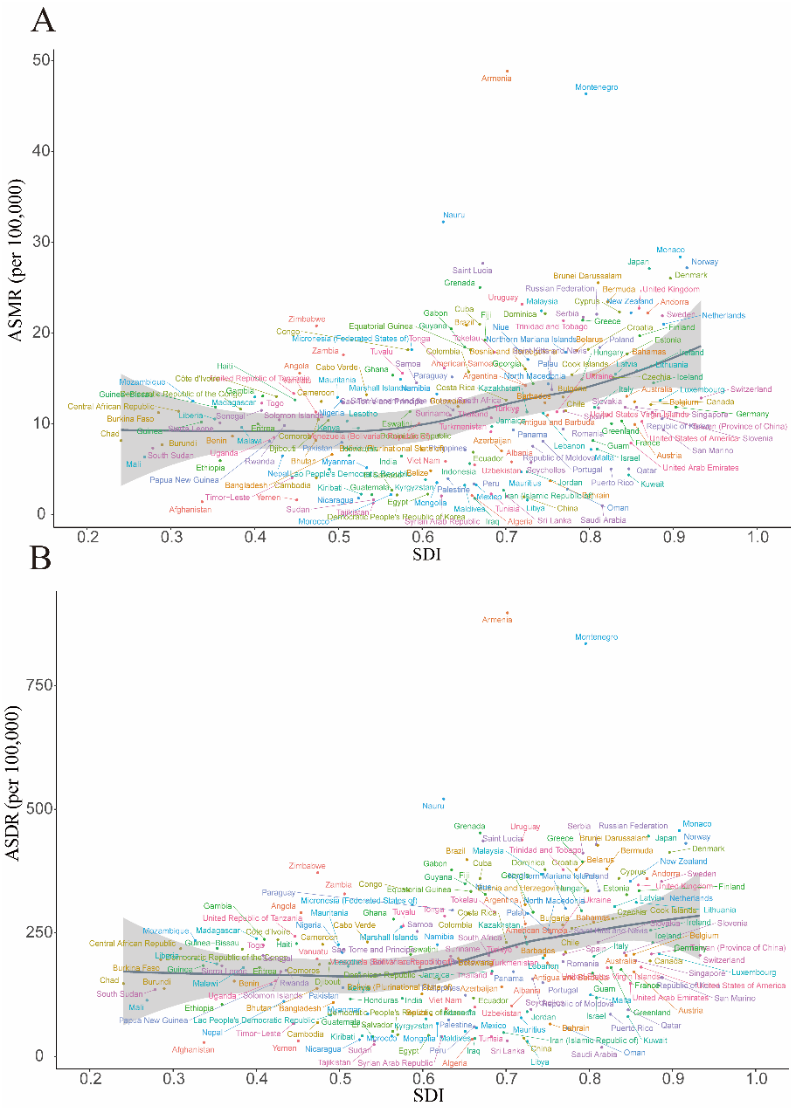

Figure 1. ASMR (A) and ASDR (B) of AA among individuals aged 55 and older from 1990 to 2021 in 204 countries and territories classified by SDI.

ASMR: age-standardized mortality rate; ASDR: age-standardized disability-adjusted life years rate; SDI: sociodemographic index.

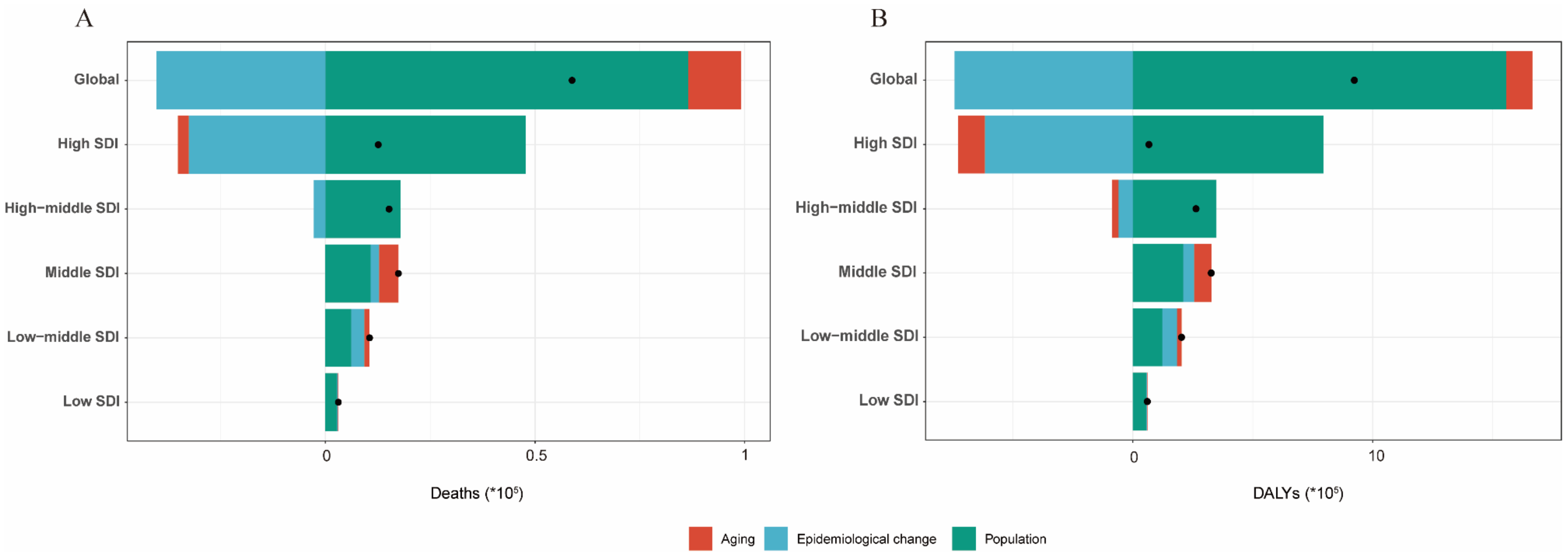

Figure 2. Impact of aging, epidemiological changes, and population growth on AA deaths and DALYs globally and across SDI regions from 1990 to 2021. AA: aortic aneurysm; DALYs: disability-adjusted life years; SDI: sociodemographic index.

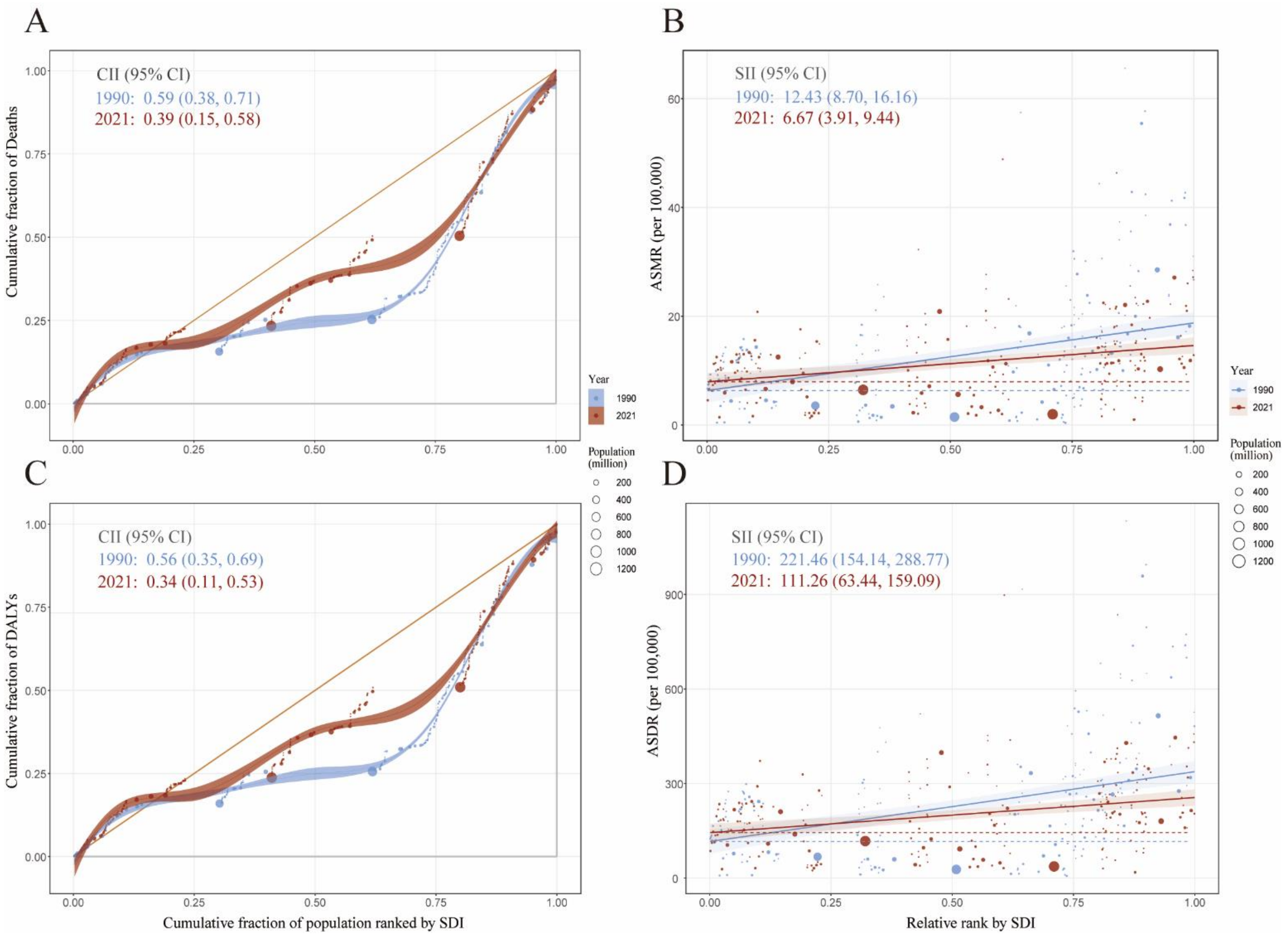

Figure 3. SII and CII of ASMR (A and B) and ASDR (C and D) for global AA in 1990 and 202.

SII: slope index of inequality; CII: concentration index of inequality; ASMR: age-standardized mortality rate; ASDR: age-standardized disability-adjusted life years rate; AA: aortic aneurysm.

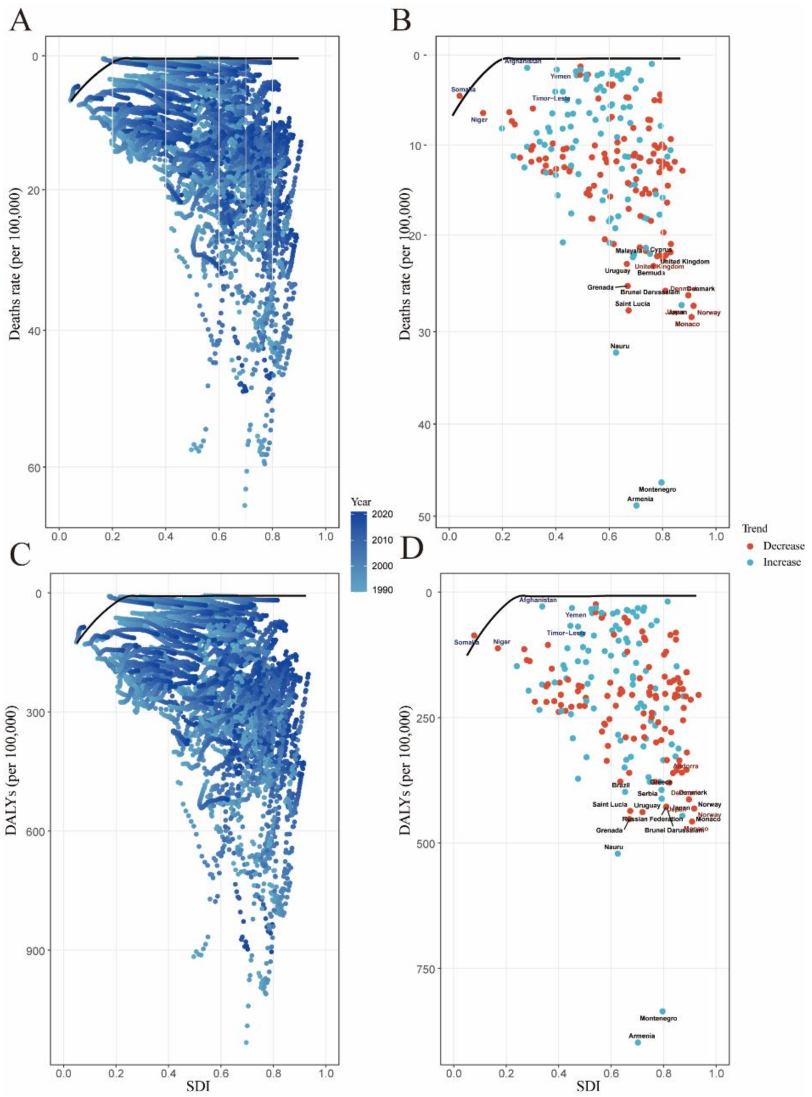

Figure 4: Frontier analysis based on SDI, ASMR and ASDR for AA across 204 countries and territories.

SDI: sociodemographic index; ASMR: age-standardized mortality rate; ASDR: age-standardized disability-adjusted life years rate; AA: aortic aneurys.
